# SPE–UHPLC–MS/MS Method for Simultaneous Quantification of 50 Pesticide Biomarkers Across Nine Current-Use Chemical Classes in Human Urine

**DOI:** 10.3390/jox16020067

**Published:** 2026-04-13

**Authors:** Ravikumar Jagani, Jasmin Chovatiya, Hiraj Patel, Sandipkumar Teraiya, Divya Pulivarthi, Syam S. Andra

**Affiliations:** Institute for Exposomic Research, Department of Environmental Medicine, Icahn School of Medicine at Mount Sinai, New York, NY 10029, USA

**Keywords:** pesticide biomarkers, human biomonitoring, UHPLC–MS/MS, solid-phase extraction, multi-class analysis, Exposomics

## Abstract

A comprehensive ultra-high-performance liquid chromatography-tandem mass spectrometry (UHPLC-MS/MS) method was developed for the simultaneous quantification of 50 pesticide biomarkers across nine current-use chemical classes in human urine. These classes include organophosphorus insecticides (which encompass dialkyl phosphates and specific metabolites), pyrethroid insecticides, fungicides, neonicotinoid insecticides, herbicides, insect repellents, organochlorine pesticide metabolites, and plant growth regulators. The method employs solid-phase extraction (SPE) for sample preparation, requiring only 0.2 mL of urine. Chromatographic separation was optimized using a Hypersil Gold AQ column, achieving a total run time of 18 min. Mass spectrometric detection utilized polarity switching in electrospray ionization mode with multiple reaction monitoring. Method validation demonstrated satisfactory linearity (R^2^ > 0.99), high sensitivity with limits of detection ranging from 0.01 to 0.88 ng/mL, and extraction efficiencies between 85% and 113%. Precision and accuracy were within acceptable ranges, with relative standard deviations generally below 15%. The method’s robustness was confirmed through participation in external quality assessment schemes. Application to real samples revealed significant inter-individual variability in pesticide biomarker concentrations, with total measured biomarker levels ranging from 89 to 1242 ng/mL across the 10 individuals analyzed. This method offers comprehensive coverage of current-use pesticide chemical classes, including 30 biomarkers from the U.S. National Health and Nutrition Examination Survey (NHANES) biomonitoring program, and demonstrates improved sensitivity and broader analyte coverage compared to existing methods. The developed assay provides a valuable tool for large-scale biomonitoring studies and environmental health research.

## 1. Introduction

Pesticides are ubiquitous environmental contaminants used extensively in agriculture, gardening, and pest control [1]. Their widespread application has led to frequent detection of pesticides and their metabolites in human biological samples, raising concerns about potential health impacts [1]. Human exposure to pesticides occurs through inhalation, ingestion, and dermal contact, after which these compounds are typically metabolized in the liver and excreted via urine [1]. Urine has become the preferred matrix for human biomonitoring (HBM) of pesticide exposure due to its non-invasive collection, availability in larger quantities than blood, and role as the main excretion route for many pesticides and their metabolites [1].

Numerous epidemiological studies have linked pesticide exposure to adverse health outcomes, particularly in vulnerable populations such as pregnant women and children [1]. Neurotoxic pesticides like organophosphates, pyrethroids, carbamates or thiocarbamates (fungicides), and neonicotinoids can disrupt normal neuronal functions [2], while others like triazoles (fungicides), herbicides, organochlorine pesticides, and insect repellents may interfere with the endocrine system [3,4], and plant growth regulators show reproductive and developmental toxicity [5]. These health concerns underscore the critical need for comprehensive HBM methods to assess human exposure to multiple pesticide classes simultaneously.

Traditionally, HBM methods have focused on one or two pesticide classes [6]. However, there is growing interest in multitarget methods capable of characterizing several pesticide classes and their metabolites in a single analysis [6]. This approach allows for a more comprehensive assessment of human exposure to multiple pesticide types and classes [7,8,9,10,11]. Recent studies have optimized methodologies to include 13 to 39 different pesticides, encompassing both currently used and banned compounds, from 3 to 5 pesticide classes (Table 1). The method by Bustamante et al. [12] covers 23 analytes in 4 classes, while Marin-Saez et al. [13] analyze 39 analytes in 5 classes. Larose et al. [14], Gao et al. [15], and Zhu et al. [16] cover 13, 26, and 31 analytes, respectively, in 3 to 5 pesticide classes.

**Figure 1:**
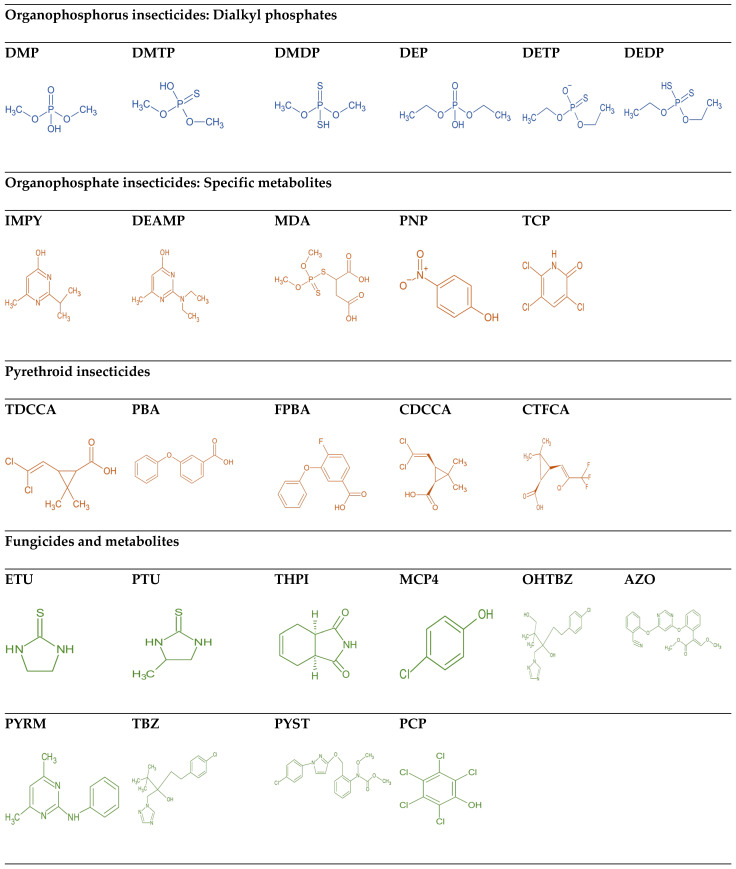

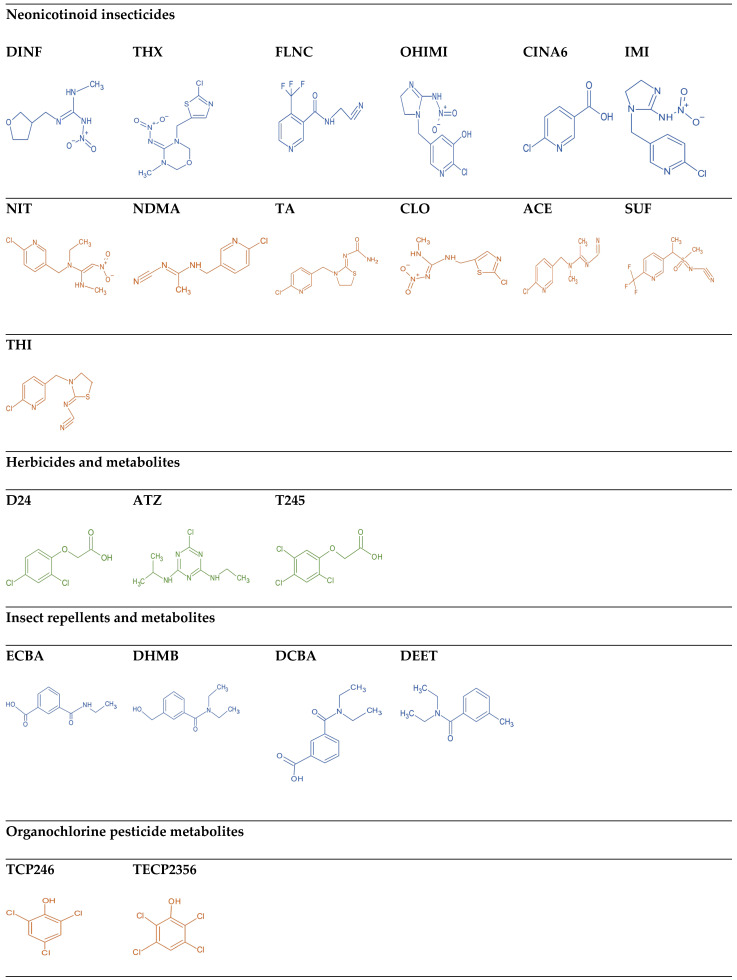

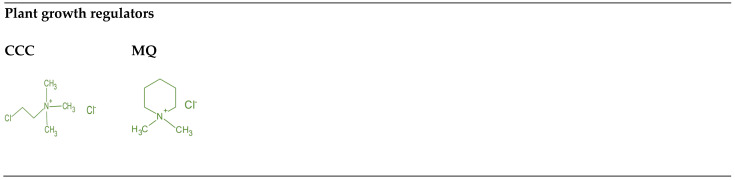
The chemical structures of the studied pesticides and metabolites, categorized by their chemical classes.

Using ultra-high-performance liquid chromatography (UHPLC) and tandem mass spectrometry (MS/MS), the current study aims to develop and optimize a multiclass pesticide urinary assay that covers a range of biomarkers of exposures to current-use pesticides from nine chemical classes currently of interest in the National Health and Nutrition Examination Survey (NHANES) biomonitoring and others of emerging health concern (Figure 1, Table 2). This includes organophosphorus insecticides (dialkyl phosphates and specific metabolites), pyrethroids, fungicides, neonicotinoids, herbicides, insect repellents, organochlorine pesticides, and plant growth regulators. Target selection criteria for non-NHANES biomarkers were predefined as follows: (1) evidence of contemporary agricultural or residential use (current-use status), (2) published or suspected human exposure and toxicological concern in recent biomonitoring studies, and (3) availability of analytical reference standards or participation in proficiency testing to enable quantification. These criteria align with other multiclass method development efforts that prioritize analytes balancing public-health relevance and analytical feasibility [12,15,17]. The term ‘emerging health concerns’ herein refers specifically to compounds meeting at least two of these criteria but not yet included in routine national surveys.

Sample preparation is a crucial step in our method, employing solid-phase extraction (SPE) to efficiently extract and concentrate pesticide biomarkers from urine samples. This approach allows for effective handling of multiple components with varying chemical properties, enhancing the method’s sensitivity and selectivity. The optimized SPE procedure, using Oasis HLB 96-well plates, enables high-throughput sample processing essential for large-scale biomonitoring studies. Our method demonstrates high sensitivity, accuracy, and precision while allowing for high sample throughput to support large-scale biomonitoring studies and environmental health research. The resulting method will serve as a valuable tool for facilitating more accurate health risk assessments and informing public health policies aimed at reducing pesticide-related health risks. Such high-throughput, low-volume assays are particularly well-suited for large-scale epidemiologic studies and pediatric cohorts, where limited sample volumes and high participant numbers necessitate sensitive, rapid analyses [18,19,20,21,22]. Comparable multiclass SPE-LC-MS/MS methods have been shown to support population biomonitoring with similar sample-volume efficiencies and throughput, reinforcing the utility of the present assay for exposomic applications [13,15,17].

**Table 2:** Pesticides, parent compounds and urinary metabolites, by abbreviations, parent chemicals, and metabolites by chemical class.

#	Chemical Class	Parent Chemical	Biomarker (Analyte) Full Name	CAS No.	Abbreviation	NHANES ^a^	NIST SRM ^b^	PT ^c^	Reference(s) ^d^
1	Organophosphorus insecticides: Dialkyl phosphates (Non-specific metabolites)	Organophosphate insecticides (multiple)	Dimethylphosphate	813-78-5	DMP	Yes	-	G-EQUAS	Heffernan et al., 2016 [23]
			Dimethylthiophosphate	59401-04-6	DMTP	Yes	-	G-EQUAS	
			Dimethyldithiophosphate	756-80-9	DMDP	Yes	-	G-EQUAS	
			Diethylphosphate	598-02-7	DEP	Yes	-	G-EQUAS	
			Diethylthiophosphate	5871-17-0	DETP	Yes	-	G-EQUAS	
			Diethyldithiophosphate	298-06-6	DEDP	Yes	-	G-EQUAS	
2	Organophosphorus insecticides: Specific metabolites	Parathion, methyl parathion	4-nitrophenol	100-02-7	PNP	Yes	-	G-EQUAS	Heffernan et al., 2016 [23]; Noren et al., 2020 [24]
		Chlorpyrifos, chlorpyrifos-methyl	3,5,6-trichloro-2-pyridinol	6515-38-4	TCP	Yes	-	G-EQUAS	
		Malathion	2-[dimethoxyphosphorothioyl) sulfanyl] succinic acid	1190-28-9	MDA	Yes	-	-	
		Diazinon	2-isopropyl-4-methyl-pyrimidinol	2814-20-2	IMPY	Yes	-	-	
		Pirimiphos	2-(diethylamino)-6-methylpyrimidin-4-ol	42487-72-9	DEAMP	-	-	-	
3	Pyrethroid insecticides	Permethrin, cypermethrin, cyfluthrin	trans-dichlorovinyl-dimethylcyclopropane carboxylic acid	59042-50-1	TDCCA	Yes	-	G-EQUAS	Heffernan et al., 2016 [23]; Noren et al., 2020 [24]
			cis-dichlorovinyl-dimethylcyclopropane carboxylic acid	59042-49-8	CDCCA	Yes	-	G-EQUAS	
		Permethrin, cypermethrin, deltamethrin, resmethrin, fevalerate, phenothrin, cyphenothrin and lambda-cyhalothrin.	3-phenoxybenzoic acid	3739-38-6	PBA	Yes	-	G-EQUAS	
		Cyfluthrin	4-fluoro-3-phenoxybenzoic acid	77279-89-1	FPBA	Yes	-	G-EQUAS	
		Bifenthrin, lambda-cyhalothrin	3-(2-chloro-3,3,3-trifluoroprop-1-enyl)-2,2-dimethylcyclopropanecarboxylic acid	72748-35-7	CTFCA	-	-	G-EQUAS	
4	Fungicides and metabolites	Pentachlorophenol	Pentachlorophenol	87-86-5	PCP	Yes	-	G-EQUAS	Noren et al., 2020 [24]
		Chlorobenzene	4-chlorophenol	106-48-9	MCP4	-	-	-	
		Tebuconazole	Hydroxy tebuconazole	212267-64-6	OHTBZ	-	-	-	
		Mancozeb and several ethylene bis-dithiocarbamate (EBDC) fungicides	Ethylene thiourea	96-45-7	ETU	Yes	-	-	
		Propineb	Propylene thiourea	2122-19-2	PTU	Yes	-	-	
		Pyrimethanil	Pyrimethanil	53112–28–0	PYRM	-	-	-	
		Tebuconazole	Tebuconazole	107534-96-3	TBZ	-	-	-	
		Captan	cis-1,2,3,6-tetrahydrophthalimide	1469-48-3	THPI	-	-	-	
		Azoxystrobin	Azoxystrobin	131860-33-8	AZO	-	-	-	
		Pyraclostrobin	Pyraclostrobin	175013-18-0	PYST	-	-	-	
5	Neonicotinoid insecticides	Acetamiprid, imidacloprid, nitenpyram, thiacloprid	6-chloronicotinic acid	5326-23-8	CINA6	-	-	G-EQUAS	Wrobel et al., 2023 [25]
		Acetamiprid	Acetamiprid	135410-20-7	ACE	Yes	-	G-EQUAS, OSEQAS	
			N-desmethyl-acetamiprid	190604-92-3	NDMA	Yes	-	G-EQUAS, OSEQAS	
		Imidacloprid	Imidacloprid	138261-41-3	IMI	Yes	-	G-EQUAS, OSEQAS	
			5-hydroxyimidacloprid	380912-09-4	OHIMI	Yes	-	OSEQAS	
		Clothianidin	Clothianidin	210880-92-5	CLO	Yes	-	OSEQAS	
		Thiacloprid	Thiacloprid	111988-49-9	THI	Yes	-	OSEQAS	
			Thiacloprid-amide	676228–91–4	TA	-	-	-	
		Thiamethoxam	Thiamethoxam	153719–23–4	THX	-	-	OSEQAS	
		Sulfoxaflor	Sulfoxaflor	946578–00–3	SUF	-	-	OSEQAS	
		Nitenpyram	Nitenpyram	150824-47-8	NIT	-	-	OSEQAS	
		Flonicamid	Flonicamid	158062-67-0	FLNC	-	-	-	
		Dinotefuran	Dinotefuran	165252-70-0	DINF	-	-	OSEQAS	
6	Herbicides and metabolites	2,4-dichlorophenoxyacetic acid	2,4-dichlorophenoxyacetic acid	94-75-7	D24	Yes	-	OSEQAS	Heffernan et al., 2016 [23]
		2,4,5-trichloro phenoxyacetic acid	2,4,5-trichlorophenoxyacetic acid	93-76-5	T245	Yes	-	-	
		Atrazine	Atrazine	1912–24–9	ATZ	Yes	-	-	
7	Insect repellents and metabolites	N,N-diethyl-meta-toluamide	N,N-diethyl-meta-toluamide	134-62-3	DEET	Yes	-	-	Heffernan et al., 2016 [23]
			3-(diethylcarbamoyl) benzoic acid	72236-23-8	DCBA	Yes	-	-	
			3-(ethylcarbamoyl) benzoic acid	126926-33-8	ECBA	Yes	-	-	
			N,N-diethyl-3-(hydroxymethyl) benzamide	72236-22-7	DHMB	-	-	-	
8	Organochlorine pesticide metabolites	Trichlorophenol	2,4,6-trichlorophenol	88-06-2	TCP246	Yes	-	-	Schmied-Tobies et al., 2021 [26]
		Tetrachlorophenol	2,3,5,6-tetrachlorophenol	935-95-5	TECP2356	-	-	-	
9	Plant growth regulators	Chlormequat	Chlormequat	7003-89-6	CCC	-	-	-	Noren et al., 2020 [24]
		Mepiquat	Mepiquat	15302-91-7	MQ	-	-	-	

**^a^** included in at least one of the NHANES cycles conducted between 1999 and 2018. **^b^** availability of a mass fraction value for the pesticide biomarker of interest in the National Institute of Standards and Technology (NIST) Standard Reference Material (SRM) 3672: Organic Contaminants in Smokers’ Urine and/or 3673: Organic Contaminants in Non-Smokers’ Urine. **^c^** offered in the proficiency testing (PT) programs like the G-EQUAS [https://app.g-equas.de/web/ (accessed on 25 February 2026)] and/or the OSEQAS [https://www.inspq.qc.ca/en/ctq/eqas/oqesas/description, (accessed on 25 February 2026)]. **^d^** articles that reference the parent chemical of pesticides and their corresponding urinary biomarkers. #: Numbers.

## 2. Materials and Methods

### 2.1. Chemicals and Reagents

Our method utilizes 50 reference standards and 26 isotope-labeled internal standards (IL-ISTD) for the quantification of pesticide biomarkers. The IL-ISTD were carefully selected to represent the physicochemical properties of our target analytes. Reference standards and IL-ISTD of pesticides and metabolites were acquired from multiple sources, including AA Blocks (San Diego, CA, USA), Enamine (Monmouth Jct., NJ, USA), Cambridge Isotope Laboratories (Tewksbury, MA, USA), Sigma-Aldrich (St. Louis, MO, USA), and Toronto Research Chemicals (North York, ON, Canada). Table 2 and Table S1 provide detailed information on these reference standards and IL-ISTD. Working and intermediate stock solutions of all reference standards and IL-ISTD were prepared in acetonitrile at concentrations of 1 mg/mL and 1 µg/mL, respectively, and stored at −20 °C. All solvents used, including water, acetonitrile, and methanol, were of LC/MS grade, and all other chemicals and reagents were of the highest grade available, obtained from Fisher Scientific (Hampton, NH, USA). For solid-phase extraction (SPE) sample cleanup, an Oasis HLB hydrophilic-lipophilic balanced reversed-phase 96-well plate was purchased from Waters Corporation (Milford, MA, USA). Sample and extract collection and handling were performed using a 96-deep well plate (DWP) with a 2 mL well volume from Eppendorf (Hauppauge, NY, USA). To achieve complete deconjugation of exposure biomarkers of interest, the ALS enzyme BGALA-RO-glucuronidase/arylsulfatase from Helix pomatia was obtained from Sigma-Aldrich, as optimized, and detailed in Jagani et al. [18] A detailed mapping of analytes to isotope-labeled internal standards is provided in Table 3 (matched IL-ISTD vs surrogate). Analytes lacking matched isotopically labeled standards were quantified using surrogate IL-ISTDs selected based on physicochemical similarity (retention time and polarity) and comparable MS/MS fragmentation patterns, an approach used in prior multiclass urinary pesticide and environmental-chemical methods to balance coverage and cost [15,17].

### 2.2. Biological Material

Urine samples for blanks were obtained from Lee Biosolutions, Inc. (Maryland Heights, MO, USA). These samples were collected from healthy children and adults of both genders. Additionally, synthetic urine was sourced from UTAK (Valencia, CA, USA). The urine QC pool was created by combining urine samples obtained from anonymous volunteers. Each sample was individually screened for the analytes of interest in this study before mixing. This process ensured the formation of an unspiked QC urine pool that contains analytes at the lower limit of quantification (LLOQ) levels. This study was conducted in accordance with ethical guidelines and received approval from the Icahn School of Medicine at Mount Sinai (IRB-16-00742) for developing analytical methods using de-identified human specimens that contain no personally identifiable information (PII) or protected health information (PHI).

### 2.3. Chromatographic Conditions

The chromatographic program and mass spectrometric conditions were adapted and modified from Jagani et al.’s report on multiclass analysis of environmental chemical biomarkers in urine [18]. An ExionLC system equipped with a Hypersil Gold AQ analytical column (150 mm × 4.6 mm, 3 μm particle size) and guard column (10 mm × 4.0 mm, 3 μm) from Thermo Scientific (Waltham, MA, USA) was used for the separation of 50 pesticides and their metabolites. The LC system was maintained at 40 °C with a flow rate of 0.5 mL/min. The mobile phase consisted of 0.1% acetic acid in water (A) and a 1:1 ratio of acetonitrile to methanol (B). The gradient elution program was as follows: 0–1.0 min: 5% B; 1.0–6.0 min: linear gradient to 50% B; 6.0–8.0 min: 50% B; 8.0–11.0 min: linear gradient to 95% B; 11.0–14.0 min: 95% B; 14.0–14.2 min: return to 5% B; 14.2–18.0 min: re-equilibration at 5% B. The total LC separation run time was 18.0 min.

### 2.4. Mass Spectrometric Conditions

Mass spectrometric analysis was performed using a SCIEX 7500 Triple Quad system, a Qtrap-ready mass spectrometer (SCIEX, Framingham, MA, USA). The source temperature was set at 350 °C, with nitrogen used as the curtain gas, ion source gas 1, and ion source gas 2. The flow rates were set at 50 psi for curtain gas, 30 psi for ion source gas 1, and 60 psi for ion source gas 2. These optimized gas flow rates and pressures contributed to the method’s high sensitivity and reproducibility. A 10-μL injection volume of the SPE extract was introduced onto the LC column. Data acquisition was conducted simultaneously using electrospray ionization (ESI) in both negative ion and positive ion modes, allowing for comprehensive coverage of pesticide biomarkers with diverse chemical properties. Analyte-specific parameters (declustering potential, entrance potential, collision exit potential, and collision energy) were individually optimized by direct infusion of each compound into the mass spectrometer using a syringe. Detailed MS/MS conditions for each native and labeled analyte are provided in Table 3. Data acquisition, analysis, and processing were performed using SCIEX OS 3.3 software.

### 2.5. Sample Preparation

**Figure 2:**
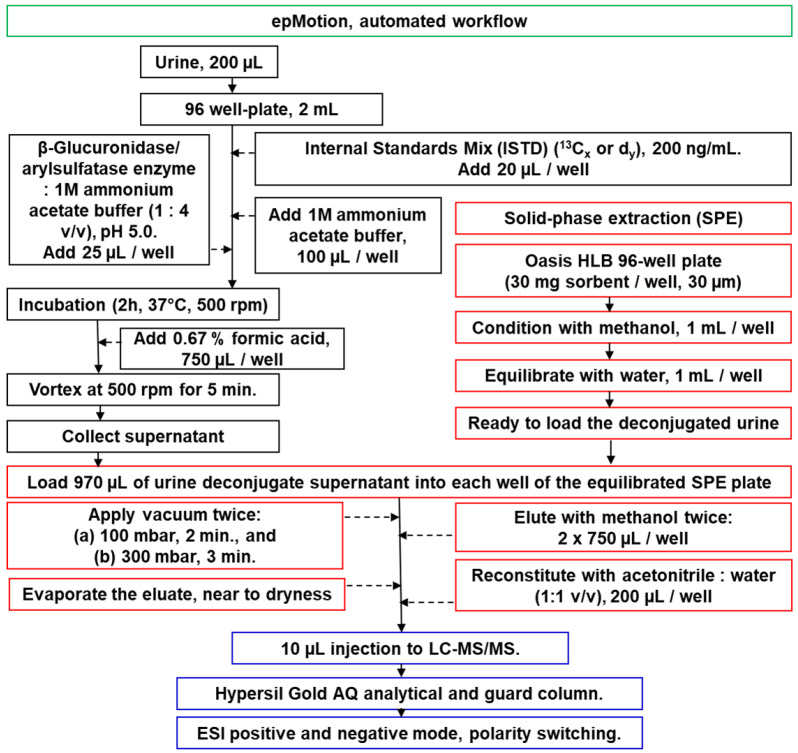
A flowchart optimized for urine cleanup and analysis enables the extraction, separation, and quantification of 50 pesticide chemicals and their metabolites from a single urine aliquot in one assay, while also allowing for customization to include additional analytes or classes of pesticides.

The sample preparation method for analyzing pesticides and metabolites was adapted and modified from Jagani et al. [18] An automated workflow using epMotion 5075vtc (Eppendorf, Hauppauge, NY, USA) was employed for sample preparation (Figure 2). Urine samples (200 µL) were aliquoted into a 96-well plate (2 mL capacity). A mixture of β-glucuronidase/arylsulfatase enzyme and 1M ammonium acetate buffer (1:4 *v*/*v*, pH 5.0) was prepared that gives an enzyme-specific activity of about 100,000 units/mL for β-glucuronidase and 47,500 units/mL for sulfatase. To each well, 20 µL of IL-ISTD mix (13C or deuterium-labeled, 200 ng/mL) and 25 µL of enzyme-buffer mixture were added, followed by 100 µL of 1M ammonium acetate buffer. The plate was incubated at 37 °C for 2 h (500 rpm) on a Mixmate vortexer (Eppendorf, Hauppauge, NY, USA). After incubation, 750 µL of 0.67% formic acid was added, and the plate was vortexed (500 rpm, 5 min). The supernatant (970 µL) was loaded onto a preconditioned and pre-equilibrated Oasis HLB 96-well SPE plate (30 mg sorbent/well, 30 µm). Vacuum was applied twice (100 mbar, 2 min; 300 mbar, 3 min). Analytes were eluted with methanol (2 × 750 µL/well), evaporated near to dryness using a SPE Dry 96 evaporator (Biotage, LLC; Charlotte, NC, USA), and reconstituted with acetonitrile: water (1:1 *v*/*v*, 200 µL/well). 10 µL was injected into the LC-MS/MS system equipped with a Hypersil Gold AQ column, using both negative and positive ESI modes with polarity switching.

### 2.6. Method Validation

Method validation was performed using a pooled urine sample prepared by mixing urine samples collected from anonymous volunteers. This pooled sample served as the basis for our quality control (QC) samples. The concentrations of each analyte in the QC urine pool were characterized (Table 3) and classified as LLOQ or unspiked QC. Low to higher-level QC pools were prepared by spiking the QC urine pool with native standards at three levels (1, 10, and 100 ng/mL). Recovery, linearity, accuracy, precision, limit of detection (LOD), and limit of quantification (LOQ) were determined for all 50 analytes.

LOD and LOQ were calculated as 3S and 10S, respectively, where S is the standard deviation from three replicate analyses of the unspiked QC urine pool, analyzed in ten separate batches. Accuracy was assessed by calculating the extraction efficiencies (EE) of spiked analytes in the two higher-level QC pools using the formula: EE (%) = [measured conc. (ng/mL) * 100]/[QC pool baseline conc. + spiked conc. (ng/mL)]. Precision was evaluated using the coefficient of variation (CV) at each level. Inter-batch accuracy was reported as the relative error (RE) of an expected concentration. Measurement uncertainty (precision) was expressed as the relative standard deviation (RSD).

The method was further validated through participation in proficiency testing (PT) programs conducted by the German External Quality Assessment Scheme for analyses of biological materials (G-EQUAS) [http://www.g-equas.de/ (accessed on 25 February 2026)] and the Canadian External Quality Assessment Scheme for Organic Substances in Urine (CTQ-OSEQAS) [https://www.inspq.qc.ca/en/ctq/eqas/oqesas/description, (accessed on 25 February 2026)]. Finally, to demonstrate its practical application, the validated method was applied to 10 urine samples collected from adult volunteers.

The Mount Sinai laboratory is part of the NIEHS’s Children’s Health Exposure Analysis Resource (CHEAR) consortium [27], which focuses on exposure analysis using both traditional biomonitoring methods and untargeted analysis of the exposome. This consortium has now become the Human Health Exposure Analysis Resource (HHEAR) [28]. The laboratory follows the QA/QC and method validation protocols outlined here [29]. The CHEAR and HHEAR programs are referenced to indicate alignment with established laboratory network practices for quality assurance in human biomonitoring.

## 3. Results and Discussion

### 3.1. Analyte Coverage and Pesticide Classes

The developed method encompasses a comprehensive panel of 50 pesticide biomarkers across 9 current-use chemical classes (Table 2). This broad coverage allows for simultaneous assessment of exposure to multiple pesticide classes, providing a more complete picture of overall pesticide exposure (Table 1). The pesticide classes include organophosphorus insecticides (dialkyl phosphates and specific metabolites), pyrethroid insecticides, fungicides, neonicotinoid insecticides, herbicides, insect repellents, organochlorine pesticide metabolites, and plant growth regulators. Of the 50 biomarkers, 30 are currently included in the NHANES biomonitoring program, indicating their relevance for population-level exposure assessment (Table 2).

### 3.2. Optimization of Chromatographic Separation

**Figure 3:**
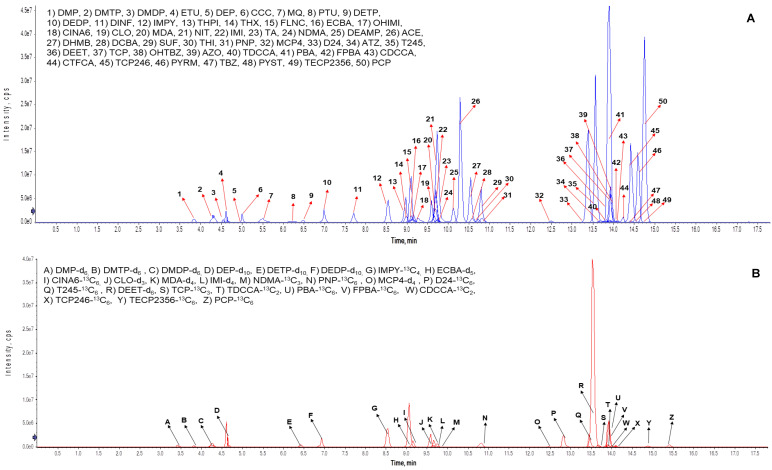
Representative extracted ion chromatograms (EIC) of multiclass pesticide analytes: (**A**) native standard solution and (**B**) stable isotope-labeled internal standard solution.

**Figure 4:**
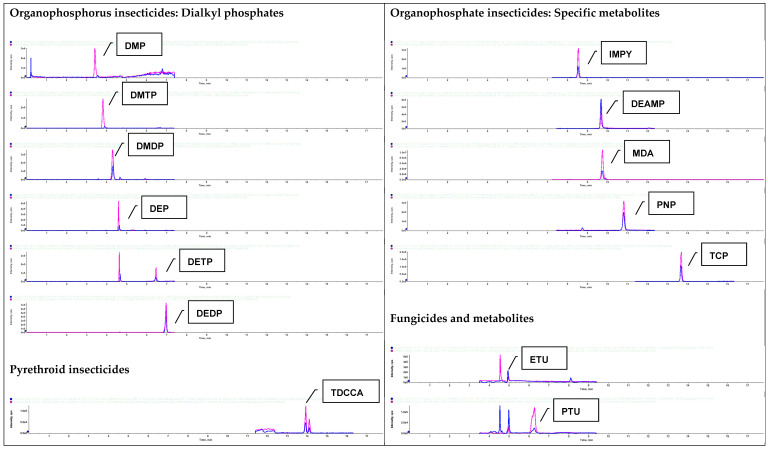

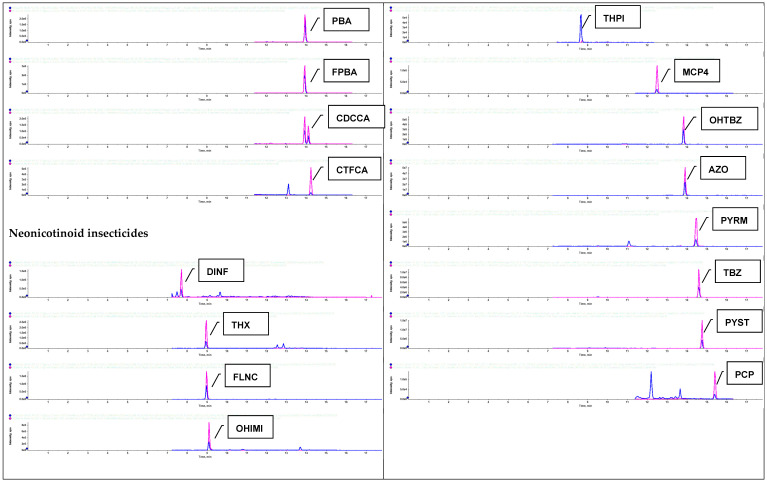

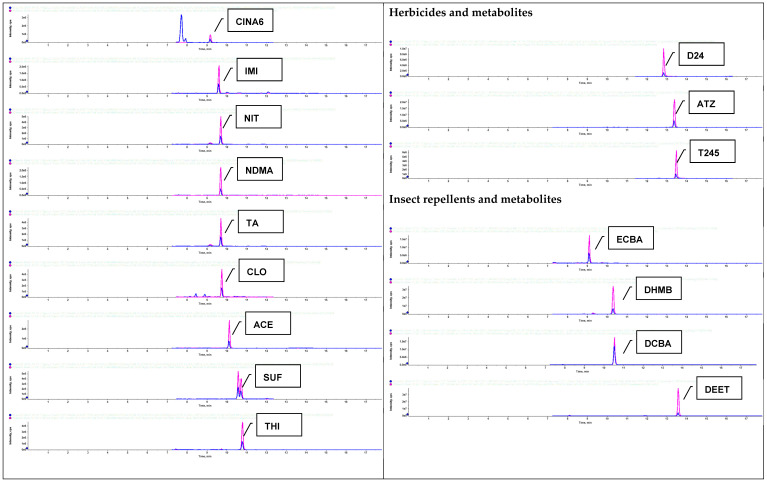

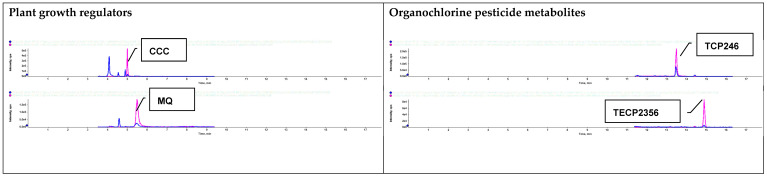
Representative LC-MS/MS MRM ion chromatograms of various classes of pesticides and their metabolites obtained from a single injection of an extract from a representative urine sample (blue line) alongside a 1 ng/mL standard solution (red line).

Several reversed-phase columns were evaluated during method development. Chromatographic separation was optimized using a Thermo Scientific Hypersil Gold AQ column (150 mm × 4.6 mm, 3 μm), which provided excellent separation for the diverse range of pesticide classes (Figure 3). In contrast, Gao et al. [15] opted for a Phenomenex Kinetex XB-C18 column (100 mm × 2.1 mm, 1.7 μm), and Mercier et al. [17] opted for a Waters Acquity UPLC HSS T3 column (150 mm × 2.1 mm, 1.8 μm). While these different columns all achieved effective separation of pesticide biomarkers, our choice of the Hypersil Gold AQ column was optimal for our specific set of 50 analytes across 9 chemical classes. The mobile phase composition and gradient elution program were carefully optimized to achieve effective separation of metabolites of both polar (organophosphorus) and non-polar (organochlorine) pesticides. Mobile phases are generally similar across studies, with slight variations in additives and organic solvents. A binary gradient consisting of 0.1% acetic acid in water (A) and 1:1 ratio of acetonitrile to methanol (B) was found to provide the best overall performance. Our method achieved a total run time of 18 min, comparable to other recent studies, with retention times ranging from 3.38 min for dimethyl phosphate (DMP, a dialkyl phosphate) to 15.41 min for pentachlorophenol (PCP, a fungicide), striking a balance between comprehensive analyte coverage and high-throughput analysis (Figure 4). Other recent studies have found that run times range from 9.0 min [16] to 22.0 min [12]. The optimization of the mobile phase composition gradient elution program was crucial for balancing the need for adequate separation of early-eluting polar compounds with the elution of more hydrophobic analytes later in the run. This approach allowed for effective resolution of structurally similar isomers, like the two pyrethroid metabolites trans-3-(2,2-dichlorovinyl)-2,2-dimethylcyclopropane carboxylic acid (TDCCA) and cis-3-(2,2-dibromovinyl)-2,2-dimethylcyclopropane carboxylic acid (CDCCA) [30], while minimizing matrix effects (Figure 4).

Retention times (average ± SD, n = 30) for all target analytes are presented in Table 3, with standard deviations (SD) of less than 1.0. Resolution (Rs) was calculated for structurally similar compound pairs to confirm baseline or acceptable chromatographic separation (Rs ≥ 1.5 considered baseline). Critical isomer separations - specifically CDCCA and TDCCA—achieved Rs > 1.5 under the described gradient and column conditions; representative extracted-ion chromatograms (EIC) illustrating isomer separation and co-elution checks are provided in Figure 4. Peak shape was quantified by symmetry factors (tailing factors), which were typically between 0.8 and 1.2 for most analytes; reproducibility of retention times across batches (n = 10 days) showed %RSD ≤ 1 for most compounds. These chromatographic performance assessments follow approaches described in prior multiclass urinary methods and ensure reliable discrimination of isomers and consistent peak integration for quantification [11,13,15].

### 3.3. Mass Spectrometric Detection

Mass spectrometric detection was performed using a triple quadrupole mass spectrometer by implementing a polarity switching approach in the ESI mode, allowing for the detection of both positive and negative ions within a single run. This technique is also employed by other leading researchers in the field, such as Marin-Saez et al. [13] and Zhu et al. [16] Scheduled Multiple reaction monitoring (MRM) mode was used for quantification and to ensure selectivity and specificity (Table 3). This technique optimizes sensitivity and selectivity by monitoring specific time windows for each analyte and minimizes potential interference across mass spectrometry channels when analyzing multiclass target analytes sequentially. Precursor ion *m*/*z* values ranged from 103.1 for ethylene thiourea (ETU) to 404.1 for azoxystrobin (AZO), both fungicides. Product ion *m*/*z* values spanned from 35.0 for several compounds to 372.1 for AZO. The optimization of compound-specific parameters, including precursor and product ion selection, collision energies, and cone voltages, was crucial for achieving maximum sensitivity for each target compound (Table 3).

### 3.4. Sample Preparation and Extraction

SPE has emerged as a preferred method for extracting pesticides from urine due to its efficiency and ability to handle multiple components with varying chemical properties [18]. Sample preparation employed a SPE approach using Oasis HLB 96-well plates (Figure 2). This format allows for high-throughput sample processing, which is essential for large-scale biomonitoring studies. Several SPE sorbents were evaluated, including C18, polymeric reversed-phase, and mixed-mode ion-exchange materials. The experimental setup and findings were previously discussed [18]. Oasis HLB was selected as the optimal sorbent due to its broad applicability to compounds with diverse physicochemical properties. This choice is consistent with several published methods for multiclass pesticide analysis in urine. Prior to extraction, samples underwent enzymatic deconjugation to cleave conjugated metabolites, allowing for the determination of total (free + conjugated) concentrations of organophosphorus and pyrethroid pesticides [31]. We optimized the enzyme activity and incubation conditions to ensure complete deconjugation without degrading the target analytes, as previously described [18]. Enzymatic deconjugation is employed in all methods, with similar enzyme activities (Table 1). We also confirmed that the enzymatic deconjugation step did not have any effects on the pesticides that are primarily excreted in aglycone-free forms, such as phenoxy acid herbicides [32], neonicotinoids [25,33], and dialkyl phosphates [34]. All methods, including the current one, utilize a single urine aliquot for sample preparation. The current method requires the smallest urine volume (0.2 mL) compared to others (0.25–1.0 mL), making it particularly suitable for studies with limited sample availability (Table 1).

### 3.5. Method Validation

Method validation was conducted in accordance with the guidelines set forth by the NIEHS’s Human Health Exposure Analysis Resource (HHEAR) consortium [29]. These guidelines are consistent with the bioanalytical method validation standards established by the Food and Drug Administration (FDA) [35,36].

#### 3.5.1. Linearity and Calibration

Linearity was assessed using matrix-matched calibration curves prepared in pooled urine at concentrations ranging from 0.1 to 100 ng/mL. The method showed excellent linearity across all analytes, with coefficients of determination (R^2^) values ranging from 0.988 (ETU) to 0.999 for propylene thiourea (PTU), both fungicides, indicating strong linear relationships between analyte concentration and instrument response (Table 3). All reported R^2^ values are statistically significant (*p* < 0.001), indicating strong linear relationships between analyte concentration and instrument response across the calibration range. This high degree of linearity contributes to the method’s accuracy and reliability for quantifying pesticide biomarkers across a wide concentration range. For pesticide analytes above the upper limit of linearity of 100 ng/mL, which are uncommon in the general population, urine sample dilution or a smaller aliquot (≤0.1 mL) or extending the calibration curve up to 1000 ng/mL is suggested.

Calibration curves were constructed using ordinary least squares linear regression with weighting applied to improve performance at the low end; a 1/x weighting was selected for most analytes and 1/x^2^ applied where low-concentration residuals indicated heteroscedasticity. Model selection and weighting were informed by inspection of residuals and by back-calculated concentration bias criteria across the calibration range. Residuals were examined for systematic deviation at low and high concentrations, and lack-of-fit was assessed qualitatively to ensure no curvature or concentration-dependent bias was present. Use of 1/x weighting and residual inspection is a recommended practice in multiclass LC–MS/MS biomonitoring to improve quantitation at low environmental concentrations and has been applied in comparable urinary pesticide methods [11,13,15]. Reported R^2^ values supplement these diagnostics but do not substitute for residual evaluation and weighting strategies adopted to ensure accurate quantification across the calibration range.

#### 3.5.2. Sensitivity, LOD, and LOQ

The developed method has high sensitivity, with limits of detection (LODs) ranging from 0.01 (21/50 analytes) to 0.88 ng/mL for N,N-diethyl-meta-toluamide (DEET, an insect repellent), with the majority (47/50) ≤ 0.5 ng/mL LOD. The limits of quantification (LOQs) ranged from 0.02 (14/50 analytes) to 2.92 ng/mL (DEET), with the majority (29/50) ≤ 1.0 ng/mL LOQ (Table 3). Herbicides consistently showed the lowest LODs and LOQs at 0.01 and 0.02 ng/mL, respectively. The highest LOD and LOQ were observed for DEET, an insect repellent, at 0.88 and 2.92 ng/mL, respectively. Organophosphorus insecticides showed LODs ranging from 0.01 to 0.25 ng/mL and LOQs from 0.02 to 0.84 ng/mL. These values are generally comparable to or better than those reported in recent literature. For instance, Bustamante et al. [12] reported LODs of 0.012–0.058 ng/mL, while Marin-Saez et al. [13] reported a wider range of 0.1–100 ng/mL. Gao et al. [15] report lower LOQs (0.1–16 pg/mL), but for fewer analytes. The high sensitivity of our method allows for the detection and quantification of pesticides at environmentally relevant concentrations, even at the lower end of the calibration range.

To further substantiate the reported LODs and LOQs at the low end of the calibration range, we combined instrument-based signal-to-noise (S/N) assessment with replicate low-level measurements and calibration extrapolation. LODs were defined by S/N ≥ 3 and LOQs by S/N ≥ 10, and were corroborated by replicate injections (n ≥ 10) at concentrations bracketing the LOQ for representative low-LOQ analytes (e.g., selected neonicotinoids and dialkyl phosphate biomarkers). This approach follows commonly applied practices in urinary pesticide biomonitoring where LOQs are established by the lowest concentration meeting S/N, accuracy (±20% at LOQ) and precision (RSD ≤ 20%) criteria and are subsequently confirmed in matrix by evaluating peak morphology and recovery [11]. For compounds where routine multi-batch validation was performed at 1 and 10 ng/mL, the lower LOQs reported were supported by matrix-matched calibration points, surrogate isotope-labeled internal standard compensation, and single-batch low-level assessments (extracted replicates and S/N verification) that met the LOQ acceptance criteria. Similar combined strategies for LOQ confirmation have been described for multiclass urinary pesticide methods [12,15]. These additional low-level checks demonstrate that the reported LOQs are achievable and reproducible in matrix even when routine multi-batch QC was centered at 1 and 10 ng/mL.

For analytes with LOQs higher than the 1 ng/mL routine QC level (e.g., ethylene thiourea, DEET, chlormequat), the reported LOQs reflect compound-specific matrix effects or signal suppression that prevented acceptable accuracy/precision at concentrations below those LOQs despite achieving detectable peaks at lower concentrations. This determination was based on targeted evaluation of recovery, matrix effect and precision at incremental low concentrations and on signal suppression observed in extracted urine, which is a recognized cause of elevated LOQs in multiclass urinary methods [11]. Where matrix effects or suppression were pronounced, the lowest concentration meeting predefined acceptance criteria for accuracy and precision was designated as the LOQ; this approach is consistent with previous multiclass pesticide biomonitoring methods that report higher LOQs for analytes affected by strong matrix suppression and recommend additional cleanup or alternative preparation for improvement [11,15]. For transparency, these analyte-specific LOQs are therefore reported as the lowest concentrations at which accuracy and precision acceptance criteria were met in extracted matrix, rather than the lower routine QC level.

The chromatogram displayed for DEET (Figure 4) corresponds to a solvent-based standard and therefore can exhibit a clearer peak at 1 ng/mL than an equivalent concentration in extracted urine. The LOQ reported for DEET (2.92 ng/mL) was established based on matrix-matched evaluations in extracted urine where matrix suppression led to poorer accuracy/precision at 1 ng/mL; therefore, the matrix-based LOQ is higher than the solvent standard appearance. This distinction between solvent and matrix responses is a common observation in urinary pesticide analyses and underlies the requirement to establish LOQs in matrix (not solvent) to ensure reliable quantification in real samples [11,15]. Accordingly, the LOQ reflects the lowest concentration in urine matrix satisfying S/N, recovery and precision acceptance criteria, whereas the solvent chromatogram is presented only to illustrate retention and transition selectivity.

Quantification limits below the stated calibration minimum (0.1 ng/mL) were established by combining signal-to-noise determinations, analyte-specific extracted-matrix assessments, and surrogate isotope-labeled internal standard correction to evaluate accuracy and precision at sub-calibration concentrations. For analytes with reported LOQs below 0.1 ng/mL, the instrumental response remained linear when examined at sub-0.1 ng/mL levels in matrix during targeted low-level checks, and the LOQ assignment was based on the lowest matrix concentration meeting the LOQ acceptance criteria (S/N, accuracy and precision). This pragmatic approach—using matrix-matched calibration for the primary curve while confirming sub-calibration LOQs by targeted matrix checks and IL-ISTD compensation—has precedent in urinary pesticide biomonitoring where low environmental concentrations are encountered and where matrix-matched confirmation is used to validate sub-range LOQs [12,15]. If required for reporting, extended calibration points can be included for selected analytes with sub-0.1 ng/mL LOQs in future targeted analyses.

#### 3.5.3. Accuracy and Precision

Accuracy and precision were evaluated at four levels: LLOQ (unspiked QC urine pool), low QC (1 ng/mL spike), medium QC (10 ng/mL spike), and high QC (100 ng/mL spike). Both intra-day (n = 3) and inter-day (n = 10 days) assessments were conducted. Accuracy, represented as extraction efficiency (EE%), was assessed using QC urine samples spiked at various levels, analyzed in triplicate across 10 batches (n = 30). For the purpose of discussion, we focused on the unspiked QC urine pool (LLOQ) as well as the low and mid QC spikes of 1 ng/mL and 10 ng/mL, which represent typical environmental exposure levels. EE% ranged from 85% to 107% at 1 ng/mL and 89% to 113% at 10 ng/mL, demonstrating good accuracy across the concentration range (Table 3). The lowest EE% at 1 ng/mL was observed for AZO (85%), a fungicide, while the highest was for 3-(diethylcarbamoyl)benzoic acid (DCBA, 107%, a DEET metabolite). This recovery range is among the best reported in recent literature, with other studies showing wider ranges such as 78–99% [12], 70–120% [13], and 24–119% [15]. RE% values were generally within ±15% for both concentration levels, indicating good accuracy. Precision, assessed using RSD% and CV%, was generally acceptable. At 1 ng/mL, RSD% and CV% were typically below 15%, while at 10 ng/mL, these values improved to below 10% for most analytes. Accuracy and precision data for the high QC level (100 ng/mL) were generated during method validation. At 100 ng/mL, mean relative error and relative standard deviation for the evaluated analytes met typical bioanalytical acceptance criteria (absolute RE ≤ 15% and RSD ≤ 15%) consistent with performance observed for multiresidue urinary assays reported in the literature [11,15]. These high-level QC results confirm the method’s trueness and precision across the full validated calibration range and support the upper calibration limit reported.

Validation experiments were conducted following a structured multi-day design to characterize intra- and inter-day performance. For each concentration level (low, mid, high), three replicates were analyzed per batch across ten independent batches on separate days (totaling ten days) to assess intra- and inter-day precision and accuracy, consistent with accepted biomonitoring validation practices [29]. Acceptance criteria were aligned with established biomonitoring and bioanalytical guidance: accuracy within ± 15% (±20% at the LOQ) and precision (RSD) ≤ 15% (≤20% at the LOQ), consistent with common regulatory and biomonitoring practices [15,17]. Calibration was performed using matrix-matched calibration curves (extracted blank urine) for analytes demonstrating measurable matrix effects, whereas solvent-based calibration was reserved only for analytes showing negligible matrix bias; this hybrid approach is consistent with other multi-class urinary methods to balance accuracy and practicality [18,19]. Carryover was evaluated by injecting solvent blanks after the highest calibration standard; no analyte showed carryover above 20% of the LOQ. Dilution integrity was assessed by spiking urine at concentrations above the upper calibration limit, diluting 5- and 10-fold with extracted blank urine, and confirming accuracy and precision within acceptance criteria, demonstrating reliable quantification after dilution. These validation procedures mirror practices used in recent multiclass urinary pesticide assays to ensure robust performance across batches and days [12,17].

#### 3.5.4. Matrix Effects

The use of isotope-labeled IL-ISTD for each analyte class helped compensate for potential matrix effects, as evidenced by the consistent performance across different concentration levels in QC urine samples. Quantitative assessment of matrix effects was performed using post-extraction spiking experiments to calculate matrix/solvent slope ratios and by monitoring representative post-column infusion experiments for analytes across chemical classes. For post-extraction spiking, extracted blank urine was spiked at five concentration levels and compared to equivalent standards in solvent to calculate percent signal suppression/enhancement matrix effect (%) = 100 × [(slope_matrix_/slope_solvent_) − 1)]. Representative ion suppression/enhancement values demonstrated that most analytes exhibited slope ratios within ±20% while a limited subset showed stronger suppression and were therefore quantified using matrix-matched calibration or corrected by isotope-labeled surrogates. Post-column infusion experiments confirmed zones of transient suppression correlated with early-eluting polar metabolites and major urine matrix components, but interference at target analyte retention times was <5% for native analytes and <0.5% for internal standards, indicating acceptable selectivity. Where post-column infusion showed localized suppression at retention times of co-eluting urinary components, analyte quantification was confirmed by isotope-dilution correction or matrix-matched calibration to ensure accurate results. This combined strategy (post-extraction spiking and targeted post-column infusion) follows accepted multiclass biomonitoring practice to detect and mitigate matrix effects in complex urine matrices [14,15,17].

Developing a multiclass pesticide assay is challenging due to the lack of isotopically labeled IL-ISTD for all target compounds. In cases where analyte-specific isotope-labeled internal standards were not available, representative isotopically labeled surrogates were selected based on physicochemical similarity (polarity and retention behavior), major fragmentation pathways, and their ability to track extraction recovery and ionization variability. To get around this, we chose 26 IL-ISTD that were representative of the physicochemical properties of our target analytes. We then used these as surrogate-labeled standards and thoroughly evaluated matrix effects and extraction recoveries. Based on recovery and QC data (peak-area normalization with representative IL-ISTDs), analytes quantified with matched IL-ISTDs showed RSDs typically <10% at low QC levels while analytes quantified with surrogate IL-ISTDs exhibited slightly higher variability (RSDs up to 20%). This surrogate approach has been successfully applied in other multi-class urinary pesticide methods to control bias while balancing practicality [12,15]. Validation data indicate that analytes quantified with surrogate IL-ISTDs exhibited modestly higher variability, with average additional imprecision of ~4–8% and bias up to an additional ~6% at low QC levels compared with matched-IL-ISTD analytes; these effects were mitigated by careful surrogate selection and matrix-matched calibration [16,29,37]. While labeled standards for each analyte are ideal, our results demonstrate that careful optimization and validation can still achieve effective multi-class methods, even in the absence of analyte-specific labeled IL-ISTD for some. This approach allows reliable quantification while managing costs and practicality. To further validate our method’s sensitivity and selectivity, we analyzed chromatographic peaks near the retention time of each analyte, focusing on samples at or near the lower limit of quantification. This analysis confirmed that potential interfering components were below 5% for all native analytes and below 0.5% for all internal standards, ensuring reliable detection target analytes at low concentrations, even at the lowest calibration point, and quantification across the calibration range.

#### 3.5.5. Stability

To provide empirical support for storage and handling recommendations, we evaluated stability using low- and mid-quality control spikes (1 and 10 ng/mL) with five replicate aliquots for each condition. Samples were tested after bench storage at room temperature for 24, 48, and 72 h, after three freeze-thaw cycles, and during long-term storage at −80 °C, with measurements taken at 6, 12, and 24 months. Mean recoveries across conditions remained within 80–100% of initial concentrations, and relative standard deviations were ≤20% for both quality control levels, indicating acceptable stability for typical biomonitoring workflows. Similar comprehensive stability assessments and reporting practices have been employed in recent multiclass urinary pesticide methods to substantiate claims of long-term storage stability [15,17]. The assertion of long-term stability for storage at −80 °C (3–4 years) is based on real-time data for a subset of analytes present in the G-EQUAS and OSEQAS proficiency testing program materials from previous years, for which archived aliquots were available. These aliquots showed recoveries within 70–110% of the reference values, with relative standard deviations ≤20% for the panel of representative analytes. These quantitative results support the conclusion that target analytes are stable under the tested conditions and provide essential data for longitudinal biomonitoring studies.

#### 3.5.6. Selectivity

**Figure 5:**
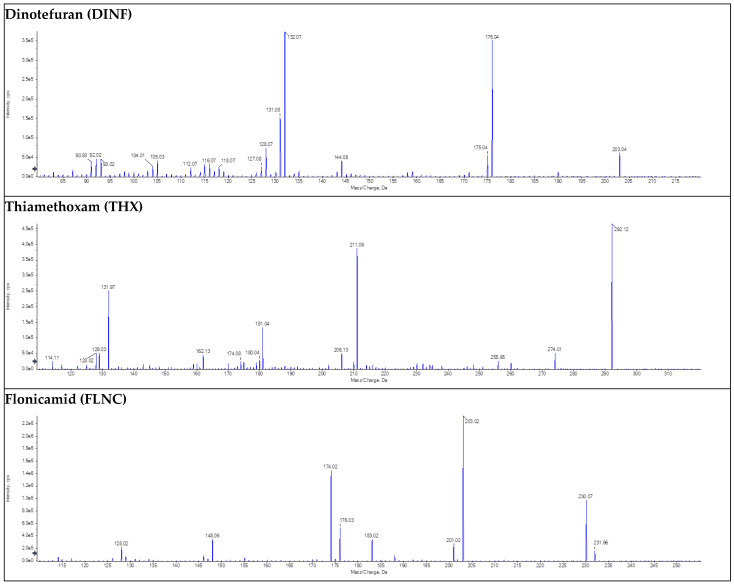

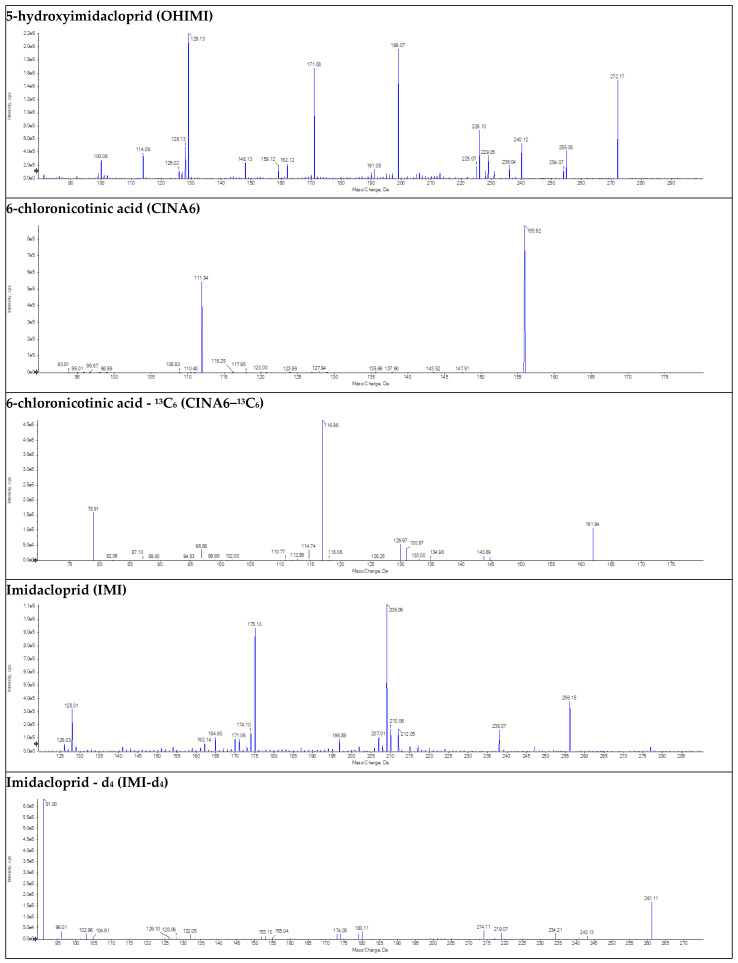

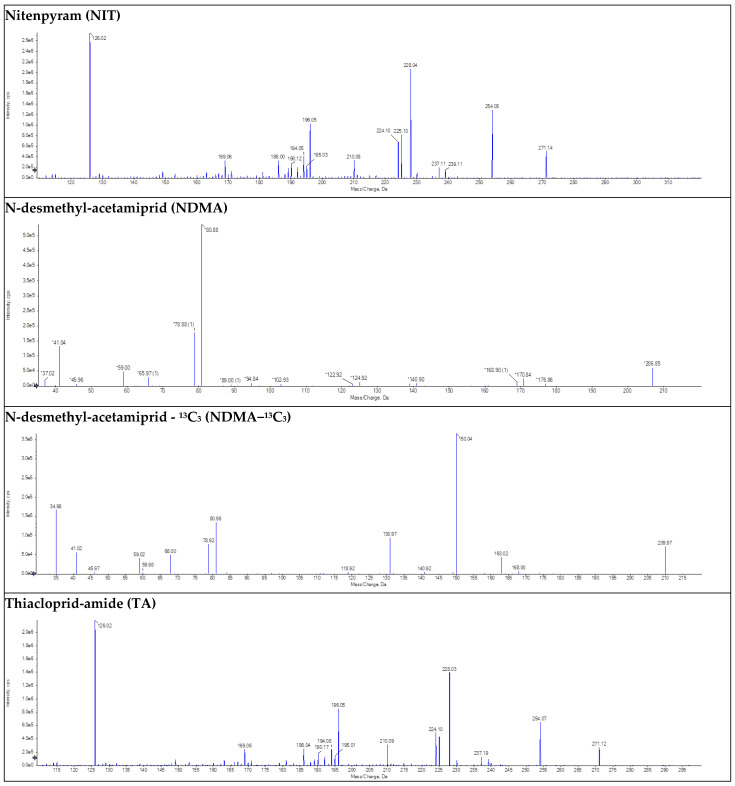

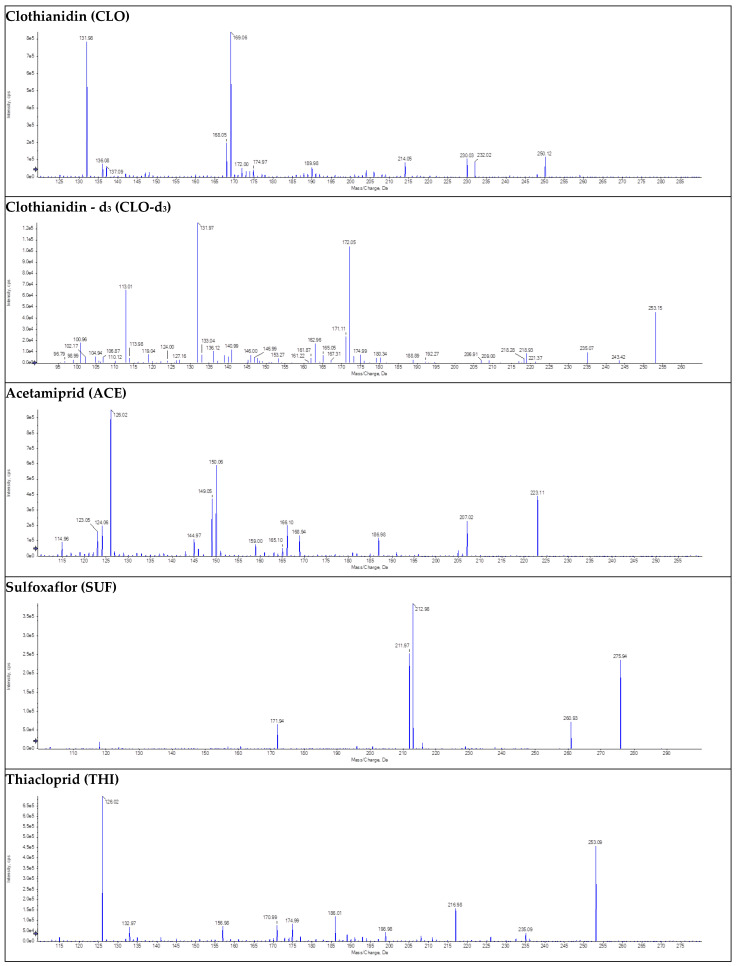
The mass spectrum of individual neonicotinoid insecticides illustrates their molecular characteristics and fragmentation patterns. Similar precursor and fragmentation ion spectra for each reference standard and IL-ISTD for all pesticide classes studied are in the Supplementary Materials.

Method selectivity was confirmed by analyzing blank urine samples from multiple sources, with no interfering peaks observed at the retention times of the target analytes. Mass spectrometric detection utilized polarity switching in electrospray ionization mode with multiple reaction monitoring, ensuring high selectivity. The mass spectrum for neonicotinoid insecticides, representing one class of pesticides, is shown in Figure 5. Additional spectra of the precursor and fragmentation ions for each reference standard and internal standard (IL-ISTD) across all pesticide classes examined are available in the Supplementary Materials (Figure S1). These data demonstrate the robustness and specificity of our method for analyzing multiple classes of pesticides in urine.

A quantitative matrix effect (ME) evaluation was performed using the post-extraction addition approach. Matrix factor (MF) values were calculated for each analyte as the peak area in post-extraction spiked urine divided by the peak area in neat solvent at equivalent concentration; the IS-normalized MF (analyte MF divided by corresponding internal standard MF) was also calculated to evaluate compensation by labeled surrogates. Experiments were conducted in five independent urine sources at two concentration levels (1 and 10 ng/mL) in triplicate (n = 3 per source). Reported MF values ranged from moderate suppression to slight enhancement for some compounds; however, IS-normalized MF values were within 0.70–1.15 for most analytes, indicating effective compensation by the selected isotopically labeled internal standards. This approach and use of surrogate IL-ISTDs to correct matrix effects are consistent with prior multi-class urinary pesticide methods [11,15,17].

#### 3.5.7. External Validation and Proficiency Testing

The robustness and reliability of our method were further demonstrated through participation in external quality assessment schemes. Specifically, 18 of the pesticide biomarkers included in our method are part of the G-EQUAS PT program (Table 2). Additionally, the OSEQAS PT program includes 11 pesticide biomarkers from our method (Table 2). This external validation provides confidence in the method’s performance and ensures comparability with other laboratories.

#### 3.5.8. Regulatory Alignment and Deviations

Method validation elements (accuracy, precision, sensitivity, selectivity, stability, and recovery) were carried out following principles consistent with recent FDA biomarker recommendations for bioanalytical methods, including evaluating accuracy across at least three concentration levels and determining intra- and inter-assay precision [29,36]. Key parameters such as accuracy (RE within ± 15% for QC levels), precision (RSD <15%), and stability testing were consistent with standard biomonitoring method practices reported in the literature. Two practical deviations from specific FDA 2025 draft elements are noted: (1) analyte-specific isotopically labeled internal standards were unavailable for all targets; representative IL-ISTDs were used as surrogates after thorough matrix effect and recovery evaluation, an approach reported in other multiclass urine methods when full isotopically labeled coverage is not feasible [15,17]; and (2) while extensive within-laboratory reproducibility was assessed, full multi-laboratory reproducibility requires ring-trial participation [29]. These deviations are explicitly reported so readers may interpret regulatory applicability in the context of resource and standard availability. Full alignment with all formal regulatory checklists will be pursued in method transfer and interlaboratory validation stages.

### 3.6. Method Strenghts

The present method enhances analyte coverage compared to recent multi-analyte urinary assays while simultaneously reducing the required sample volume and improving analytical sensitivity. For instance, Marin-Saez et al. reported 30 analytes using a 1.0 mL urine aliquot [13], whereas our assay quantifies 50 analytes with a reduced sample volume of 0.20 mL, resulting in an 8-fold increase in analytes per milliliter processed. Gao et al. identified 26 pesticide-related analytes [15]; thus, our target list represents an approximate 92% increase in analyte coverage relative to their study while maintaining similar chromatographic run times and solvent compositions. Furthermore, methods that utilize online SPE or isotope-dilution strategies have achieved LODs, but at the expense of increased instrument complexity or larger sample volumes [12,13,15,25,38]. By integrating off-line 96-well Oasis HLB SPE with UHPLC-MS/MS and representative isotopically labeled surrogates, our method achieves LODs ranging from 0.01 to 0.88 ng/mL while utilizing minimal urine volume—this is particularly advantageous for studies limited by sample availability (e.g., pediatric cohorts). Additionally, it achieves limits of detection that are comparable to, or even lower than, those reported in the literature, which typically range from 0.02 to 0.64 ng/mL for 10 pesticides in 0.30 mL of urine [25] and from 0.1 to 0.6 ng/mL for 16 pesticides in 0.2 mL of urine [38], both employing isotope-dilution online SPE-LC-MS/MS.

### 3.7. Method Limitations

While the method demonstrates favorable recovery and selectivity metrics, limitations warrant consideration. First, although surrogate isotopically labeled internal standards were used to mitigate matrix effects, targeted assessment beyond global recovery—such as class-specific matrix suppression/enhancement mapping across diverse urine pools—was not exhaustively performed; similar multiclass studies emphasize the value of expanded matrix effect characterization when sample matrices vary widely [15,17]. Second, several emerging pesticides and metabolites were excluded due to the unavailability of certified reference materials or authentic standards at the time of method development; this pragmatic constraint has been noted in other multiclass efforts and guides prioritized future expansion [12,15]. Third, the demonstrated throughput (96-well SPE format with ~18 min LC cycle) supports moderate high-throughput studies, but scaling to >1000 individual samples requires dedicated automation, instrument multiplexing, and additional QC resources to maintain data quality—limitations observed in population studies that adopt online SPE, or isotope-dilution approaches to increase throughput [12,13,15,19,25,37,38]. Finally, integration with HHEAR or other laboratory networks will require harmonized QA/QC protocols, participation in interlaboratory comparison/round-robin test/external quality assessment schemes, and alignment of calibration and reporting units, as recommended for biomonitoring harmonization [29].

### 3.8. Method Applicability and Relevance

**Figure 6:**
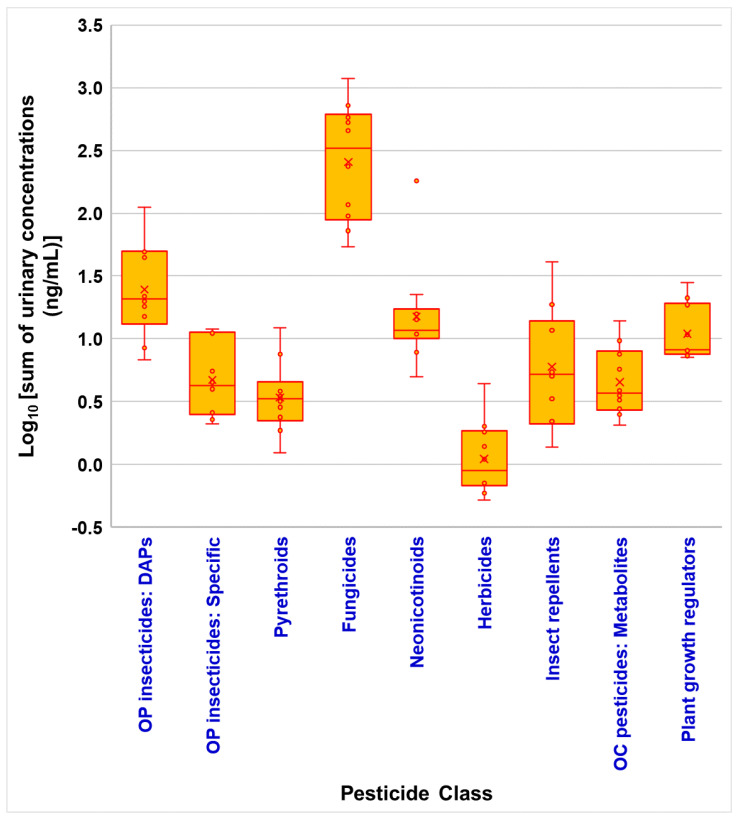
Distribution of pesticide biomarkers in urine samples from 10 adult volunteers. Box plot shows the distribution of pesticide concentrations by chemical class. The box represents the 25th and 75th percentiles, with the median indicated by the line inside the box and the mean by an X. Whiskers show the minimum and maximum values, with outliers plotted as individual points.

To demonstrate the practical utility of our developed method, we applied it to the analysis of urine samples from 10 individuals with sample ID (SID)-1 to SID-10. The method successfully quantified biomarkers from nine different pesticide classes, showcasing their capability for comprehensive pesticide exposure assessment. Figure 6 presents the arithmetic sum concentrations of pesticide compounds and their metabolites categorized by chemical class. This approach was chosen to provide a clear overview of total pesticide burden across different classes and samples, facilitating the assessment of overall exposure patterns. While molar sums would account for molecular weight differences, concentration sums offer a more direct representation of the measured values, which is particularly useful for comparing results across different studies and biomonitoring programs.

For the pilot application, analyte-specific detection frequencies and concentration ranges are reported to demonstrate method applicability despite the limited sample size. Detection frequency (percent of samples above LOD) for each analyte is presented in Table 4, and concentration ranges (min–max; median) with creatinine-unadjusted values. For key biomarkers, PBA was detected in 10/10 samples with concentrations from 0.21 to 3.64 ng/mL, TCP in 10/10 with 0.07–6.60 ng/mL, IMI in 9/10 with 0.00–0.43 ng/mL, CDCCA in 9/10 with 0.01–2.25 ng/mL, TDCCA in 4/10 with 0.00–2.20 ng/mL, and ACE in 3/10 with 0.00–0.47 ng/mL (Table 4). Where relevant, measured concentrations are compared with published biomonitoring datasets such as NHANES and contemporary multiclass urinary studies, noting that direct comparisons consider differences in sample populations, sample collection timing, and analytical LODs [1,15,23,24,26,37]. The comparison shows that observed ranges for the pilot cohort fall within or near ranges reported in population studies for several common biomarkers, supporting the method’s applicability for population biomonitoring; however, broader epidemiological inference will require larger cohorts.

**Figure 7:**
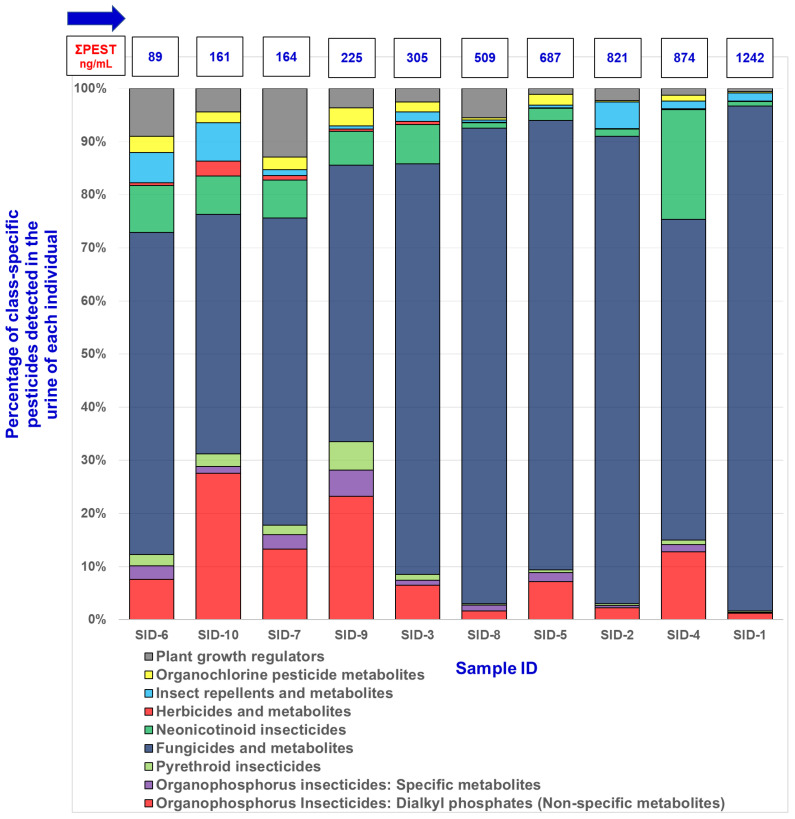
Percentage composition of pesticide biomarkers by chemical class for each individual sample represented as a stacked bar chart. Sample IDs (SID) are arranged in ascending order of total pesticide concentration (ΣPEST), from SID-6 with the lowest ΣPEST of 89 ng/mL to SID-1 with the highest ΣPEST of 1242 ng/mL. ΣPEST represents the sum of all measured pesticide biomarker concentrations in each sample. The use of arithmetic sum concentrations allows for a more direct comparison of exposure levels across different pesticide classes and individuals, which is particularly useful for biomonitoring studies and risk assessment. This approach provides a straightforward representation of the total pesticide burden, facilitating easier interpretation of exposure patterns across the study population.

Total pesticide concentrations varied widely among the samples, ranging from 89 ng/mL in SID-6 to 1242 ng/mL in SID-1, representing a nearly 14-fold difference (Figure 7). This substantial variation highlights the potential for significant inter-individual differences in pesticide exposure or metabolism. For sample extracts yielding analyte signals above the upper limit of quantification (100 ng/mL), a protocol of on-board dilution (10-fold or appropriate dilution factor) with blank followed by re-analysis was applied and concentrations were back-calculated to original sample levels; diluted analyses were repeated in duplicate to confirm results. This dilution-and-reanalysis approach follows established practice for multiresidue urinary assays when target analytes exceed the calibration range and ensures quantification remains within validated limits [11,38]. The summed group concentrations presented in Figure 6 and Figure 7 were therefore computed from validated individual analyte concentrations (either from original or diluted analyses).

Fungicides and metabolites exhibited the highest concentrations, ranging from 54 ng/mL to 1180 ng/mL, dominating the total pesticide burden. Organophosphorus insecticides, particularly dialkyl phosphates, showed a wide range of concentrations from 6.8 ng/mL to 111 ng/mL, while specific metabolites were generally lower. Herbicides and metabolites consistently showed the lowest concentrations, from 0.5 ng/mL to 4.4 ng/mL. The observed variability underscores the potential for this method to discern different exposure scenarios or metabolic profiles among individuals (Figure 7), providing valuable insights into the complex nature of pesticide exposure in human populations [23,24,26]. To provide population context, reported total-biomarker sums in our small convenience set were compared qualitatively with reference biomonitoring data. While direct summation approaches differ across studies, population surveys such as NHANES and multi-country assessments report median or percentile ranges for individual pesticide metabolites that generally lie below the upper range observed here for some individuals [1,12,15,23,24,26,37]. Comparable multiclass studies emphasize that isolated high summed concentrations in small samples may reflect episodic exposures or dietary/occupational sources and should be interpreted cautiously without larger, representative sampling [23,29]. We therefore report these observations as preliminary and recommend larger population-level comparisons, harmonized analyte selection, and unit normalization approaches (e.g., creatinine adjustment) for definitive interpretation [23,37].

## 4. Conclusions

In conclusion, a key strength of the developed method is the comprehensive coverage of pesticide classes and their metabolites, including both legacy and emerging pesticides in current use, in a single analysis. Although several prior urine methods have targeted multiple pesticide classes, the present assay expands analyte coverage and practical applicability for population studies by combining (i) simultaneous quantification of a larger set of neonicotinoid and fungicide metabolites alongside organophosphates and pyrethroids, and (ii) an SPE-based sample preparation compatible with only 0.2 mL urine, thereby enabling high-throughput analysis with small sample volumes [15,18,20]. Nevertheless, limitations remain, such as (i) comprehensive isotopically labeled internal standards are not universally available for all target analytes, which necessitated the use of representative surrogate IL-ISTDs and careful matrix-effect evaluation; and (ii) certain extreme polarity analytes may require tailored extraction or complementary techniques for improved recovery.

The current method analyzes 50 pesticide biomarkers across 9 pesticide classes, representing a significant improvement over existing literature. Its performance characteristics, including a wide linear range, low LODs and LOQs, high recoveries, and minimal matrix effects, make it a valuable tool for pesticide exposure assessment and biomonitoring studies. The method’s comprehensive coverage and alignment with established biomonitoring programs ensure its relevance for both research and regulatory applications in the field of environmental health and toxicology. Future work could focus on expanding the range of detectable compounds, particularly to include emerging pesticides and their metabolites from within this study’s chemical classes, such as adding dicamba to the herbicides class [14], expanding the neonicotinoids chemicals list [25], monitoring the USEPA’s recently banned pesticide dimethyl tetrachloroterephthalate (DCPA) [39] or its metabolite, or adding new and related classes such as fluorine-containing pesticides that are of emerging concern as “forever pesticides” [40]. In addition, using a suspect screening method like the pseudo-MRM technique [41] or a neutral loss scan [42] on a triple quadrupole mass spectrometer, as in a hybrid quantitative-qualitative assay, could be added to the targeted MRM method, allowing for the identification and semi-quantification of unexpected exposure biomarkers to new pesticides.

In summary, the validated SPE-UHPLC-MS/MS assay demonstrates robust performance for urinary biomonitoring of 50 pesticide biomarkers with limits of detection comparable to recent multiclass methods (LODs down to low-sub ng/mL), extraction efficiencies typically between 85–113%, calibration linearity across 0.1–100 ng/mL, acceptable precision (RSDs generally <15%), and demonstrable mitigation of matrix effects using representative isotopically labeled internal standards, supported by quantitative matrix factor data. Stability testing under the reported conditions indicated analyte integrity for routine handling and long-term storage at −80 °C, enabling use in large-scale studies. The method’s capability to quantify a broad range of pesticide classes in small urine volumes supports its applicability to population biomonitoring and exposure assessment, consistent with approaches used in recent multiclass urinary pesticide and exposomic methods.

## Figures and Tables

**Table 1:** Multiclass or multiresidue triple quadrupole LC/MS analytical methods that have been selected and recently published for non-persistent pesticides and metabolites in human urine after 2020.

Study #	Number of Pesticide Classes	Pesticide Class	Number of Pesticide Biomarkers in Each Class	Total Number of Pesticide Analytes	Number of Sample Preparation Steps (Number of Urine Aliquots)	Urine Aliquot Volume (mL)	Sample Preparation: Enzymatic Deconjugation	Sample Cleanup	LC Column (Particle Size, Inner Diameter, and Length)	LC Mobile Phases	LC Run Time (min.)	Mass Spectrometry	LODs/LOQs (ng/mL)	Recovery Range	Reference
1	9	Organophosphorus insecticides: Dialkyl phosphates (Non-specific metabolites)	6	50	1	0.2	ALS enzyme BGALA-RO-glucuronidase/arylsulfatase from *Helix pomatia* (β-glucuronidase activity ≈100,000 units/mL and sulfatase activity ≈47,500 units/mL)	Solid-phase extraction (SPE): Oasis HLB 96-well plate (30 mg sorbent per well, 30 µm)	Hypersil Gold AQ analytical column (3 μm, 4.6 × 150 mm) and guard column (3 µm, 4.0 × 10 mm)	A: 0.1% acetic acid in water, and B: 1:1 of acetonitrile and methanol	18.0	SCIEX 7500 Triple Quad and Qtrap-ready. ESI positive and negative mode with polarity switching.	LOD: 0.01–0.88 LOQ: 0.02–2.92	85–113%	This study
		Organophosphate insecticides: Specific metabolites	5												
		Pyrethroid insecticides	5												
		Fungicides and metabolites	10												
		Neonicotinoid insecticides	13												
		Herbicides and metabolites	3												
		Insect repellents and metabolites	4												
		Organochlorine pesticide metabolites	2												
		Plant growth regulators	2												
2	4	Organophosphate insecticides: Specific metabolites	5	23	1	1.0	β-glucuronidase type H-1 solution from *Helix pomatia* (specific activity of ~500 units/mg)	SPE with Oasis HLB 3 cm^3^	Betasil C18 column (3 μm, 2.1 × 100 mm)	A: HPLC grade water with 5% methanol and 1% CH3COOH, and B: acetonitrile	22.0	Waters XEVO-TQ-S Triple Quad MS. ESI positive and negative mode in time segments.	LOD: 0.012–0.058	78–99%	Bustamante et al., 2024, [12]
		Pyrethroid insecticides	2												
		Fungicides and metabolites	5												
		Neonicotinoid insecticides	11												
3	5	Organophosphorus Insecticides: Dialkyl phosphates (Non-specific metabolites)	8	30	1	1.0	β-Glucuronidase from *Helix pomatia* (6000 units/mL)	Salting-out liquid–liquid extraction (SALLE)	Hypersil Gold AQ column (1.9 µm, 2.1 × 100 mm)	A: Water with ammonium formate (4 mM) and 0.2% (*v*/*v*) formic acid, and B: methanol with ammonium formate (4 mM) and 0.2% (*v*/*v*) formic acid	16.0	AB SCIEX API 3200 Triple Quad MS. ESI positive and negative mode with polarity switching.	LOD: 0.01–25	70–120% (SALLE)	Marin-Saez et al., 2024, [13] *
		Organophosphate insecticides: Specific metabolites	5												
		Pyrethroid insecticides	3												
		Fungicides and metabolites	6												
		Neonicotinoid insecticides	8												
4	3	Organophosphate insecticides: Specific metabolites	5	13	1	0.25	β-Glucuronidase from *Helix Pomatia*, type H-1 (β–glucuronidase of 6300 units/mL and sulfatase of 103 units/mL)	SPE with Strata-X extraction cartridges (30 mg/3 mL)	BEH C18 column with VanGuard FIT (1.7 μm, 2.1 × 100 mm)	A: 0.035% acetic acid in water, and B: 0.035% acetic acid in methanol	17.5	AB SCIEX Tripe Quad 7500 MS. ESI positive and negative mode in time segments. with polarity switching.	LOD: 0.0038–0.091; dicamba LOD: 0.1	79–95.4%; MDA: 34.9%	Larose et al., 2023 [14]
		Pyrethroid insecticides	5												
		Herbicides and metabolites	3												
5	5	Organophosphate insecticides and metabolites	6	26	1	1.0	β-glucuronidase, from Helix pomatia. type HP-2 (β-glucuronidase activity ≥ 100,000 units/mL and sulfatase activity ≤ 7500 units/mL)	SPE with Oasis HLB cartridges (3 mL/60 mg, 30 μm)	Kinetex XB-C18 reversed-phase column (1.7 μm, 2.1 × 50 mm)	A: water with 5 mmol/L ammonium formate, and B: methanol with 5 mmol/L ammonium formate	15.0	Agilent 6495 Triple Quad MS with iFunnel technology. ESI positive and negative mode with dynamic MRM (d-MRM) mode.	LOQ: 0.1–16 pg/mL	80–119%; MDA: 24%	Gao et al., 2022 [15]
		Pyrethroid insecticides	11												
		Neonicotinoid insecticides	4												
		Fungicides and metabolites	2												
		Other pesticides (fipronil and metabolites)	3												
6	5	Organophosphate insecticides: Specific metabolites	2	31	1 One aliquot but two injections: 1st injection includes 7 pesticides. 2nd injection includes 24 pesticides.	0.5	β-glucuronidase/arylsulfatase enzyme from *Helix pomatia* (β-glucuronidase activity ≈ 100,000 units/mL; sulfatase activity ≈ 47,500 units/mL)	SPE with ABS Elut NEXUS (60 mg, 3 mL)	1st: Ultra AQ C18 column (3 μm, 2.1 × 100 mm). 2nd: Betasil C18 column (5 μm, 2.1 × 100 mm).	1st: A: 0.1% acetic acid in water, and B: 0.1% acetic acid in methanol. 2nd: A: water, and B: acetonitrile.	Two injections: 1st: 12.0 2nd: 9.0	AB SCIEX QTRAP 5500 + Triple Quad MS. ESI positive and negative mode with polarity switching.	LOD: 0.008–0.2	77–156%	Zhu et al., 2021 [16] *
		Pyrethroid insecticides	4												
		Fungicides and metabolites	7												
		Neonicotinoid insecticides	13												
		Herbicides and metabolites	4												
		Other pesticides (sulfoxaflor)	1												

* In the described analytical methodology, in addition to biomarkers of exposure to pesticides, Marin-Saez et al. (2024) [13] included mycotoxins, while Zhu et al. (2022) [16] incorporated metabolites of plasticizers, environmental phenols, and polycyclic aromatic hydrocarbons. #: Numbers.

**Table 3:** Mass spectrometric and method validation parameters.

Pesticide Class and Analyte Name	Analyte Code	Labeled Analyte Code	ESI Mode	Precursor Ion (*m*/*z*)	Product Ion (*m*/*z*)	DT (ms)	EP (V)	CE (eV)	CXP (V)	RT (min)	R^2^	LOD (ng/mL)	LOQ (ng/mL)	QC Urine Pool, Mean Conc. *, ng/mL	1 ng/mL Spike in QC Urine Pool (n = 30, 3 Replicates × 10 Batches)				10 ng/mL Spike in QC Urine Pool (n = 30, 3 Replicates × 10 Batches)			
															EE % ± SD	RE %	RSD %	CV %	EE % ± SD	RE %	RSD %	CV %
**Organophosphorus insecticides: Dialkyl phosphates (generic metabolites)**																						
Dimethylphosphate	DMP	DMP-d_6_	- ve	124.95	109.93	25	−10	−20	−21	3.38	0.999	0.05	0.15	3.07	96 ± 11	−4	4	11	94 ± 14	−6	6	15
Dimethylthiophosphate	DMTP	DMTP-d_6_	- ve	140.93	125.90	25	−10	−20	−21	3.90	0.998	0.25	0.84	2.62	101 ± 11	1	1	11	93 ± 10	−6	7	10
Dimethyldithiophosphate	DMDP	DMDP-d_6_	- ve	156.90	141.88	25	−10	−20	−21	4.29	0.993	0.03	0.12	1.14	100 ± 6	0	0	6	101 ± 5	1	1	5
Diethylphosphate	DEP	DEP-d_10_	- ve	152.98	124.95	25	−10	−20	−21	4.65	0.998	0.03	0.12	3.36	103 ± 14	6	6	14	99 ± 13	−2	2	13
Diethylthiophosphate	DETP	DETP-d_10_	- ve	168.95	140.93	25	−10	−20	−21	6.43	0.995	0.06	0.20	0.76	94 ± 10	−6	6	10	109 ± 8	9	9	8
Diethyldithiophosphate	DEDP	DEDP-d_10_	- ve	184.93	110.86	25	−10	−20	−21	6.92	0.999	0.06	0.19	0.30	102 ± 8	2	2	8	98 ± 7	−2	2	7
**Organophosphorus insecticides: Specific metabolites**																						
4-nitrophenol	PNP	PNP−^13^C_6_	- ve	137.96	107.98	25	−10	−38	−16	10.82	0.998	0.04	0.13	1.12	99 ± 9	−1	1	9	100 ± 3	0	0	3
3,5,6-trichloro-2-pyridinol	TCP	TCP−^13^C_3_	- ve	195.78	34.99	25	−10	−25	−5	13.68	0.994	0.01	0.02	0.46	105 ± 11	5	4	11	100 ± 3	0	0	3
2-[dimethoxyphosphorothioyl) sulfanyl] succinic acid	MDA	MDA-d_4_	- ve	272.90	140.93	25	−10	−14	−15	9.65	0.997	0.07	0.23	0.30	100 ± 10	−1	1	10	100 ± 5	0	0	5
2-isopropyl-4-methyl-pyrimidinol	IMPY	IMPY−^13^C_4_	+ ve	153.13	84.04	25	10	23	10	8.54	0.999	0.02	0.05	0.23	105 ± 11	5	5	11	101 ± 6	1	1	6
2-(diethylamino)-6-methylpyrimidin-4-ol	DEAMP	DEET-d_6_	+ ve	182.11	154.12	25	10	25	10	9.90	0.998	0.01	0.04	0.36	98 ± 16	0	0	16	101 ± 11	0	0	11
**Pyrethroid insecticides**																						
trans-dichlorovinyl-dimethylcyclopropane carboxylic acid	TDCCA	TDCCA−^13^C_2_	- ve	208.87	35.00	25	−10	−40	−5	13.91	0.997	0.11	0.36	2.47	103 ± 12	1	1	12	95 ± 17	−3	3	17
cis-dichlorovinyl-dimethylcyclopropane carboxylic acid	CDCCA	CDCCA−^13^C_2_	- ve	206.86	35.00	25	−10	−28	−5	14.10	0.995	0.02	0.06	1.07	102 ± 11	2	2	11	92 ± 8	−8	8	8
3-phenoxybenzoic acid	PBA	PBA−^13^C_6_	- ve	212.99	92.97	25	−10	−38	−8	13.92	0.993	0.01	0.03	2.30	102 ± 8	2	2	8	98 ± 7	−2	2	7
4-fluoro-3-phenoxybenzoic acid	FPBA	FPBA−^13^C_6_	- ve	230.80	92.97	25	−10	−38	−8	13.92	0.992	0.02	0.08	0.15	103 ± 8	3	3	8	110 ± 11	10	9	11
3-(-2-chloro-3,3,3-trifluoroprop-1-enyl)-2,2-dimethylcyclopropanecarboxylic acid	CTFCA	FPBA−^13^C_6_	- ve	240.93	204.91	25	−10	−14	−17	14.23	0.997	0.05	0.17	2.92	106 ± 12	4	4	12	97 ± 14	−2	2	14
**Fungicides and metabolites**																						
Pentachlorophenol	PCP	PCP−^13^C_6_	- ve	264.76	35.00	25	−10	−38	−16	15.41	0.999	0.04	0.13	0.72	92 ± 12	−9	9	12	99 ± 13	0	0	13
4-chlorophenol	MCP4	MCP4-d_4_	- ve	126.95	34.99	25	−10	−40	−11	12.41	0.997	0.21	0.70	1.33	93 ± 13	−8	8	13	97 ± 15	−3	3	15
Hydroxy tebuconazole	OHTBZ	IMI-d_4_	+ ve	325.18	70.02	25	10	20	10	13.83	0.993	0.01	0.03	0.52	95 ± 10	−5	5	10	98 ± 14	−2	2	14
Ethylene thiourea	ETU	CLO-d_3_	+ ve	103.00	44.05	25	10	25	10	4.55	0.988	0.80	2.66	18.79	103 ± 16	−3	3	16	95 ± 18	−6	6	18
Propylene thiourea	PTU	CLO-d_3_	+ ve	117.05	58.06	25	10	25	10	6.22	0.999	0.08	0.28	12.65	99 ± 7	−2	2	7	91 ± 9	−10	11	9
Pyrimethanil	PYRM	IMPY−^13^C_4_	+ ve	200.11	107.05	25	10	30	10	14.48	0.992	0.01	0.02	0.04	98 ± 8	−2	2	8	101 ± 8	1	1	8
Tebuconazole	TBZ	IMPY−^13^C_4_	+ ve	308.17	70.03	25	10	30	10	14.60	0.998	0.05	0.17	0.37	99 ± 14	−2	2	14	104 ± 9	4	4	9
cis-1,2,3,6-tetrahydrophthalimide	THPI	PBA−^13^C_6_	- ve	149.95	95.87	25	−10	−25	−15	8.72	0.998	0.02	0.06	134.61	104 ± 6	4	4	6	92 ± 10	−8	8	10
Azoxystrobin	AZO	ECBA-d_5_	+ ve	404.19	372.17	25	10	20	10	13.90	0.988	0.01	0.02	0.05	85 ± 16	−15	16	11	96 ± 10	−4	5	10
Pyraclostrobin	PYST	ECBA-d_5_	+ ve	388.21	194.07	25	10	20	10	14.75	0.994	0.01	0.02	0.05	89 ± 15	−11	12	15	113 ± 9	13	12	9
**Neonicotinoid insecticides**																						
6-chloronicotinic acid	CINA6	CINA6−^13^C_6_	- ve	155.92	111.94	25	−10	−14	−13	9.15	0.993	0.08	0.25	0.17	104 ± 9	4	4	9	98 ± 10	−2	2	10
Acetamiprid	ACE	CLO-d_3_	+ ve	223.11	126.02	25	10	23	10	10.12	0.993	0.01	0.02	0.06	105 ± 12	5	5	12	106 ± 7	6	6	7
N-desmethyl-acetamiprid	NDMA	NDMA−^13^C_3_	- ve	206.85	41.05	25	−10	−44	−19	9.74	0.994	0.01	0.02	0.74	96 ± 5	−4	4	5	98 ± 6	−2	2	6
Imidacloprid	IMI	IMI-d_4_	+ ve	256.15	209.06	25	10	23	12	9.70	0.995	0.01	0.02	0.60	94 ± 9	−6	6	9	105 ± 10	5	5	10
5-hydroxyimidacloprid	OHIMI	DEET-d_6_	+ ve	272.17	225.07	25	10	25	10	9.10	0.995	0.02	0.07	0.79	104 ± 14	3	3	14	97 ± 12	−3	3	12
Clothianidin	CLO	CLO-d_3_	+ ve	250.12	169.06	25	10	19	10	9.60	0.994	0.01	0.04	0.83	102 ± 8	1	1	8	96 ± 6	−3	3	6
Thiacloprid	THI	IMPY−^13^C_4_	+ ve	253.09	126.02	25	10	27	10	10.75	0.998	0.01	0.04	0.29	101 ± 7	2	2	7	103 ± 6	3	3	6
Thiacloprid-amide	TA	IMPY−^13^C_4_	+ ve	271.12	126.02	25	10	40	10	9.70	0.993	0.02	0.05	0.03	94 ± 13	−6	6	13	108 ± 4	8	8	4
Thiamethoxam	THX	IMPY−^13^C_4_	+ ve	292.12	211.06	25	10	18	10	8.96	0.994	0.02	0.06	0.88	97 ± 8	−3	3	8	89 ± 13	−11	11	13
Sulfoxaflor	SUF	PBA−^13^C_6_	- ve	275.94	212.98	25	−10	−20	−15	10.58	0.994	0.01	0.02	0.42	104 ± 9	3	3	9	98 ± 5	−2	2	5
Nitenpyram	NIT	IMI-d_4_	+ ve	271.14	126.02	25	10	40	10	9.69	0.994	0.01	0.04	0.04	88 ± 16	−12	12	16	108 ± 9	8	8	9
Flonicamid	FLNC	IMI-d_4_	+ ve	230.07	203.02	25	10	20	10	8.98	0.992	0.01	0.02	0.44	103 ± 6	3	3	6	99 ± 4	−1	1	4
Dinotefuran	DINF	IMI-d_4_	+ ve	203.04	132.07	25	10	20	10	7.49	0.995	0.02	0.08	8.81	89 ± 10	−7	7	10	103 ± 12	2	2	12
**Herbicides and metabolites**																						
2,4-dichlorophenoxyacetic acid	D24	D24−^13^C_6_	- ve	218.89	160.90	25	−10	−18	−27	12.85	0.995	0.01	0.02	0.58	100 ± 5	−1	1	5	103 ± 7	3	3	7
2,4,5-trichlorophenoxyacetic acid	T245	T245−^13^C_6_	- ve	255.00	196.86	25	−10	−20	−17	13.47	0.996	0.01	0.02	0.18	97 ± 7	−3	3	7	102 ± 8	2	2	8
Atrazine	ATZ	IMI-d_4_	+ ve	216.12	174.07	25	10	25	10	13.38	0.994	0.01	0.02	0.22	95 ± 8	−4	4	8	97 ± 15	−3	3	15
**Insect repellents and metabolites**																						
N,N-diethyl-meta-toluamide	DEET	DEET-d_6_	+ ve	192.09	119.06	25	10	23	4	13.58	0.993	0.88	2.92	0.99	103 ± 14	2	2	14	101 ± 10	1	1	10
3-(diethylcarbamoyl) benzoic acid	DCBA	DEET-d_6_	+ ve	222.12	149.05	25	10	23	4	10.55	0.996	0.01	0.02	27.35	107 ± 15	5	5	15	99 ± 11	0	0	11
3-(ethylcarbamoyl) benzoic acid	ECBA	ECBA-d_5_	+ ve	194.08	149.05	25	10	25	16	9.09	0.992	0.01	0.03	11.70	94 ± 6	−6	6	6	96 ± 8	−3	3	8
N,N-diethyl-3-(hydroxymethyl) benzamide	DHMB	DEET-d_6_	+ ve	208.12	135.05	25	10	27	4	10.28	0.997	0.01	0.02	0.15	105 ± 13	5	5	13	105 ± 11	5	5	11
**Organochlorine pesticide metabolites**																						
2,4,6-trichlorophenol	TCP246	TCP246−^13^C_6_	- ve	194.86	158.88	25	−10	−30	−11	14.41	0.993	0.08	0.25	0.37	100 ± 10	0	0	10	102 ± 15	2	2	15
2,3,5,6-tetrachlorophenol	TECP2356	TECP2356−^13^C_6_	- ve	230.78	35.00	25	−10	−50	−11	14.88	0.996	0.04	0.13	1.61	97 ± 18	−2	2	18	109 ± 17	9	9	17
**Plant growth regulators**																						
Chlormequat	CCC	CLO-d_3_	+ ve	122.09	58.07	25	10	25	10	4.91	0.992	0.52	1.73	12.89	91 ± 10	−14	15	10	92 ± 20	−1	1	20
Mepiquat	MQ	CLO-d_3_	+ ve	114.12	98.07	25	10	25	10	5.58	0.999	0.05	0.16	12.77	101 ± 7	1	1	7	96 ± 18	−3	3	18
**Labeled internal standard**																						
Dimethylphosphate - d_6_	DMP-d_6_	-	- ve	130.98	78.92	25	−10	−30	−10	3.38	-	-	-	-	-	-	-	-	-	-	-	-
Dimethylthiophosphate - d_6_	DMTP-d_6_	-	- ve	146.95	96.89	25	−10	−30	−10	3.90	-	-	-	-	-	-	-	-	-	-	-	-
Dimethyldithiophosphate - d_6_	DMDP-d_6_	-	- ve	162.92	144.89	25	−10	−30	−10	4.29	-	-	-	-	-	-	-	-	-	-	-	-
Diethylphosphate - d_10_	DEP-d_10_	-	- ve	162.93	78.92	25	−10	−30	−10	4.65	-	-	-	-	-	-	-	-	-	-	-	-
Diethylthiophosphate - d_10_	DETP-d_10_	-	- ve	179.00	94.87	25	−10	−20	−10	6.43	-	-	-	-	-	-	-	-	-	-	-	-
Diethyldithiophosphate - d_10_	DEDP-d_10_	-	- ve	194.98	110.87	25	−10	−20	−10	6.92	-	-	-	-	-	-	-	-	-	-	-	-
4-nitrophenol - ^13^C_6_	PNP−^13^C_6_	-	- ve	143.97	113.99	25	−10	−38	−16	10.82	-	-	-	-	-	-	-	-	-	-	-	-
3,5,6-trichloro-2-pyridinol - ^13^C_3_	TCP−^13^C_3_	-	- ve	199.00	34.98	25	−10	−42	−5	13.68	-	-	-	-	-	-	-	-	-	-	-	-
2-[(dimethoxyphosphorothioyl) sulfanyl] succinic acid - ^13^C_4_	MDA-d_4_	-	- ve	276.84	140.92	25	−10	−14	−15	9.65	-	-	-	-	-	-	-	-	-	-	-	-
2-isopropyl-4-methyl-pyrimidinol - ^13^C_4_	IMPY−^13^C_4_	-	+ ve	157.09	88.03	25	10	25	10	8.54	-	-	-	-	-	-	-	-	-	-	-	-
trans-dichlorovinyl-dimethylcyclopropane carboxylic acid - ^13^C_2_	TDCCA−^13^C_2_	-	- ve	209.98	35.00	25	−10	−38	−1	13.91	-	-	-	-	-	-	-	-	-	-	-	-
cis-dichlorovinyl-dimethylcyclopropane carboxylic acid - ^13^C_2_	CDCCA−^13^C_2_	-	- ve	209.98	35.00	25	−10	−32	−5	14.1	-	-	-	-	-	-	-	-	-	-	-	-
3-phenoxybenzoic acid - ^13^C_6_	PBA−^13^C_6_	-	- ve	219.02	99.00	25	−10	−38	−8	13.92	-	-	-	-	-	-	-	-	-	-	-	-
4-fluoro-3-phenoxybenzoic acid - ^13^C_6_	FPBA−^13^C_6_	-	- ve	236.80	99.00	25	−10	−38	−8	13.92	-	-	-	-	-	-	-	-	-	-	-	-
Pentachlorophenol - ^13^C_6_	PCP−^13^C_6_	-	- ve	270.70	34.90	25	−10	−56	−17	15.41	-	-	-	-	-	-	-	-	-	-	-	-
4-chlorophenol -d_4_	MCP4-d_4_	-	- ve	130.98	34.97	25	−10	−40	−5	12.41	-	-	-	-	-	-	-	-	-	-	-	-
6-chloronicotinic acid - ^13^C_6_	CINA6−^13^C_6_	-	- ve	161.94	116.96	25	−10	−16	−11	9.15	-	-	-	-	-	-	-	-	-	-	-	-
N-desmethyl-acetamiprid - ^13^C_3_	NDMA−^13^C_3_	-	- ve	209.97	41.02	25	−10	−48	−19	9.74	-	-	-	-	-	-	-	-	-	-	-	-
Imidacloprid - d_4_	IMI-d_4_	-	+ ve	261.11	214.11	25	10	25	10	9.70	-	-	-	-	-	-	-	-	-	-	-	-
Clothianidin - d_3_	CLO-d_3_	-	+ ve	253.15	172.05	25	10	19	10	9.60	-	-	-	-	-	-	-	-	-	-	-	-
2,4-dichlorophenoxyacetic acid - ^13^C_6_	D24−^13^C_6_	-	- ve	225.00	166.92	25	−10	−20	−15	12.85	-	-	-	-	-	-	-	-	-	-	-	-
2,4,5-trichlorophenoxyacetic acid - ^13^C_6_	T245−^13^C_6_	-	- ve	259.01	200.87	25	−10	−24	−27	13.47	-	-	-	-	-	-	-	-	-	-	-	-
N,N-diethyl-meta-toluamide - d_6_	DEET-d_6_	-	+ ve	198.16	119.04	25	10	25	4	13.58	-	-	-	-	-	-	-	-	-	-	-	-
3-(ethylcarbamoyl) benzoic acid - d_5_	ECBA-d_5_	-	+ ve	199.11	149.03	25	10	25	10	9.09	-	-	-	-	-	-	-	-	-	-	-	-
2,4,6-trichlorophenol - ^13^C_6_	TCP246−^13^C_6_	-	- ve	201.01	34.99	25	−10	−50	−5	14.41	-	-	-	-	-	-	-	-	-	-	-	-
2,4,5,6-tetrachlorophenol - ^13^C_6_	TECP2356−^13^C_6_	-	- ve	236.78	35.00	25	−10	−50	−11	14.88	-	-	-	-	-	-	-	-	-	-	-	-

* Abbreviations: DT = dwell time (ms), EP = entrance potential (V), CE = collision energy (V), CXP = collision cell exit potential (V).

**Table 4:** Urinary concentrations (median, minimum, maximum; ng/mL) measured in this study (n = 10), and comparable NHANES summary data for the 50 pesticide biomarkers across nine chemical classes.

Pesticide Class and Analyte Name	Analyte Code	Conc. Unit	This Study (n = 10)					NHANES (Creatinine Unadjusted)			
			LOD (ng/mL)	% Detection (% n > LOD)	Median (50th Percentile)	Min.	Max.	Survey Year	Sample Size	LOD (µg/L or ng/mL)	50th Percentile (95% CI)
**Organophosphorus insecticides: Dialkyl phosphates (generic metabolites)**											
Dimethylphosphate	DMP	ng/mL	0.05	100	9.02	0.53	31.20	2017–2018	2801	0.1	1.30 (1.10–1.49)
Dimethylthiophosphate	DMTP	ng/mL	0.25	100	2.86	1.53	7.79	2017–2018	2800	0.1	0.639 (0.565–0.743)
Dimethyldithiophosphate	DMDP	ng/mL	0.03	100	1.08	0.37	5.79	2017–2018	2804	0.1	0.105 (<LOD–0.120)
Diethylphosphate	DEP	ng/mL	0.03	100	6.84	2.28	71.58	2017–2018	2804	0.1	2.20 (1.96–2.41)
Diethylthiophosphate	DETP	ng/mL	0.06	100	0.46	0.14	1.76	2017–2018	2796	0.1	0.120 (<LOD-0.142)
Diethyldithiophosphate	DEDP	ng/mL	0.06	100	0.25	0.19	0.36	2017–2018	2804	0.1	<LOD
**Organophosphorus insecticides: Specific metabolites**											
4-nitrophenol	PNP	ng/mL	0.04	100	0.99	0.44	5.89	2015–2016	3039	0.1	0.590 (0.540–0.640)
3,5,6-trichloro-2-pyridinol	TCP	ng/mL	0.01	100	1.28	0.07	6.60	2015–2016	3033	0.1	1.20 (1.10–1.30)
2-[dimethoxyphosphorothioyl) sulfanyl] succinic acid	MDA	ng/mL	0.07	100	0.54	0.24	3.83	2015–2016	3028	0.5	<LOD
2-isopropyl-4-methyl-pyrimidinol	IMPY	ng/mL	0.02	100	0.26	0.19	1.47	2015–2016	3038	0.1	<LOD
2-(diethylamino)-6-methylpyrimidin-4-ol	DEAMP	ng/mL	0.01	100	0.21	0.07	5.87	-	-	-	-
**Pyrethroid insecticides**											
trans-dichlorovinyl-dimethylcyclopropane carboxylic acid	TDCCA	ng/mL	0.11	40	0.05	0.00	2.20	2015–2016	3022	0.6	<LOD
cis-dichlorovinyl-dimethylcyclopropane carboxylic acid	CDCCA	ng/mL	0.02	90	0.26	0.01	2.25	-	-	-	-
3-phenoxybenzoic acid	PBA	ng/mL	0.01	100	0.88	0.21	3.64	2015–2016	3022	0.1	0.680 (0.610–0.740)
4-fluoro-3-phenoxybenzoic acid	FPBA	ng/mL	0.02	100	0.09	0.04	1.28	2015–2016	3036	0.1	<LOD
3-(-2-chloro-3,3,3-trifluoroprop-1-enyl)-2,2- dimethylcyclopropanecarboxylic acid	CTFCA	ng/mL	0.05	100	1.63	0.95	3.10	-	-	-	-
**Fungicides and metabolites**											
Pentachlorophenol	PCP	ng/mL	0.04	80	0.42	0.00	1.64	2003–2004	2354	0.5	<LOD
4-chlorophenol	MCP4	ng/mL	0.21	100	1.90	0.76	3.56	-	-	-	-
Hydroxy tebuconazole	OHTBZ	ng/mL	0.01	100	0.27	0.09	8.76	-	-	-	-
Ethylene thiourea	ETU	ng/mL	0.80	100	10.25	3.58	13.58	2007–2008	2571	0.21	<LOD
Propylene thiourea	PTU	ng/mL	0.08	90	19.18	0.00	23.72	2007–2008	2573	0.36	<LOD
Pyrimethanil	PYRM	ng/mL	0.01	100	0.08	0.05	0.14	-	-	-	-
Tebuconazole	TBZ	ng/mL	0.05	100	0.31	0.18	0.51	-	-	-	-
cis-1,2,3,6-tetrahydrophthalimide	THPI	ng/mL	0.02	100	317.33	39.55	1149.70	-	-	-	-
Azoxystrobin	AZO	ng/mL	0.01	0	0.00	0.00	0.00	-	-	-	-
Pyraclostrobin	PYST	ng/mL	0.01	90	0.03	0.01	0.08	-	-	-	-
**Neonicotinoid insecticides**											
6-chloronicotinic acid	CINA6	ng/mL	0.08	100	0.27	0.14	1.61	-	-	-	-
Acetamiprid	ACE	ng/mL	0.01	30	0.00	0.00	0.47	2015–2016	2427	0.3	<LOD
N-desmethyl-acetamiprid	NDMA	ng/mL	0.01	100	0.46	0.09	3.96	2015–2016	3012	0.2	<LOD
Imidacloprid	IMI	ng/mL	0.01	90	0.16	0.00	0.43	2015–2016	2405	0.4	<LOD
5-hydroxyimidacloprid	OHIMI	ng/mL	0.02	100	0.85	0.31	1.85	2015–2016	2312	0.4	<LOD
Clothianidin	CLO	ng/mL	0.01	100	0.50	0.10	6.23	2015–2016	2423	0.2	<LOD
Thiacloprid	THI	ng/mL	0.01	100	0.25	0.22	0.34	2015–2016	2408	0.03	<LOD
Thiacloprid-amide	TA	ng/mL	0.02	10	0.00	0.00	0.27	-	-	-	-
Thiamethoxam	THX	ng/mL	0.02	100	0.84	0.57	1.33	-	-	-	-
Sulfoxaflor	SUF	ng/mL	0.01	100	0.41	0.27	1.30	-	-	-	-
Nitenpyram	NIT	ng/mL	0.01	0	0.00	0.00	0.00	-	-	-	-
Flonicamid	FLNC	ng/mL	0.01	100	0.29	0.21	0.43	-	-	-	-
Dinotefuran	DINF	ng/mL	0.02	100	6.25	2.60	169.40	-	-	-	-
**Herbicides and metabolites**											
2,4-dichlorophenoxyacetic acid	D24	ng/mL	0.01	100	0.61	0.30	4.07	2015–2016	3034	0.15	0.330 (0.290–0.360)
2,4,5-trichlorophenoxyacetic acid	T245	ng/mL	0.01	80	0.15	0.00	0.50	2009–2010	2747	0.1	<LOD
Atrazine	ATZ	ng/mL	0.01	80	0.12	0.00	0.80	2007–2008	2588	0.5	<LOD
**Insect repellents and metabolites**											
N,N-diethyl-meta-toluamide	DEET	ng/mL	0.88	60	1.00	0.45	2.58	2013–2014	2667	0.083	<LOD
3-(diethylcarbamoyl) benzoic acid	DCBA	ng/mL	0.01	100	1.61	0.10	21.22	2015–2016	2989	0.2	2.89 (1.94–4.31)
3-(ethylcarbamoyl) benzoic acid	ECBA	ng/mL	0.01	100	2.08	0.10	17.68	2015–2016	2386	0.2	1.02 (0.652–1.47)
N,N-diethyl-3-(hydroxymethyl) benzamide	DHMB	ng/mL	0.01	70	0.05	0.01	0.85	2013–2014	2659	0.089	<LOD
**Organochlorine pesticide metabolites**											
2,4,6-trichlorophenol	TCP246	ng/mL	0.08	90	0.56	0.00	1.45	2009–2010	2749	0.5	<LOD
2,3,5,6-tetrachlorophenol	TECP2356	ng/mL	0.04	100	3.06	1.93	13.15	-	-	-	-
**Plant growth regulators**											
Chlormequat	CCC	ng/mL	0.52	100	4.27	3.47	23.50	-	-	-	-
Mepiquat	MQ	ng/mL	0.05	100	3.68	3.60	8.10	-	-	-	-

## Data Availability

The original contributions presented in this study are included in the article/Supplementary Materials. Further inquiries can be directed to the corresponding author.

## Supplementary Materials

The following supporting information can be downloaded at: https://www.mdpi.com/article/10.3390/jox16020067/s1. Table S1. Pesticide chemical class, native and isotope-labelled analyte name, analyte code, CAS number, vendor, and catalog number for studied pesticides and metabolites. Figure S1: Mass spectra for the study analytes across all pesticide classes.

## Abbreviations

The following abbreviations are used in this manuscript:

ACE	Acetamiprid
ATZ	Atrazine
AZO	Azoxystrobin
CCC	Chlormequat
CDCCA	cis-dichlorovinyl-dimethylcyclopropane carboxylic acid
CHEAR	Children’s Health Exposure Analysis Resource
CINA6	6-chloronicotinic acid
CLO	Clothianidin
CTFCA	3-(-2-chloro-3,3,3-trifluoroprop-1-enyl)-2,2- dimethylcyclopropanecarboxylic acid
CTQ-OSEQAS	Canadian External Quality Assessment Scheme for Organic Substances in Urine
CV	Coefficient of variation
D24	2,4-dichlorophenoxyacetic acid
DCBA	3-(diethylcarbamoyl) benzoic acid
DEAMP	2-(diethylamino)-6-methylpyrimidin-4-ol
DEDP	Diethyldithiophosphate
DEET	N,N-diethyl-meta-toluamide
DEP	Diethylphosphate
DETP	Diethylthiophosphate
DHMB	N,N-diethyl-3-(hydroxymethyl) benzamide
DINF	Dinotefuran
DMDP	Dimethyldithiophosphate
DMP	Dimethylphosphate
DMTP	Dimethylthiophosphate
ECBA	3-(ethylcarbamoyl) benzoic acid
ETU	Ethylene thiourea
FLNC	Flonicamid
FPBA	4-fluoro-3-phenoxybenzoic acid
G-EQUAS	German External Quality Assessment Scheme for analyses of biological materials
HBM	Human biomonitoring
HHEAR	Human Health Exposure Analysis Resource
IL-ISTD	Isotope-labeled internal standards
IMI	Imidacloprid
IMPY	2-isopropyl-4-methyl-pyrimidinol
LLOQ	Lower limit of quantification
LOD	Limit of detection
LOQ	Limit of quantification
MCP4	4-chlorophenol
MDA	2-[dimethoxyphosphorothioyl) sulfanyl] succinic acid
MQ	Mepiquat
MRM	Multiple reaction monitoring
NDMA	N-desmethyl-acetamiprid
NHANES	National Health and Nutrition Examination Survey
NIT	Nitenpyram
OHIMI	5-hydroxyimidacloprid
OHTBZ	Hydroxy tebuconazole
PBA	3-phenoxybenzoic acid
PCP	Pentachlorophenol
PHI	Protected health information
PII	Personally identifiable information
PNP	4-nitrophenol
PT	Proficiency testing
PTU	Propylene thiourea
PYRM	Pyrimethanil
PYST	Pyraclostrobin
QC	Quality control
RE	Relative error
SPE	Solid-phase extraction
SUF	Sulfoxaflor
T245	2,4,5-trichlorophenoxyacetic acid
TA	Thiacloprid-amide
TBZ	Tebuconazole
TCP	3,5,6-trichloro-2-pyridinol
TCP246	2,4,6-trichlorophenol
TDCCA	trans-dichlorovinyl-dimethylcyclopropane carboxylic acid
TECP2356	2,3,5,6-tetrachlorophenol
THI	Thiacloprid
THPI	cis-1,2,3,6-tetrahydrophthalimide
THX	Thiamethoxam
UHPLC-MS/MS	Ultra-high-performance liquid chromatography-tandem mass spectrometry
