# Supplementary material for: SPE–UHPLC–MS/MS Method for Simultaneous Quantification of 50 Pesticide Biomarkers Across Nine Current-Use Chemical Classes in Human Urine
Source: J Xenobiot. 2026 Apr 13;16(2):67. doi: 10.3390/jox16020067 (PMC13117499; doi:10.3390/jox16020067)
Supplement: Supplementary file 1 [file jox-16-00067-s001.zip › jox-4198186-supplementary.pdf]

Article

Supplementary Material

SPE-UHPLC-MS/MS Method for Simultaneous Quantification of 50 Pesticide Biomarkers Across Nine Current-Use Chemical Classes in Human Urine

Ravikumar Jagani, Jasmin Chovatiya, Hiraj Patel, Sandipkumar Teraiya, Divya Pulivarthi, and Syam S. Andra *

Institute for Exposomic Research, Department of Environmental Medicine, Icahn School of Medicine at Mount Sinai, New York, NY 10029, USA

* Correspondence: syam.andra@mssm.edu

Table S1. Pesticide chemical class, native and isotope-labelled analyte name, analyte code, CAS number, vendor, and catalog number for studied pesticides and metabolites.

#	Pesticide Chemical Class	Analyte #	Full Analyte Name	Analyte Code	CAS#	Vendor*	Vendor catalog #
1	Organophosphorus insecticides: Dialkyl phosphates (generic metabolites)	1	Dimethylphosphate	DMP	813-78-5	CIL	ES-5548-10X (Dialkyl Phosphate and Phosphorothioate Cocktail (unlabeled)).
		2	Dimethylthiophosphate	DMTP	59401-04-6	CIL	
		3	Dimethyldithiophosphate	DMDP	756-80-9	CIL	
		4	Diethylphosphate	DEP	598-02-7	CIL	
		5	Diethylthiophosphate	DETP	5871-17-0	CIL	
		6	Diethyldithiophosphate	DEDP	298-06-6	CIL	
2	Organophosphorus insecticides: Specific metabolites	7	4-nitrophenol	PNP	100-02-7	CIL	ULM-8892-1.2
		8	3,5,6-trichloro-2-pyridinol	TCP	6515-38-4	CIL	ULM-9204-1.2
		9	2-[dimethoxyphosphorothioyl) sulfanyl] succinic acid	MDA	1190-28-9	CIL	ULM-9073-MT-1.2
		10	2-isopropyl-4-methyl-pyrimidinol	IMPY	2814-20-2	CIL	ULM-7432-1.2
		11	2-(diethylamino)-6-methylpyrimidin-4-ol	DEAMP	42487-72-9	SA	34546
3	Pyrethroid insecticides	12	trans-dichlorovinyl-dimethylcyclopropane carboxylic acid	TDCCA	59042-50-1	CIL	ULM-9175-1.2
		13	cis-dichlorovinyl-dimethylcyclopropane carboxylic acid	CDCCA	59042-49-8	CIL	ULM-9176-1.2
		14	3-phenoxybenzoic acid	PBA	3739-38-6	CIL	ULM-6781-SA-1.2
		15	4-fluoro-3-phenoxybenzoic acid	FPBA	77279-89-1	CIL	ULM-7391-1.2
		16	3-(-2-chloro-3,3,3-trifluoroprop-1-enyl)-2,2- dimethylcyclopropanecarboxylic acid	CTFCA	72748-35-7	AAB	AA00IC8C
4	Fungicides and metabolites	17	Pentachlorophenol	PCP	87-86-5	CIL	ULM-7600-1.2
		18	4-chlorophenol	MCP4	106-48-9	CIL	ULM-2430-0.1
		19	Hydroxy tebuconazole	OHTBZ	212267-64-6	SA	72843
		20	Ethylene thiourea	ETU	96-45-7	SA	45531
		21	Propylene thiourea	PTU	2122-19-2	SA	32949
		22	Pyrimethanil	PYRM	53112–28–0	TRC	P997250
		23	Tebuconazole	TBZ	107534-96-3	SA	32013
		24	cis-1,2,3,6-tetrahydrophthalimide	THPI	1469-48-3	SA	T14206
		25	Azoxystrobin	AZO	131860-33-8	SA	31697
		26	Pyraclostrobin	PYST	175013-18-0	SA	33696
5	Neonicotinoid insecticides	27	6-chloronicotinic acid	CINA6	5326-23-8	CIL	ULM-9604-1.2
		28	Acetamiprid	ACE	135410-20-7	SA	33674
		29	N-desmethyl-acetamiprid	NDMA	190604-92-3	CIL	ULM-10863-S
		30	Imidacloprid	IMI	138261-41-3	SA	37894
		31	5-hydroxyimidacloprid	OHIMI	380912-09-4	CIL	ULM-11068-1.2
		32	Clothianidin	CLO	210880-92-5	SA	33589
		33	Thiacloprid	THI	111988-49-9	SA	37905
		34	Thiacloprid-amide	TA	676228–91–4	CIL	ULM-10869-S
		35	Thiamethoxam	THX	153719–23–4	SA	37924
		36	Sulfoxaflor	SUF	946578–00–3	CIL	ULM-9870-S
		37	Nitenpyram	NIT	150824-47-8	SA	46077
		38	Flonicamid	FLNC	158062-67-0	SA	32509
		39	Dinotefuran	DINF	165252-70-0	CIL	ULM-9732-S
6	Herbicides and metabolites	40	2,4-dichlorophenoxyacetic acid	D24	94-75-7	SA	35826
		41	2,4,5-trichlorophenoxyacetic acid	T245	93-76-5	CIL	ULM-7213-1.2
		42	Atrazine	ATZ	1912–24–9	SA	45330

#	Pesticide Chemical Class	Analyte #	Full Analyte Name	Analyte Code	CAS#	Vendor*	Vendor catalog #
7	Insect repellents and metabolites	43	N,N-diethyl-meta-toluamide	DEET	134-62-3	CIL	ULM-7600-1.2
		44	3-(diethylcarbamoyl) benzoic acid	DCBA	72236-23-8	AAB	AA005J12
		45	3-(ethylcarbamoyl) benzoic acid	ECBA	126926-33-8	E	EN300-1265793
		46	N,N-diethyl-3-(hydroxymethyl) benzamide	DHMB	72236-22-7	TRC	H934200
8	Organochlorine pesticide metabolites	47	2,4,6-trichlorophenol	TCP246	88-06-2	CIL	ULM-7418-1.2
		48	2,3,5,6-tetrachlorophenol	TECP2356	935-95-5	CIL	ULM-7213-1.2
9	Plant growth regulators	49	Chlormequat	CCC	7003-89-6	SA	45387
		50	Mepiquat	MQ	15302-91-7	SA	36151
		A	Dimethylphosphate - d_6	DMP-d_6		CIL	ES-5547-10X (Dialkyl Phosphate and Phosphorothioate Cocktail (D, 98%))
		B	Dimethylthiophosphate - d_6	DMTP-d_6		CIL	
		C	Dimethyldithiophosphate - d_6	DMDP-d_6		CIL	
		D	Diethylphosphate - d_{10}	DEP-d_{10}		CIL	
		E	Diethylthiophosphate - d_{10}	DETP-d_{10}		CIL	
		F	Diethyldithiophosphate - d_{10}	DEDP-d_{10}		CIL	
		G	4-nitrophenol - $^{13}C_6$	PNP-$^{13}C_6$		CIL	CLM-789-1.2
		H	3,5,6-trichloro-2-pyridinol - $^{13}C_3$	TCP-$^{13}C_3$		CIL	CLM-9049-1.2
		I	2-[(dimethoxyphosphorothioyl) sulfanyl] succinic acid - $^{13}C_4$	MDA-d_4		CIL	CLM-9050-MT-1.2
		J	2-isopropyl-4-methyl-pyrimidinol - $^{13}C_4$	IMPY-$^{13}C_4$		CIL	CLM-4538-1.2
		K	trans-dichlorovinyl-dimethylcyclopropane carboxylic acid - $^{13}C_2$	TDCCA-$^{13}C_2$		CIL	CDLM-9206-1.2
		L	cis-dichlorovinyl-dimethylcyclopropane carboxylic acid - $^{13}C_2$	CDCCA-$^{13}C_2$		CIL	CDLM-9205-1.2
		M	3-phenoxybenzoic acid - $^{13}C_6$	PBA-$^{13}C_6$		CIL	CLM-4542-SA-1.2
		N	4-fluoro-3-phenoxybenzoic acid - $^{13}C_6$	FPBA-$^{13}C_6$		CIL	CLM-7389-1.2
		O	Pentachlorophenol - $^{13}C_6$	PCP-$^{13}C_6$		CIL	ULM-6894-1.2
		P	4-chlorophenol -d_4	MCP4-d_4		TRC	C375042
		Q	6-chloronicotinic acid - $^{13}C_6$	CINA6-$^{13}C_6$		CIL	CLM-9598-1.2
		R	N-desmethyl-acetamiprid - $^{13}C_3$	NDMA-$^{13}C_3$		CIL	CNLM-10862-S
		S	Imidacloprid - d_4	IMI-d_4		SA	34170
		T	Clothianidin – d_3	CLO-d_3		SA	56816-50mg
		U	2,4-dichlorophenoxyacetic acid - $^{13}C_6$	D24-$^{13}C_6$		CIL	CLM-1858-1.2
		V	2,4,5-trichlorophenoxyacetic acid - $^{13}C_6$	T245-$^{13}C_6$		CIL	CLM-4551-1.2
		W	N,N-diethyl-meta-toluamide – d_6	DEET-d_6		CIL	DLM-4762-1.2
		X	3-(ethylcarbamoyl) benzoic acid – d_5	ECBA-d_5		TRC	E899677
		Y	2,4,6-trichlorophenol - $^{13}C_6$	TCP246-$^{13}C_6$		CIL	CLM-1804-1.2
		Z	2,4,5,6-tetrachlorophenol - $^{13}C_6$	TECP2356-$^{13}C_6$		TRC	T291382

Vendor*: AAB (AA Blocks), E (Enamine), CIL (Cambridge Isotope Laboratories), SA (Sigma-Aldrich), and TRC (Toronto Research Chemicals)

Figure S1. Mass spectra for the study analytes across all pesticide classes.

Organophosphorus insecticides: Dialkyl phosphates

1. DMP

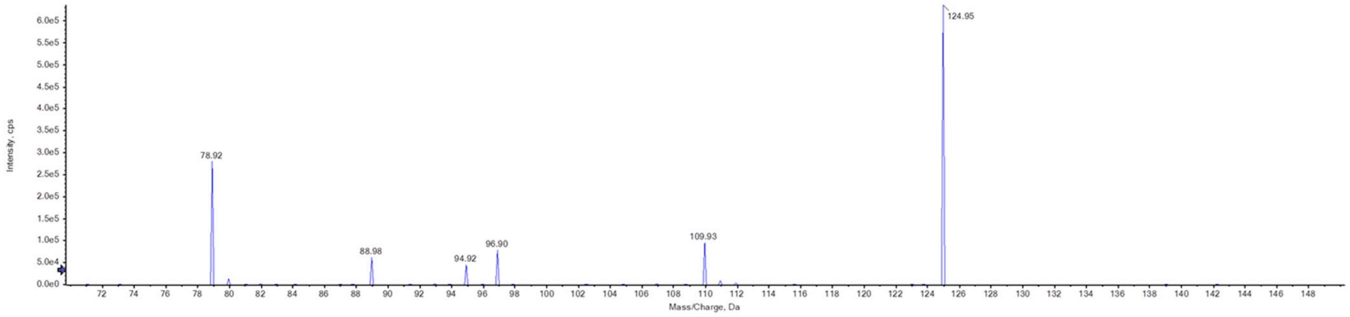

DMP-d_6

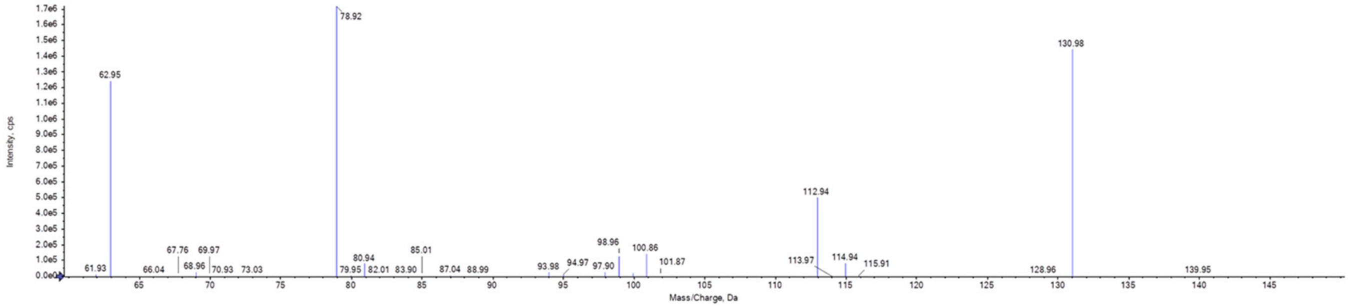

2. DMTP

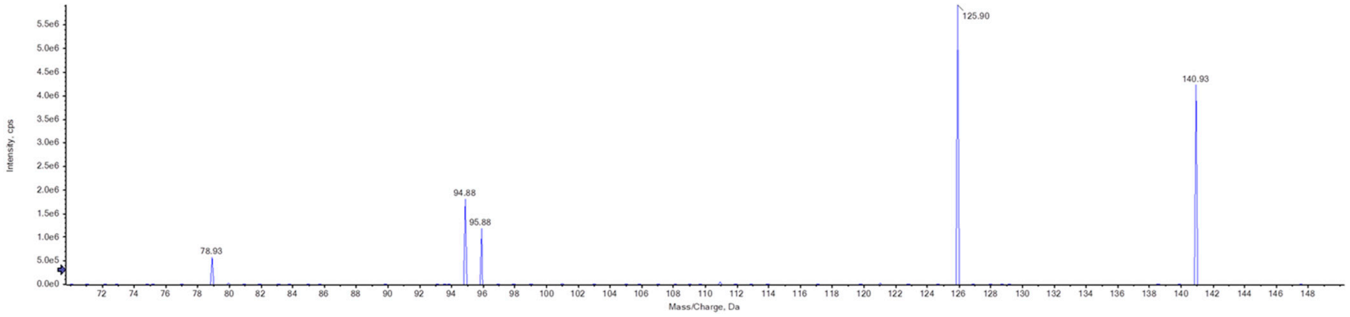

DMTP-d_6

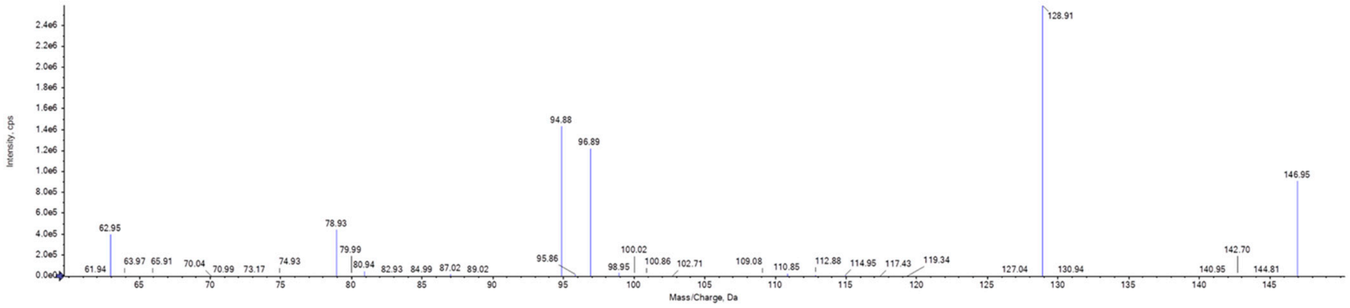

3. DMDP

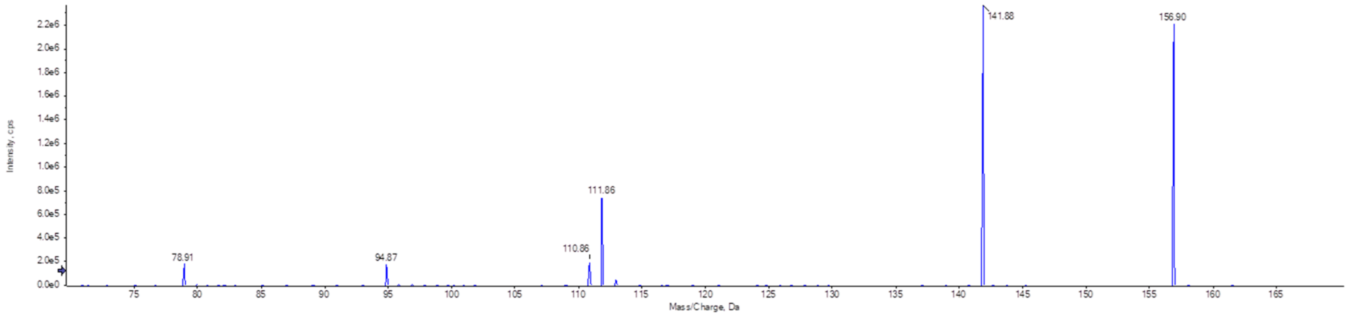

DMDP-d_6

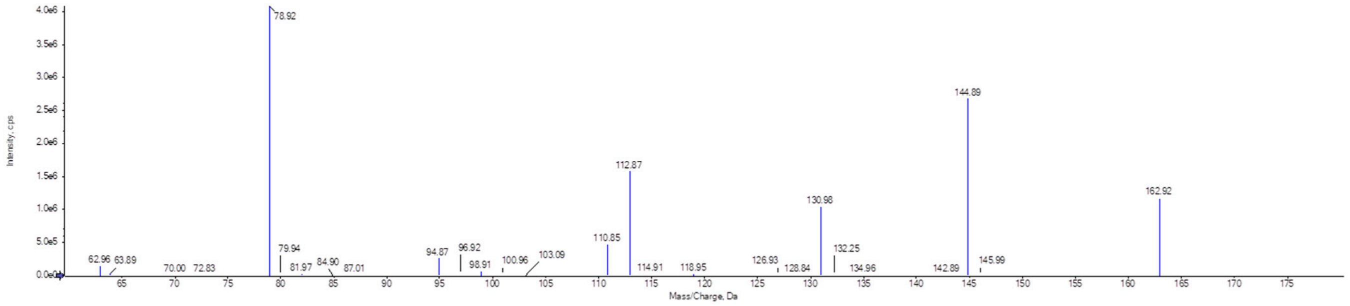

4. **DEP**

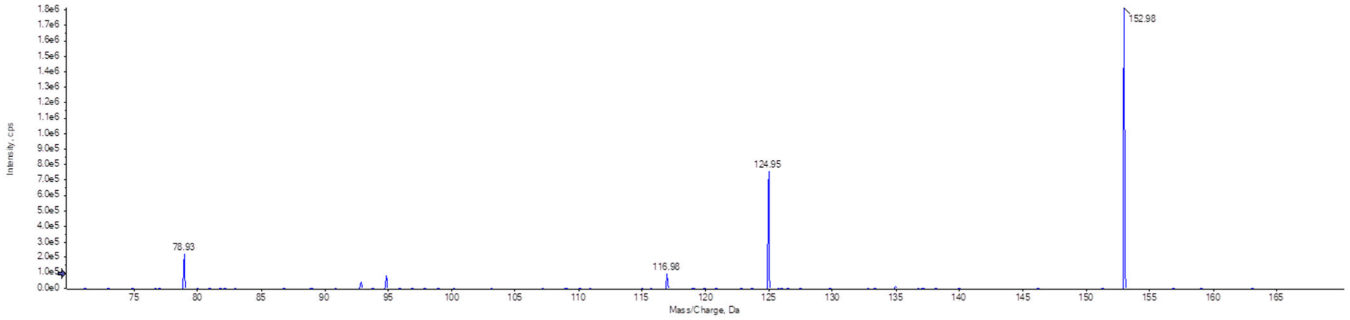

DEP-d_{10}

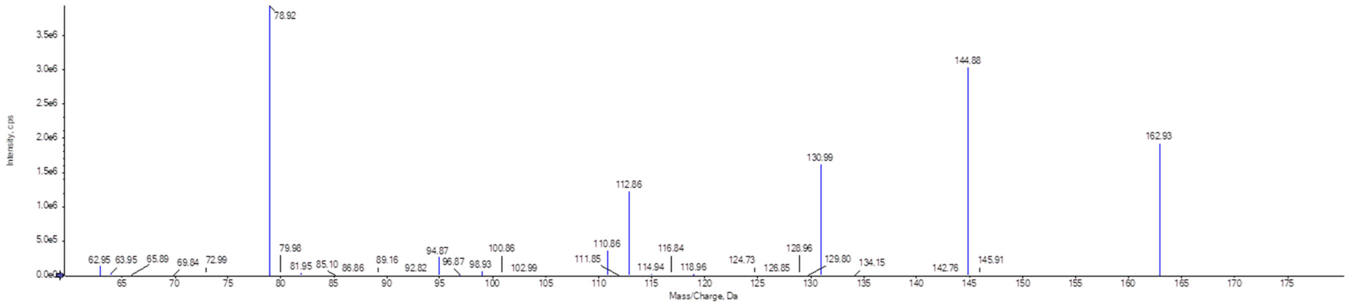

5. **DETP**

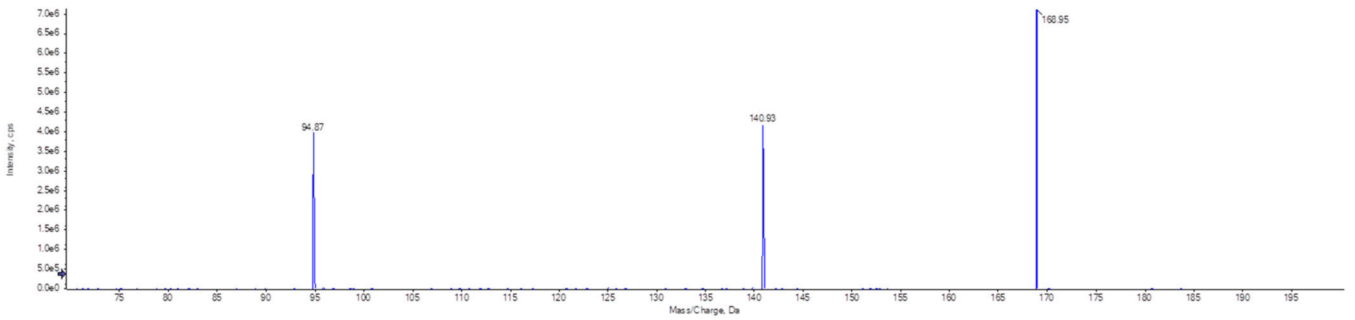

DETP-d_{10}

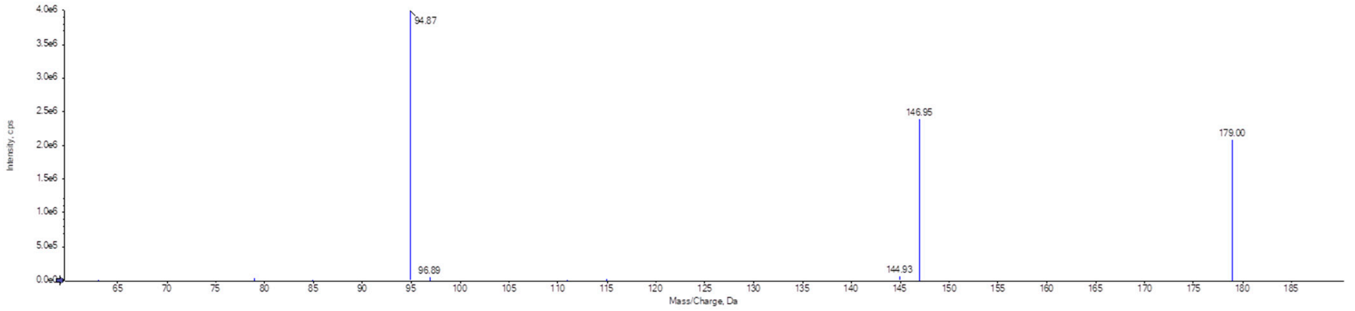

6. **DEDP**

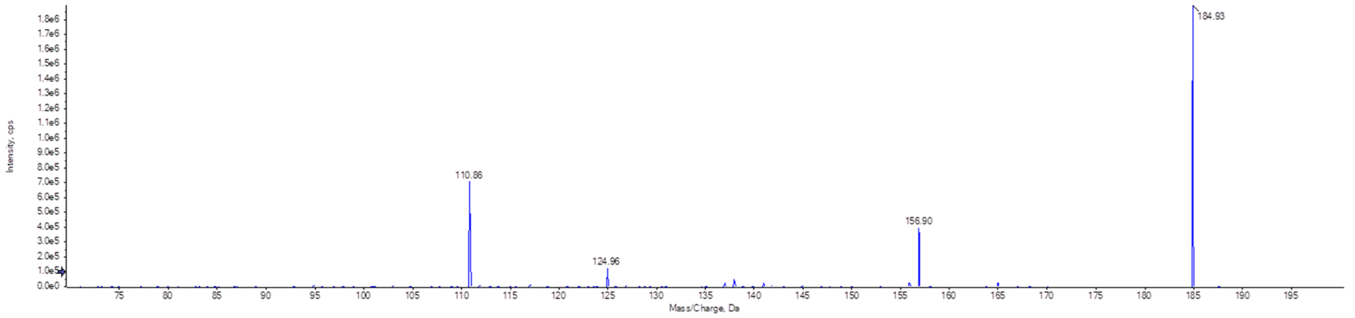

DEDP-d_{10}

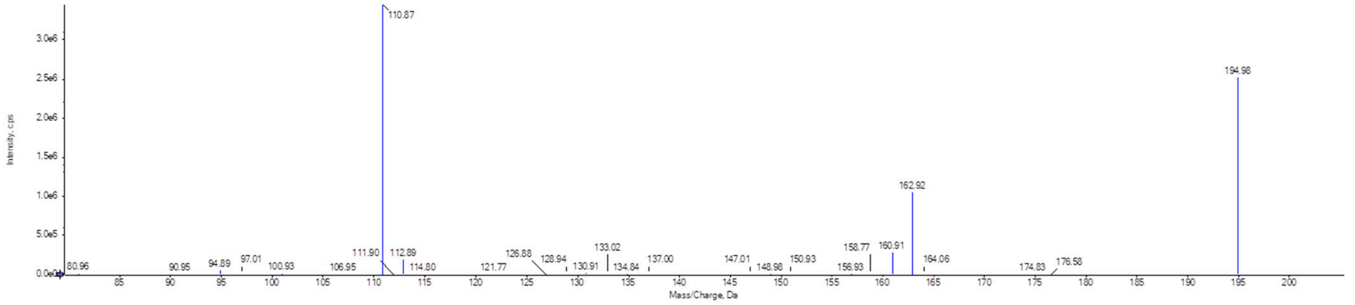

Organophosphate insecticides: Specific metabolites

7. **IMPY**

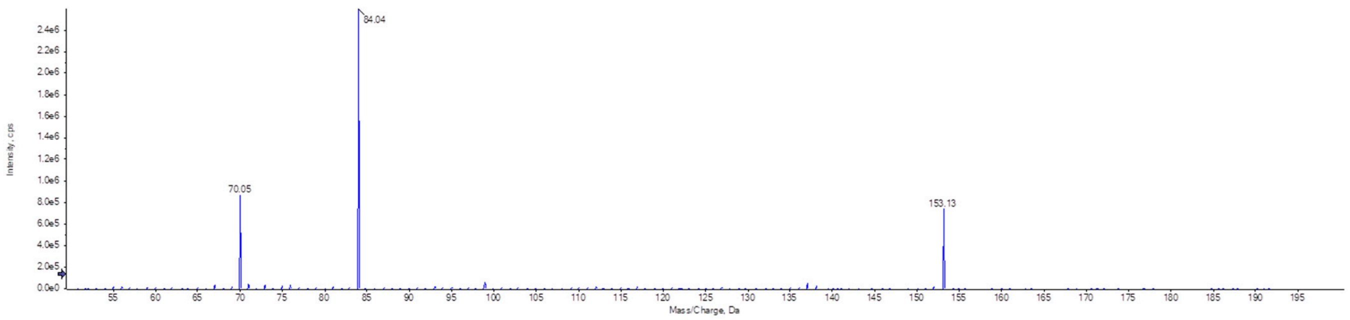

IMPY-$^{13}C_4$

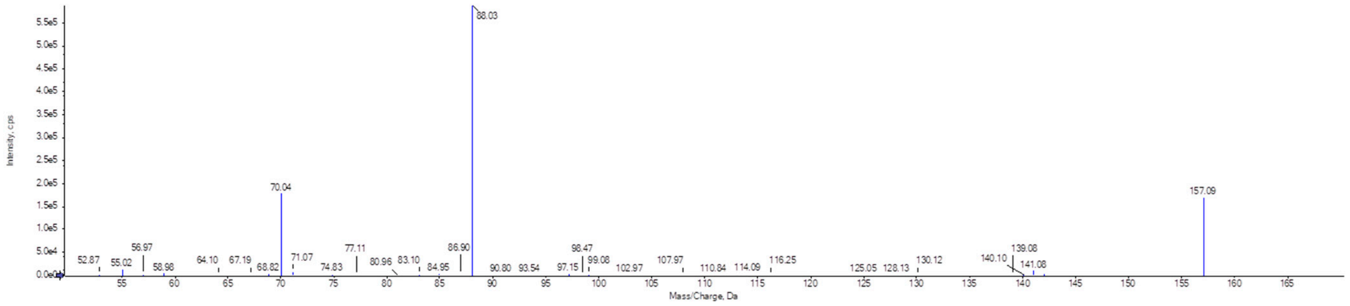

8. DEAMP

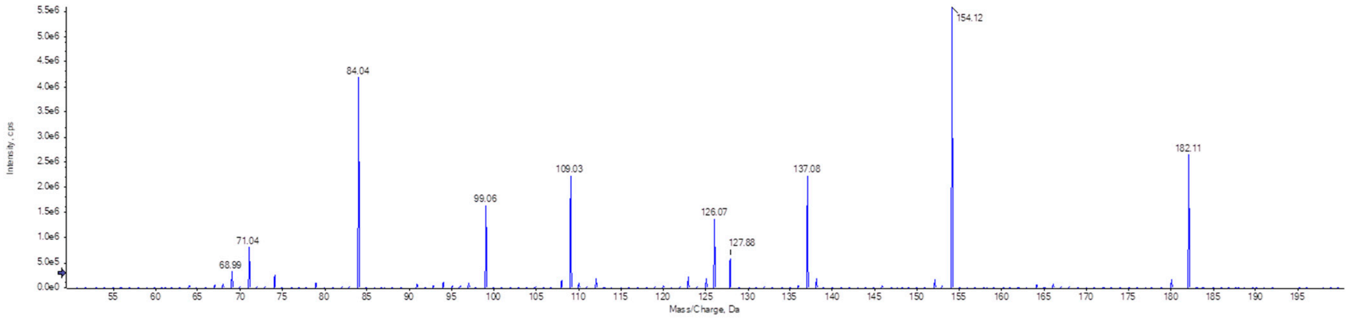

9. MDA

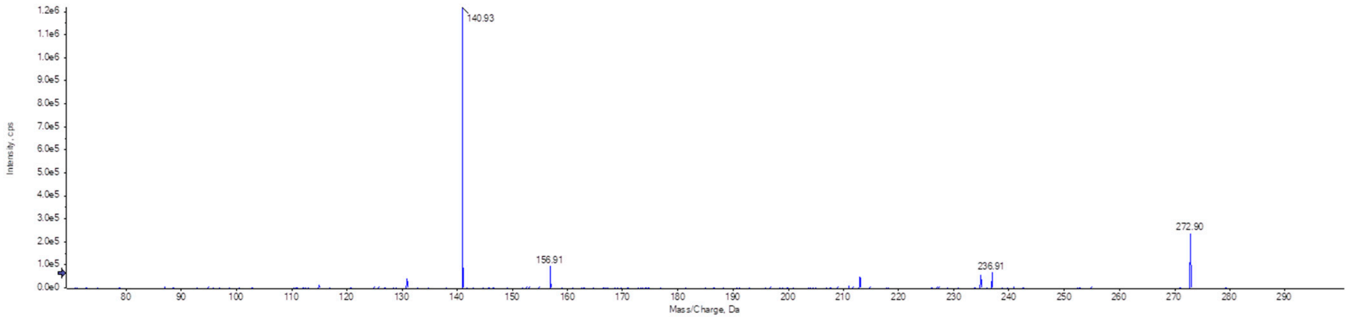

MDA-d_4

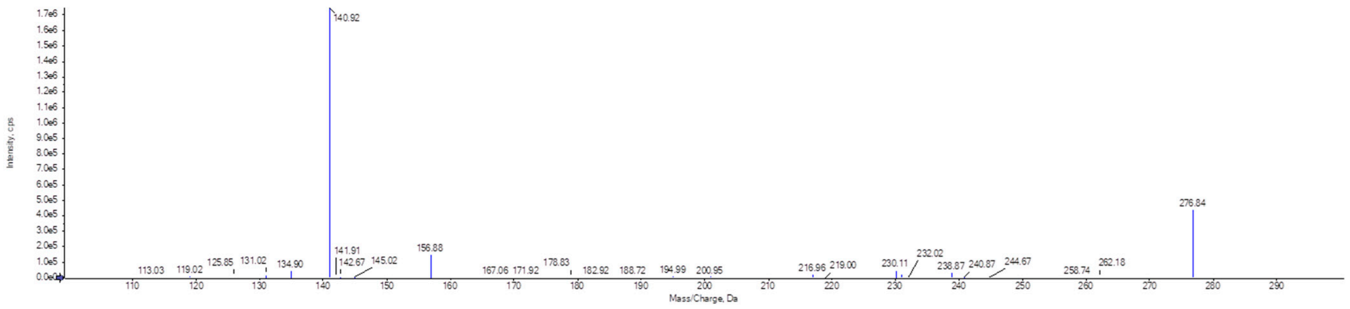

10. PNP

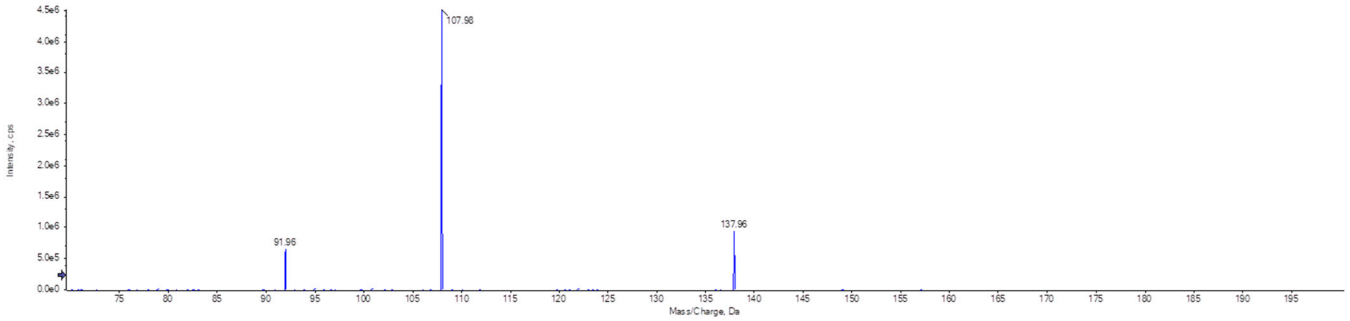

PNP-$^{13}C_6$

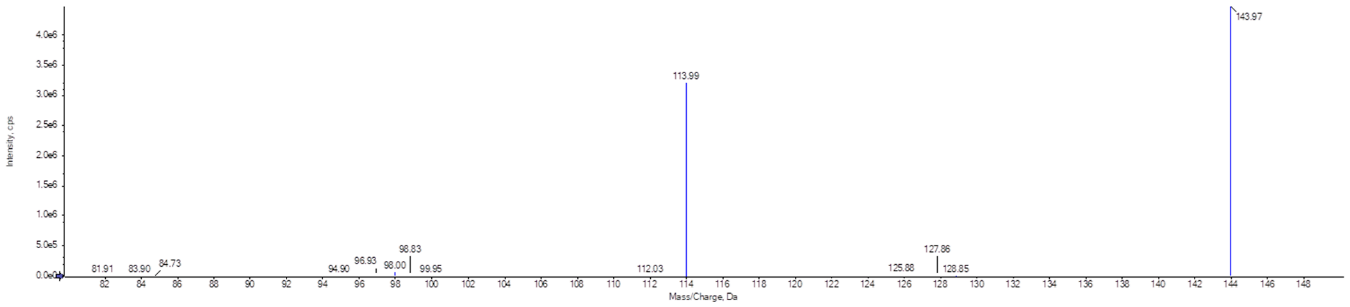

11. TCP

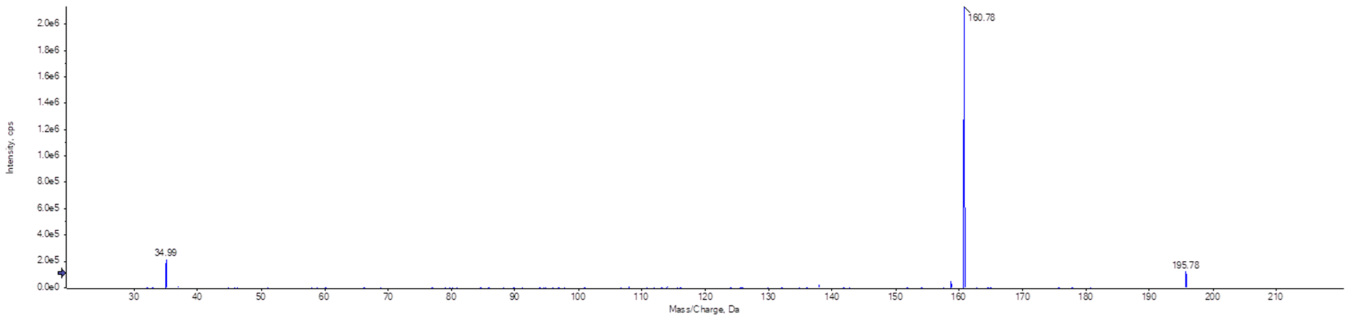

TCP-$^{13}C_3$

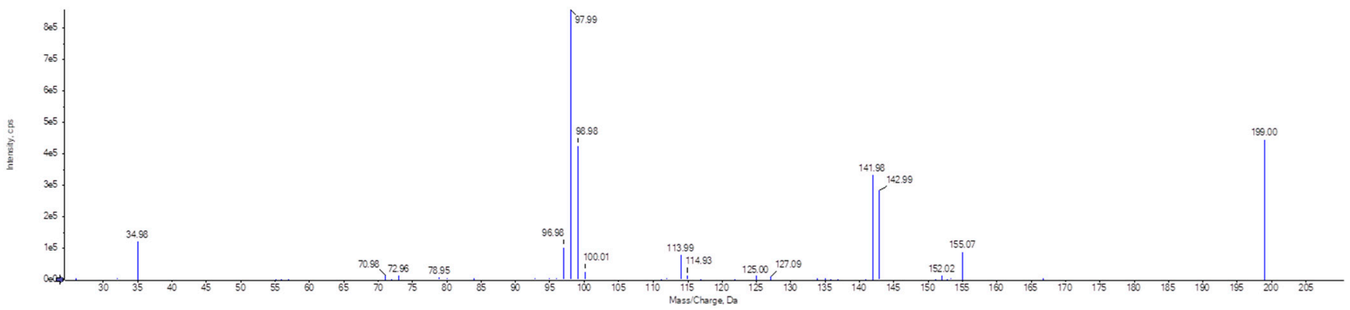

12. TDCCA

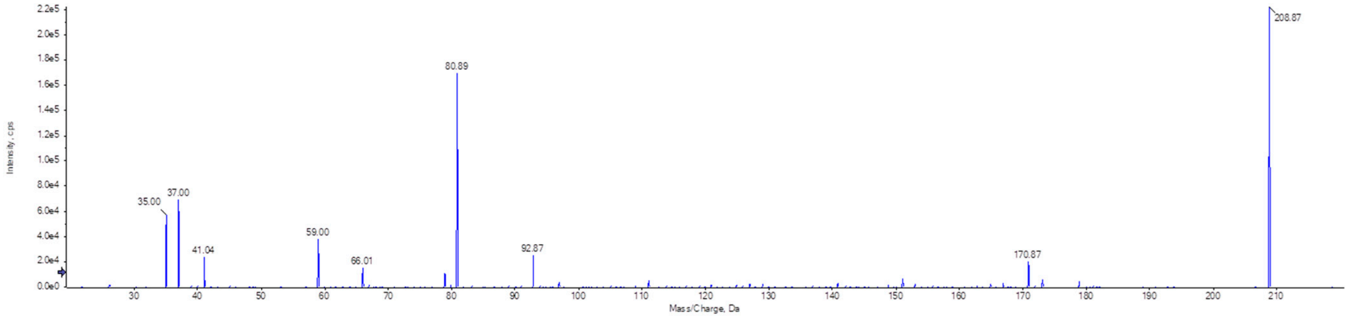

TDCCA-$^{13}C_2$

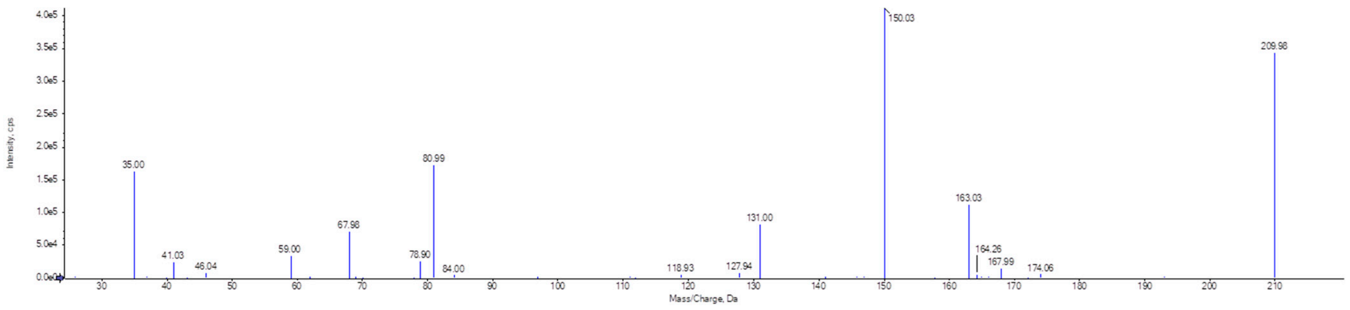

13. PBA

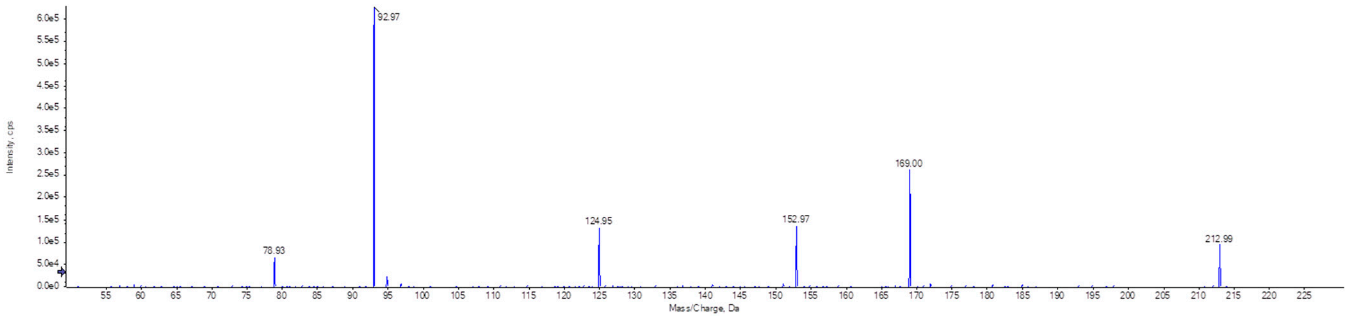

PBA-$^{13}C_6$

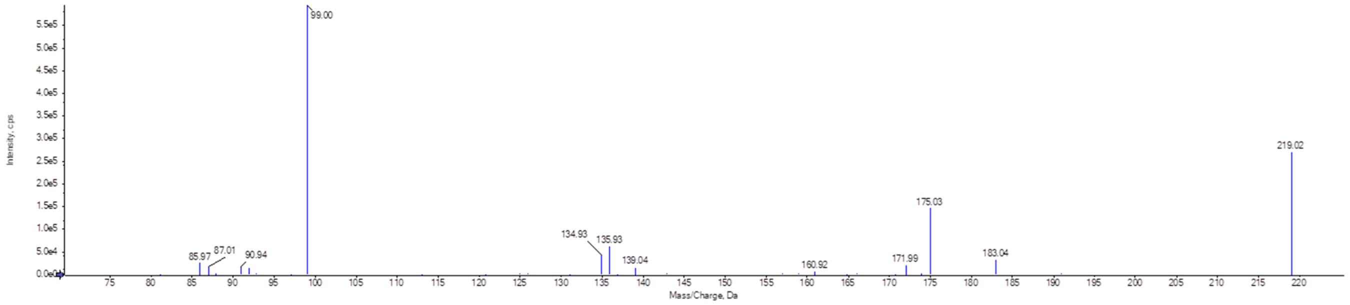

14. FPBA

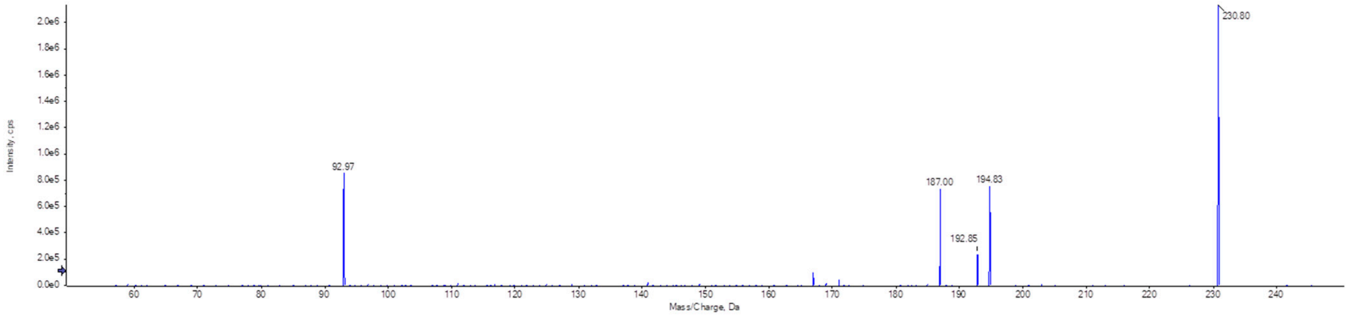

FPBA-$^{13}C_6$

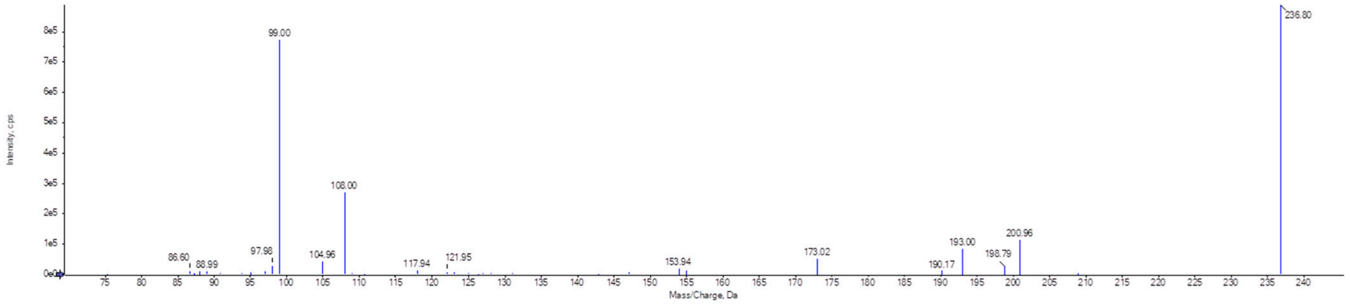

15. CDCCA

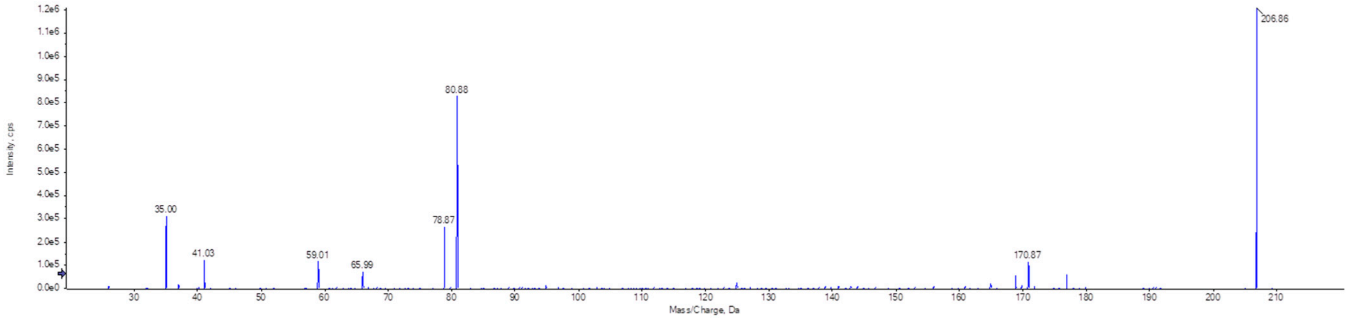

CDCCA-$^{13}C_2$

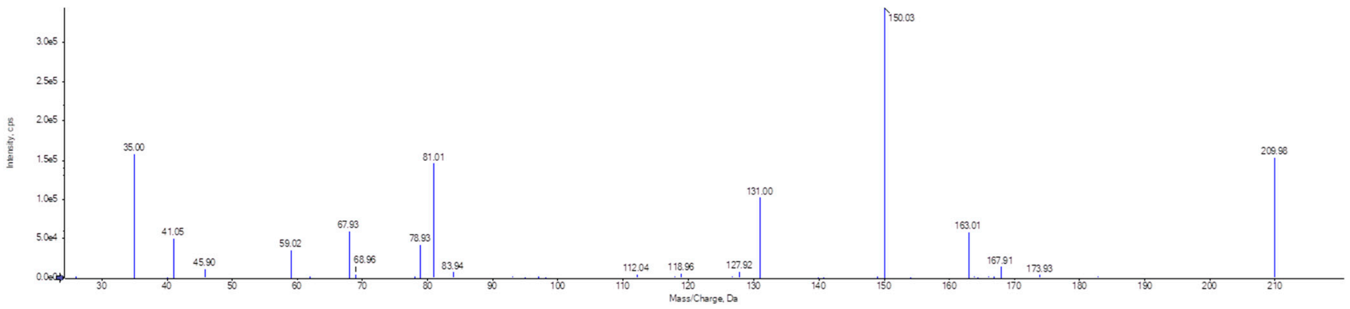

16. CTFCA

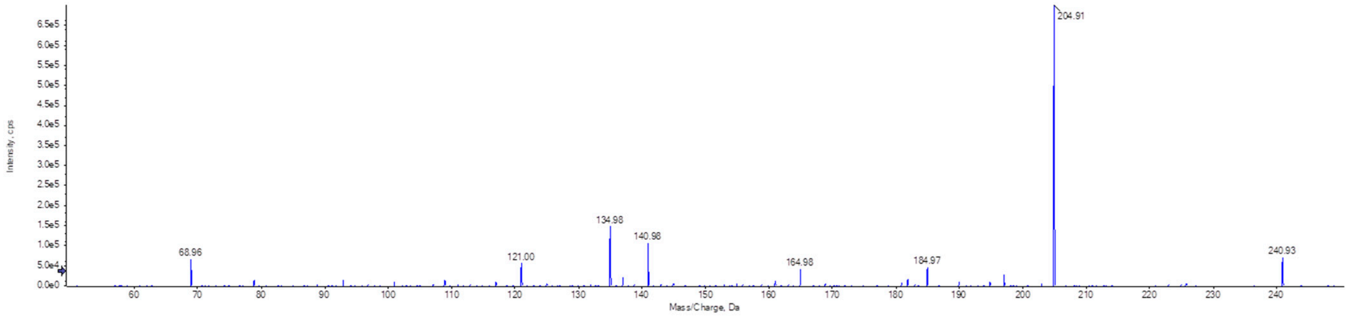

Fungicides and metabolites

17. ETU

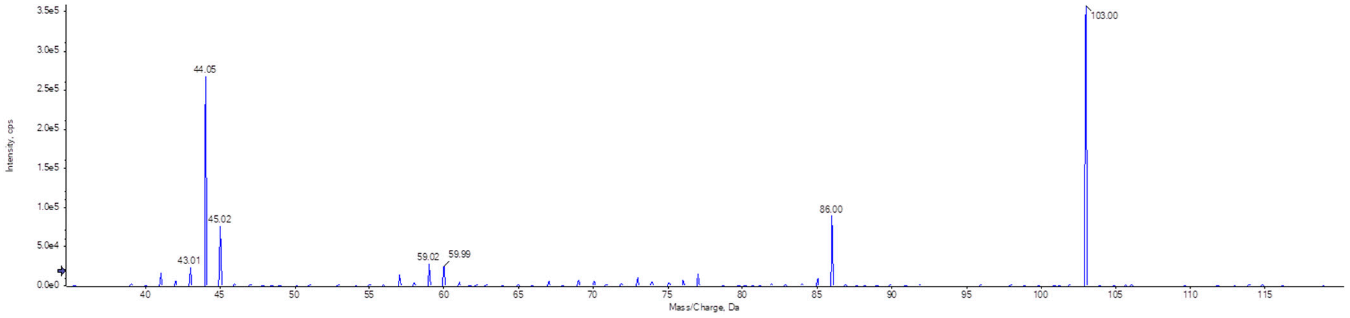

18. PTU

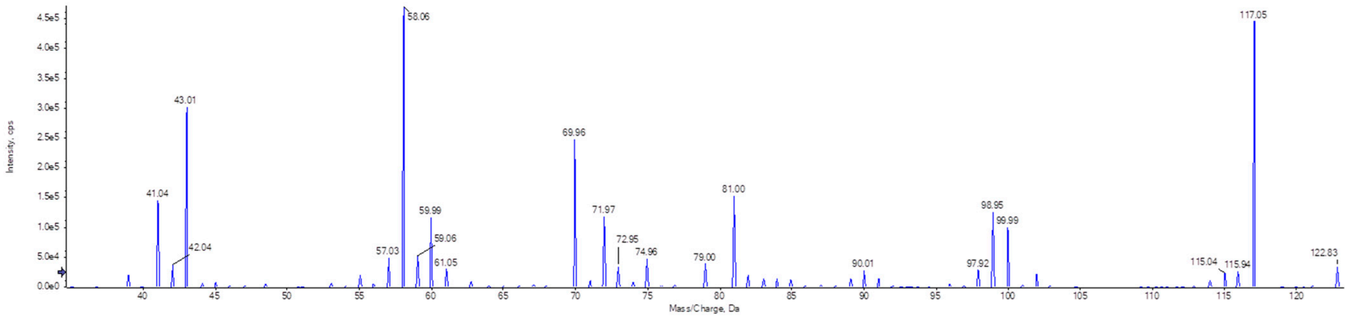

19. THPI

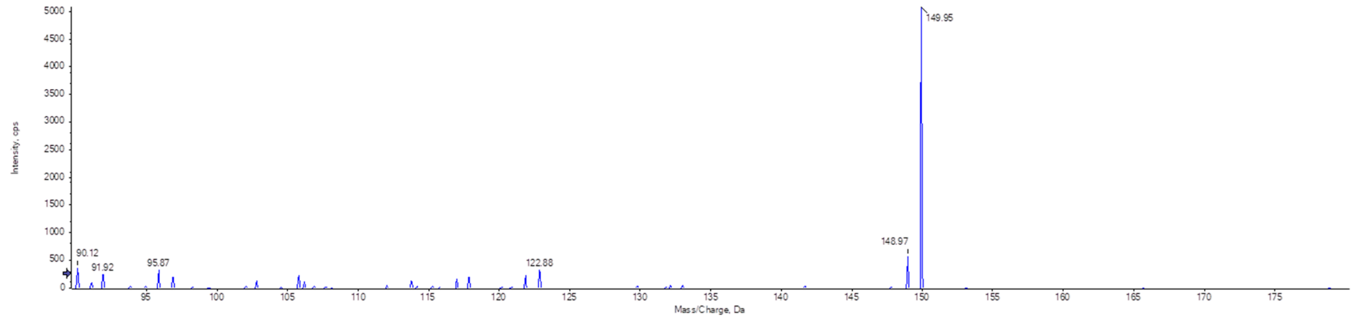

20. MCP4

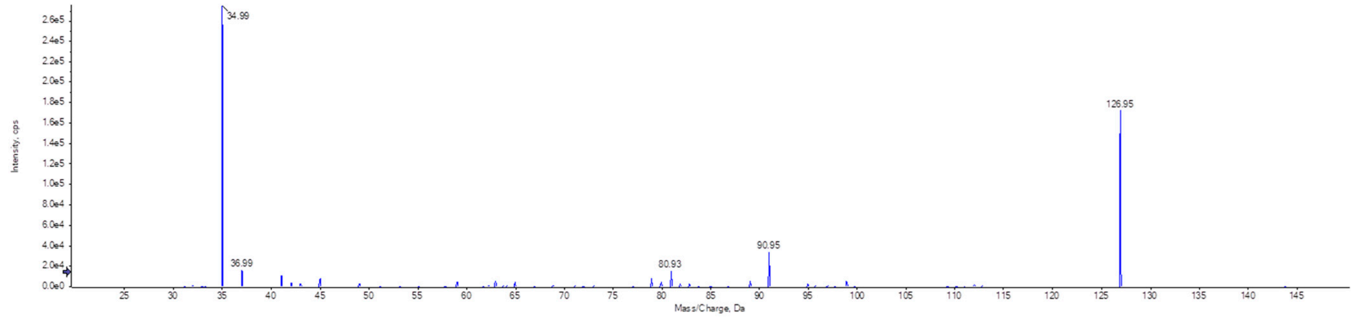

MCP4-d_4

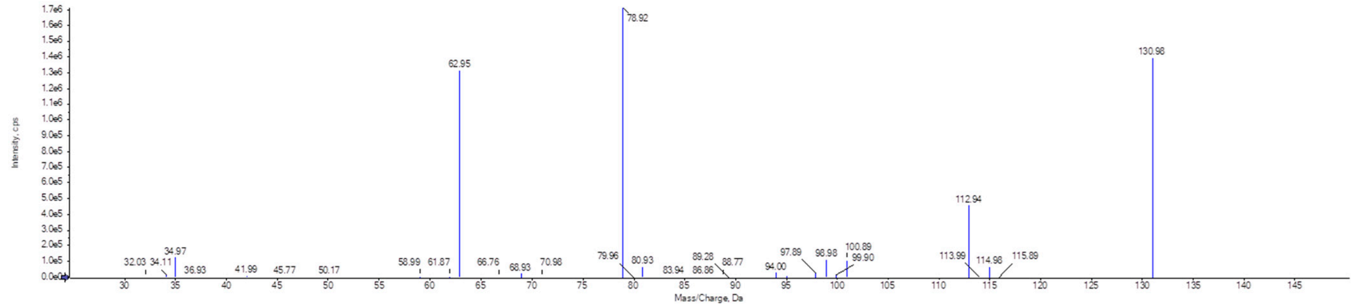

21. OHTBZ

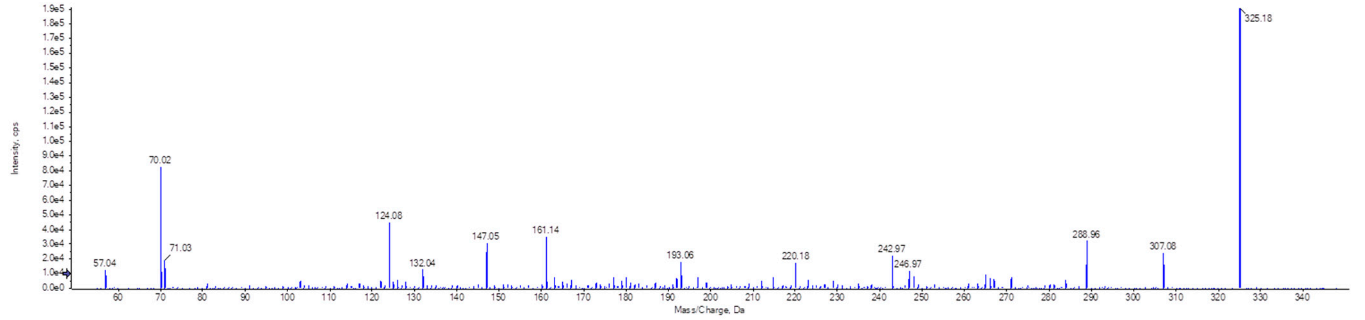

22. AZO

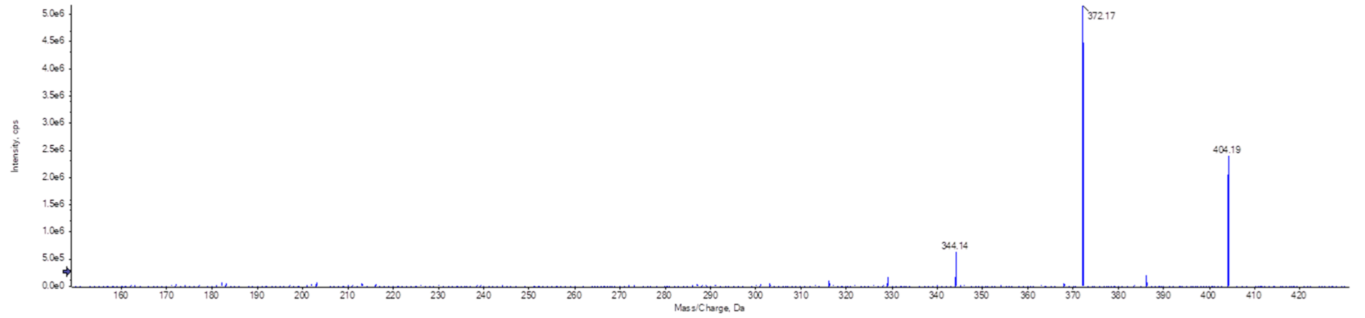

23. PYRM

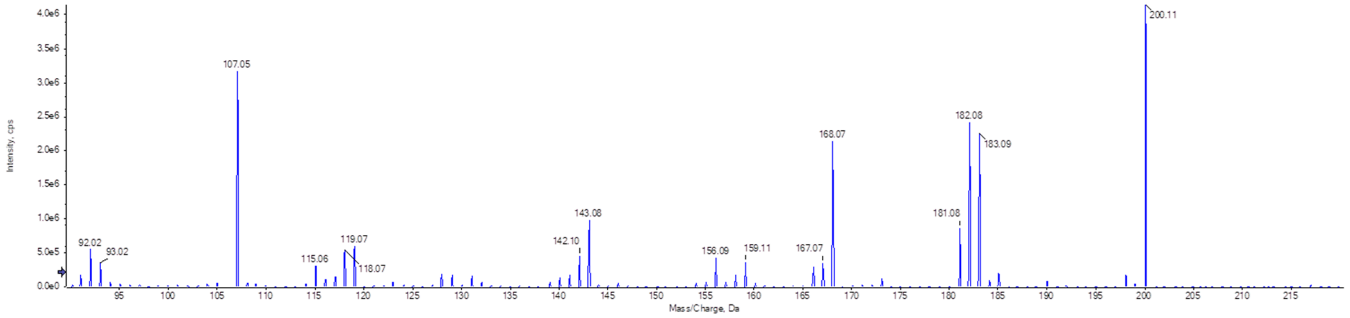

24. TBZ

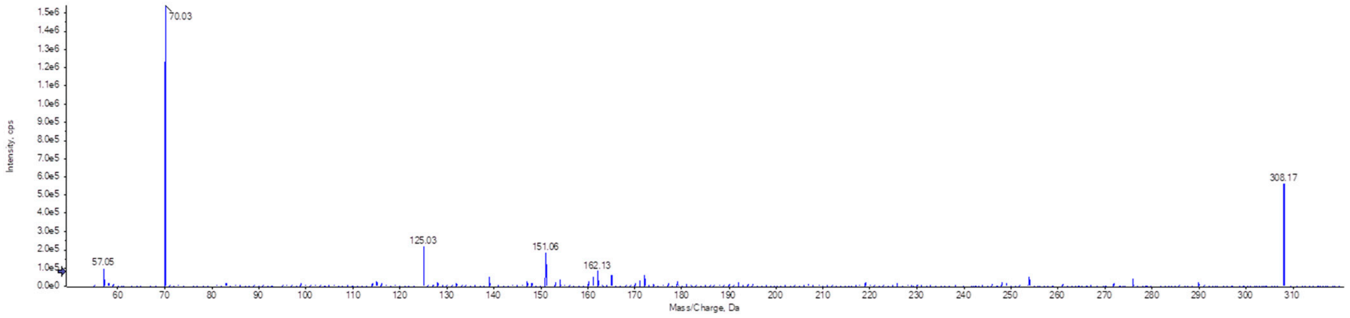

25. PYST

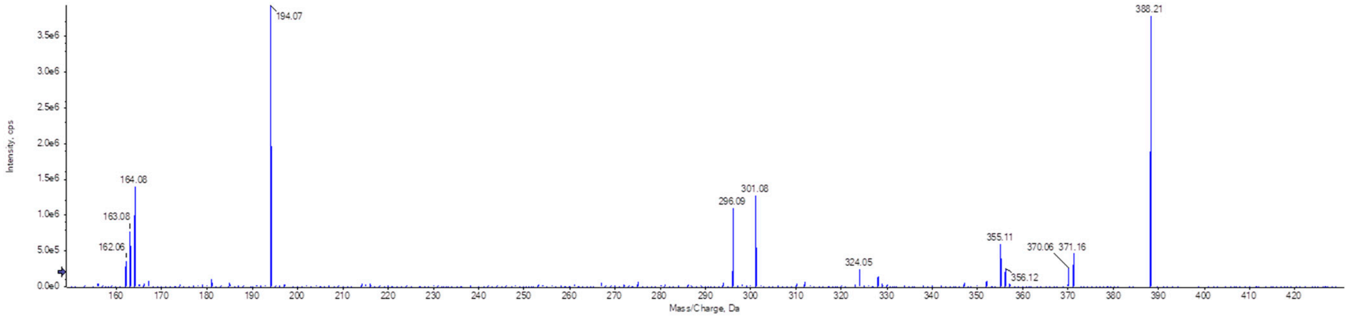

26. PCP

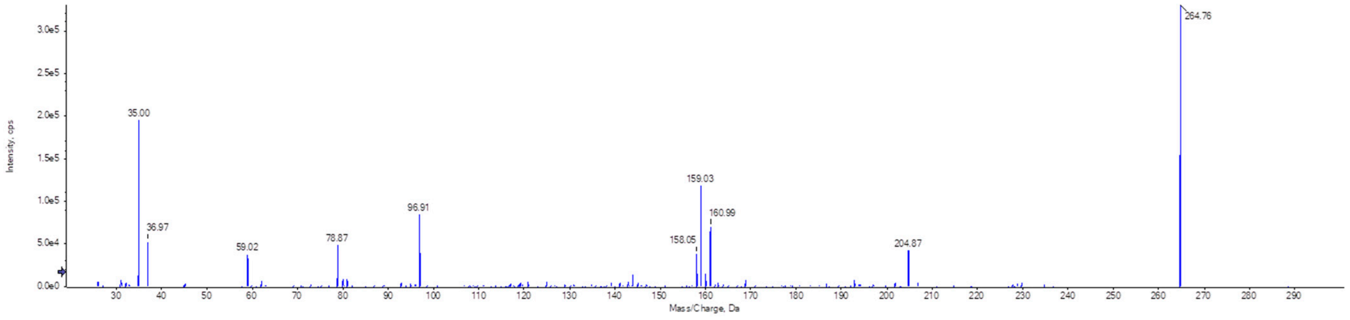

PCP-$^{13}C_6$

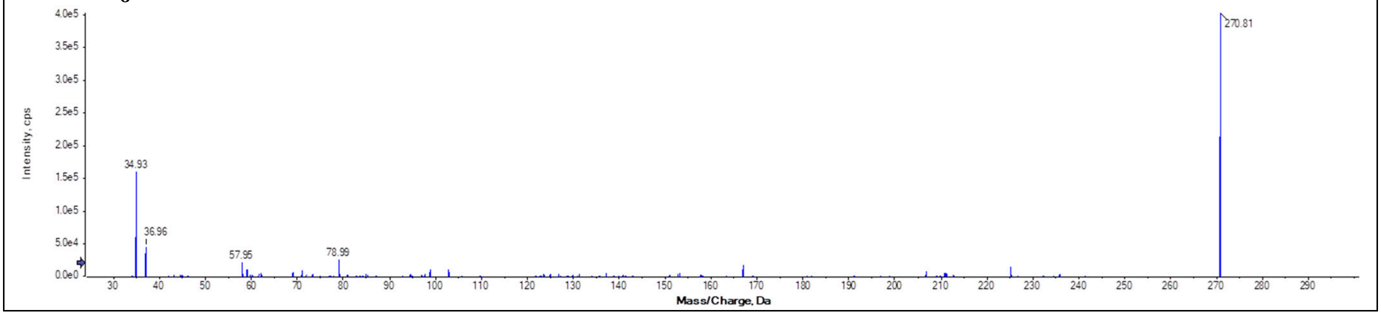

Neonicotinoid insecticides

27. DINF

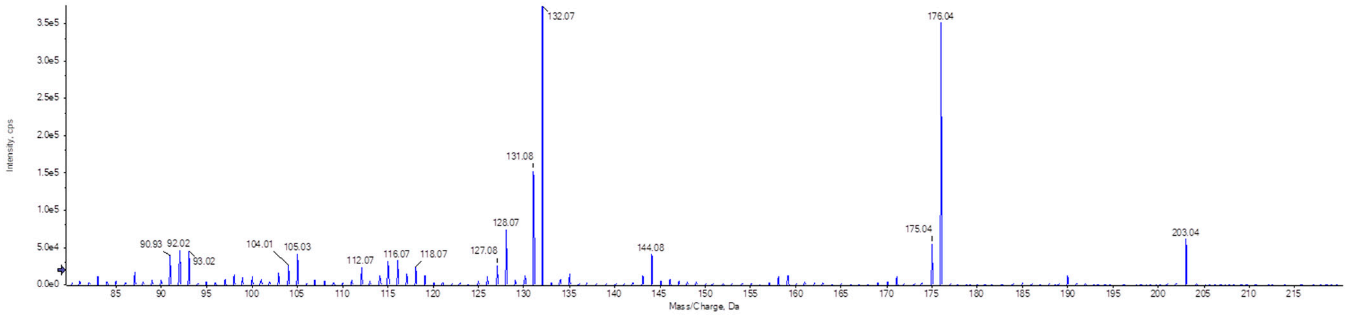

28. THX

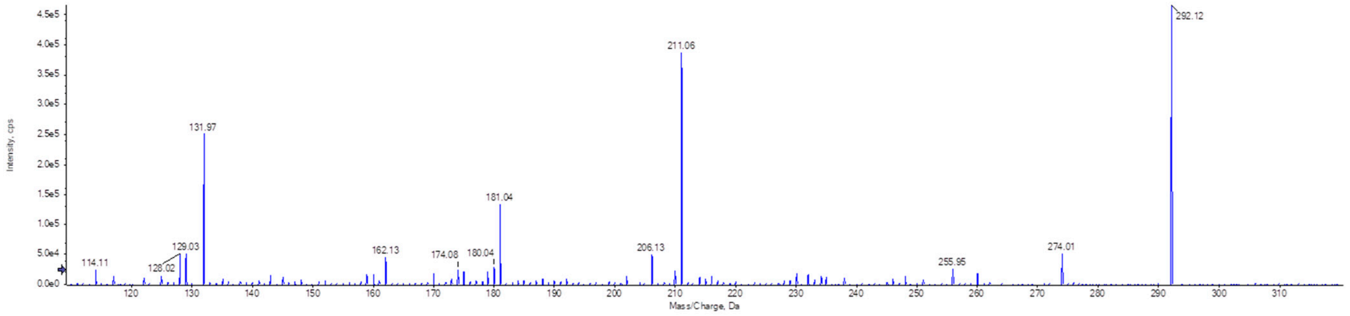

29. FLNC

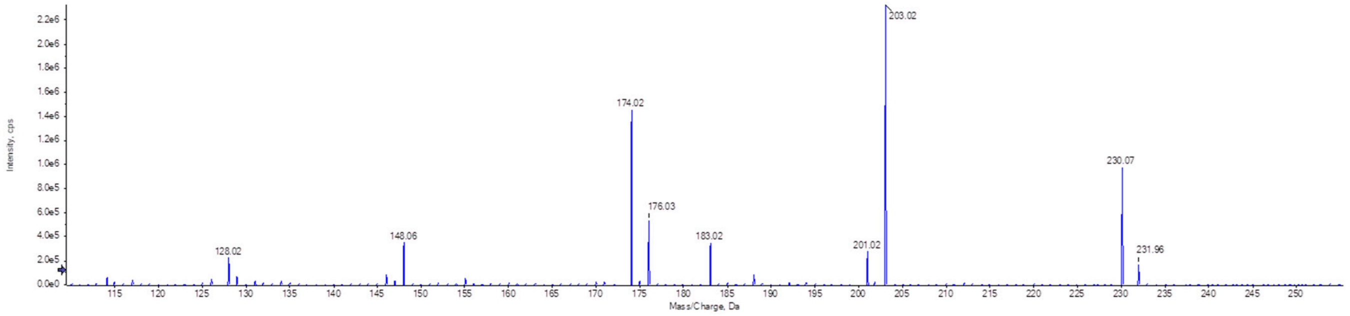

30. OHIMI

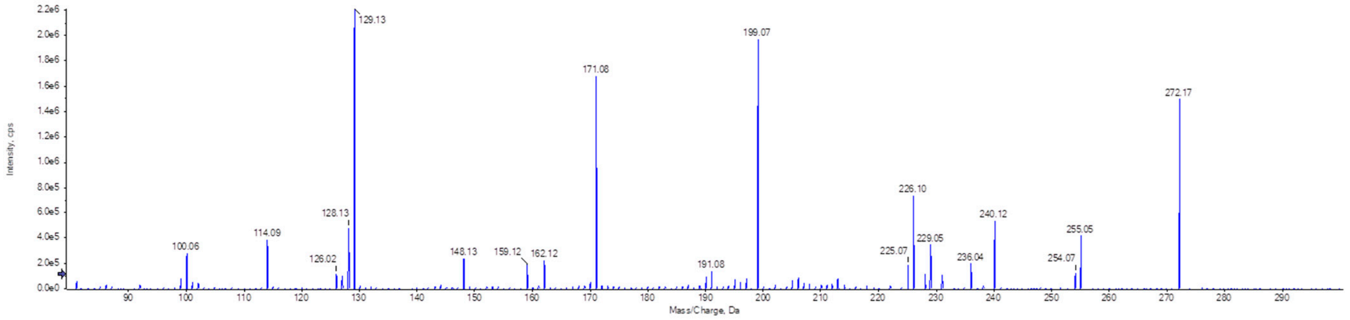

31. CINA6

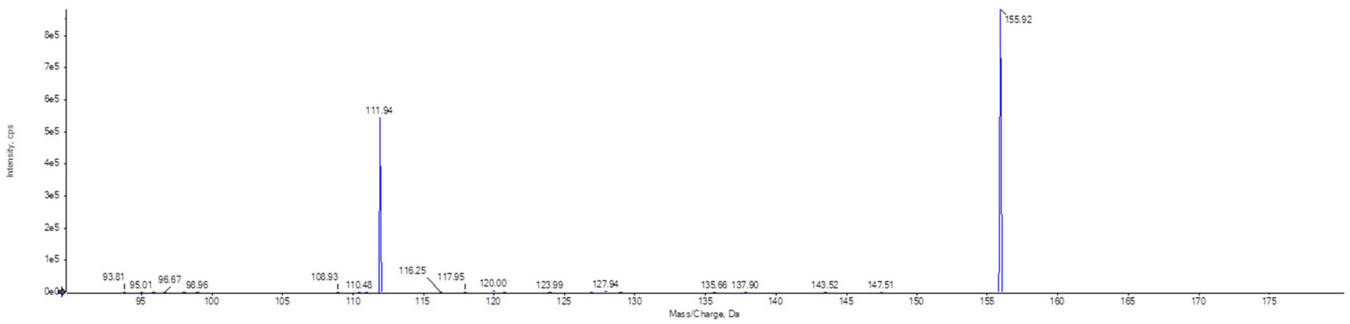

ClNA6-$^{13}C_6$

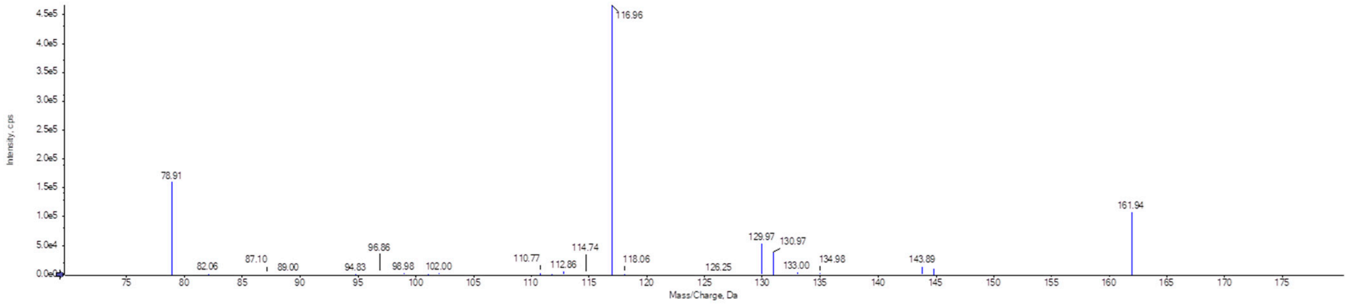

32. **IMI**

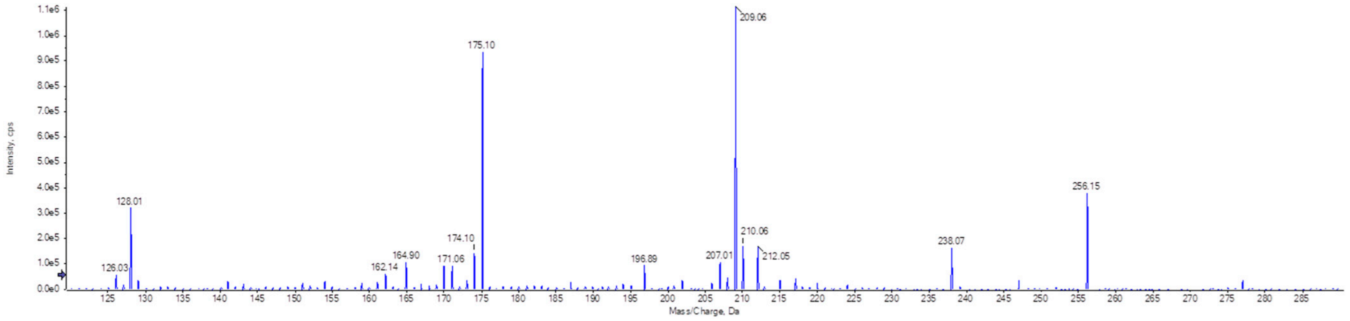

IMI-d_4

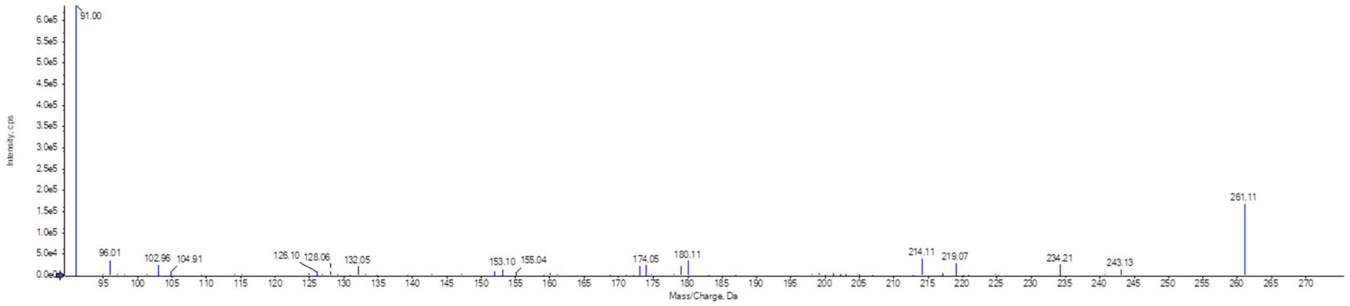

33. **NIT**

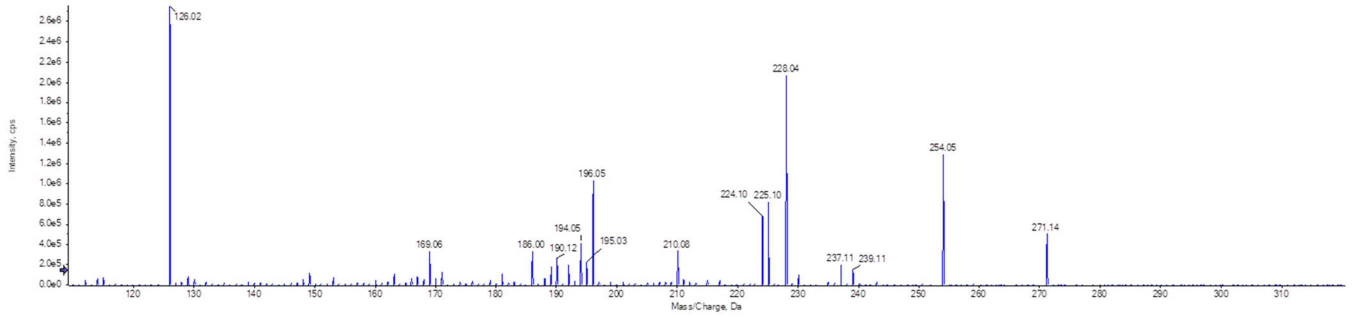

34. NDMA

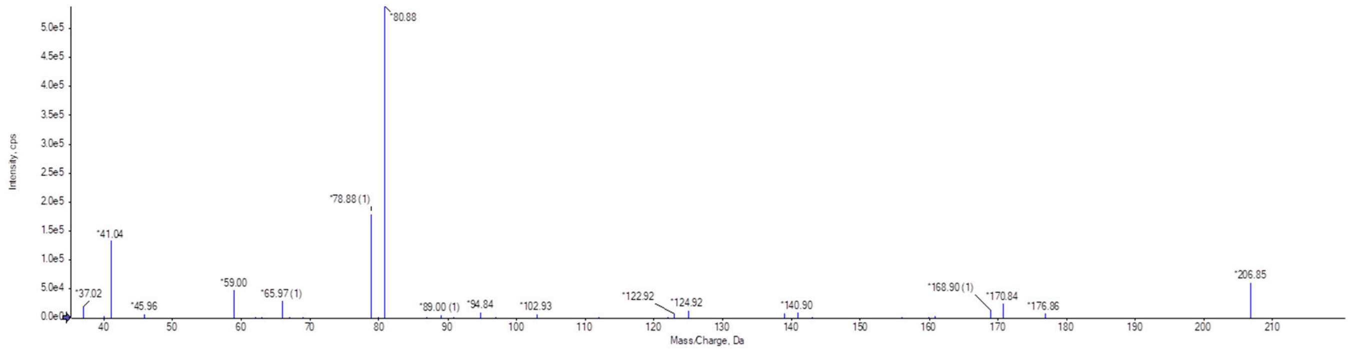

NDMA-$^{13}C_3$

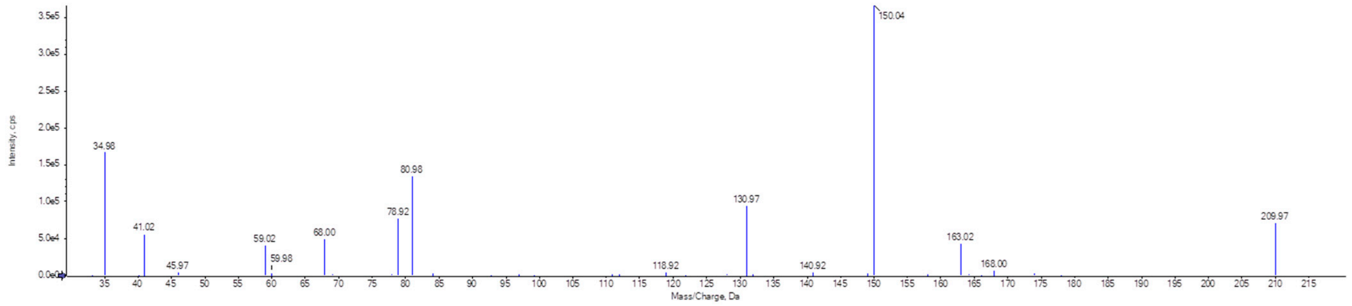

35. TA

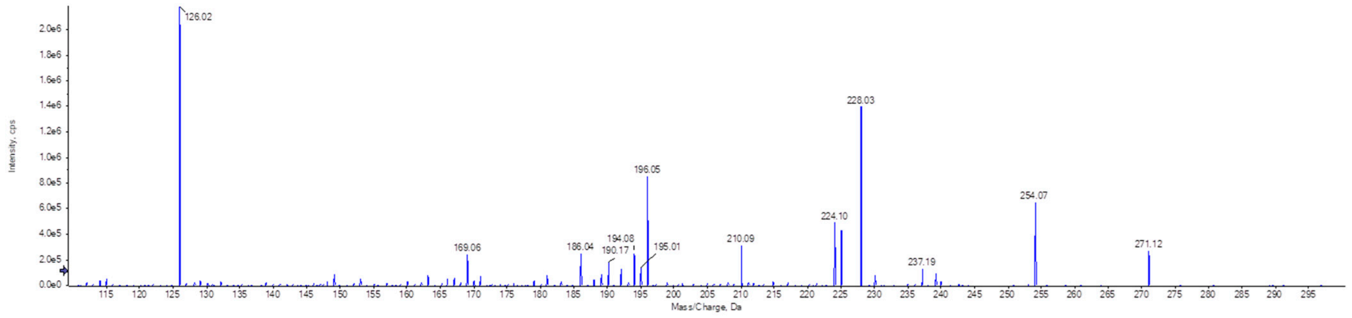

36. CLO

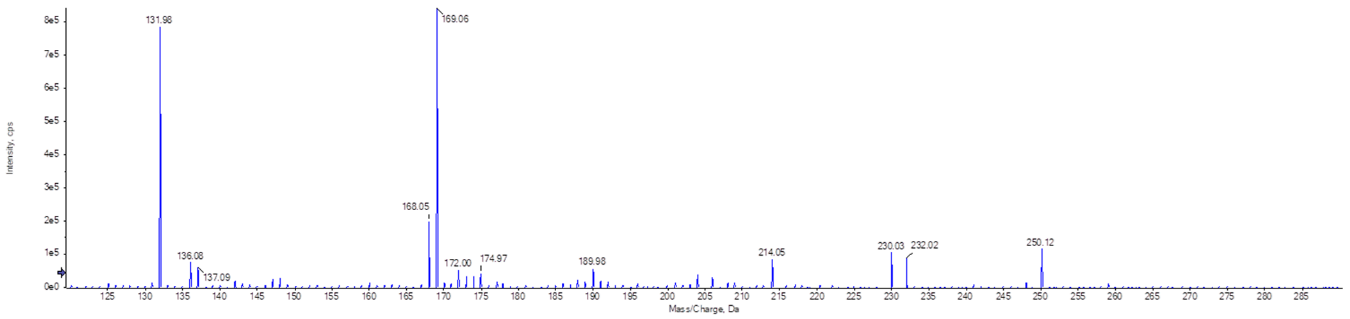

CLO-d_3

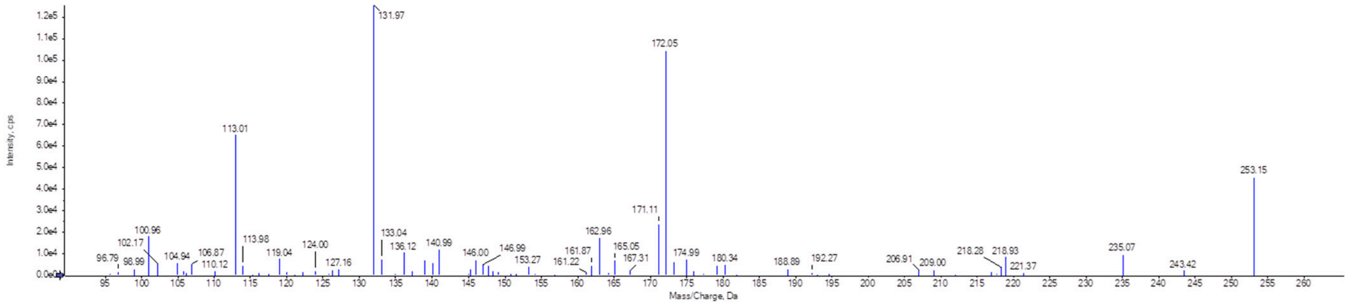

37. ACE

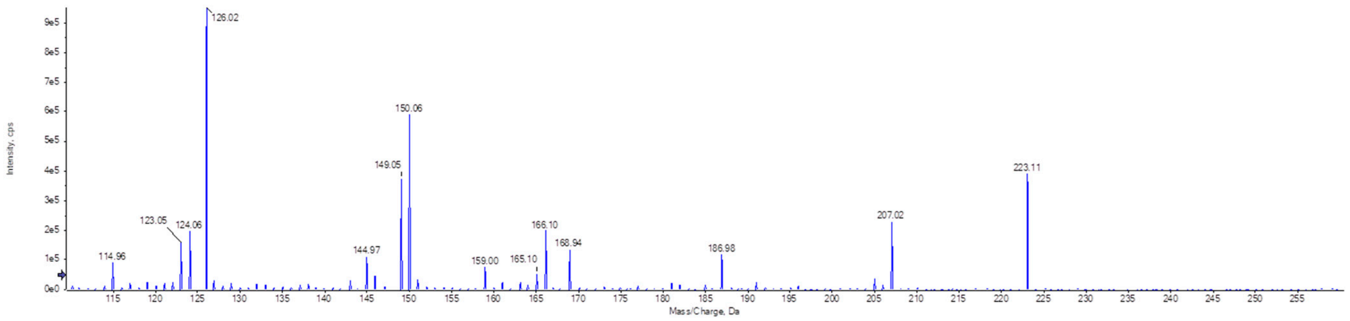

38. SUF

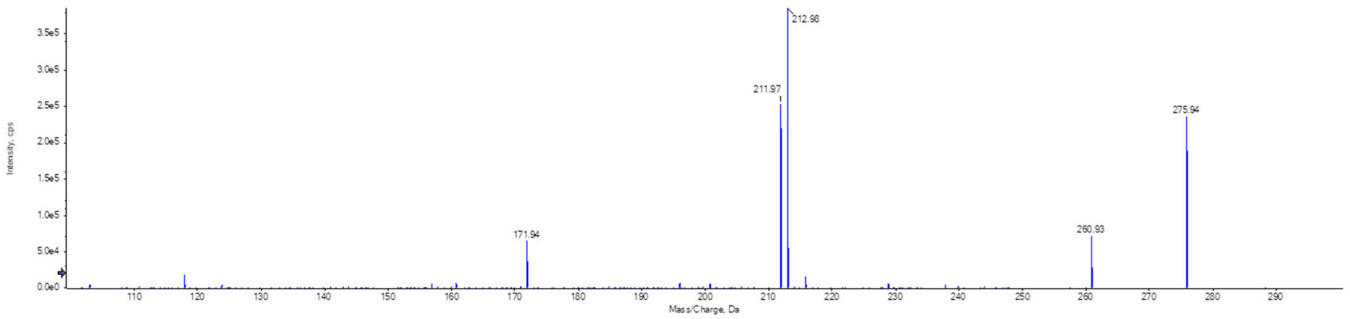

39. THI

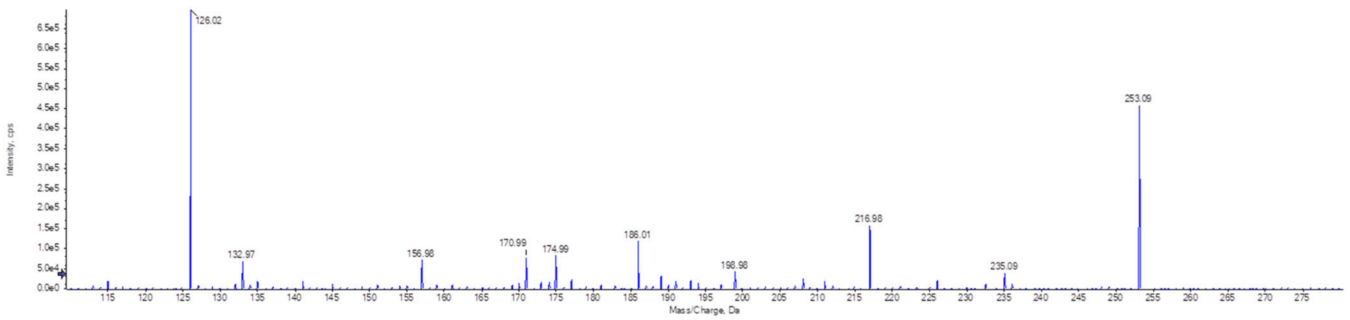

Herbicides and metabolites

40. D24

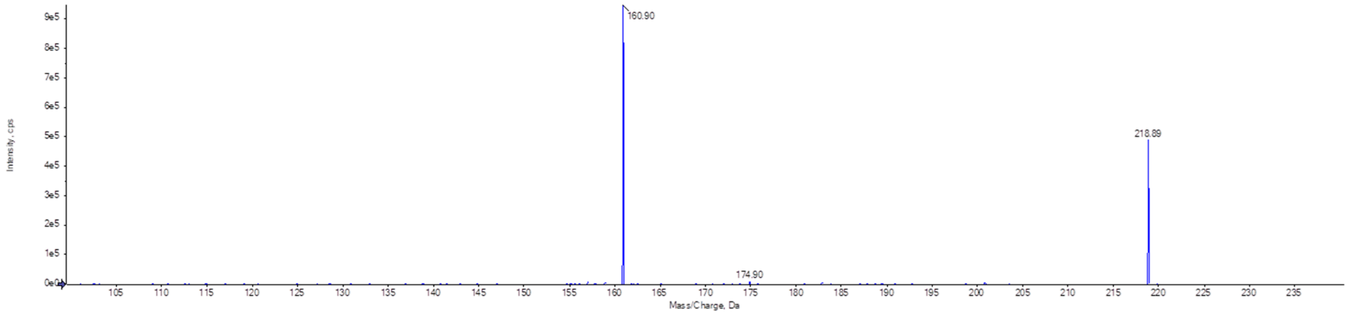

D24-$^{13}C_6$

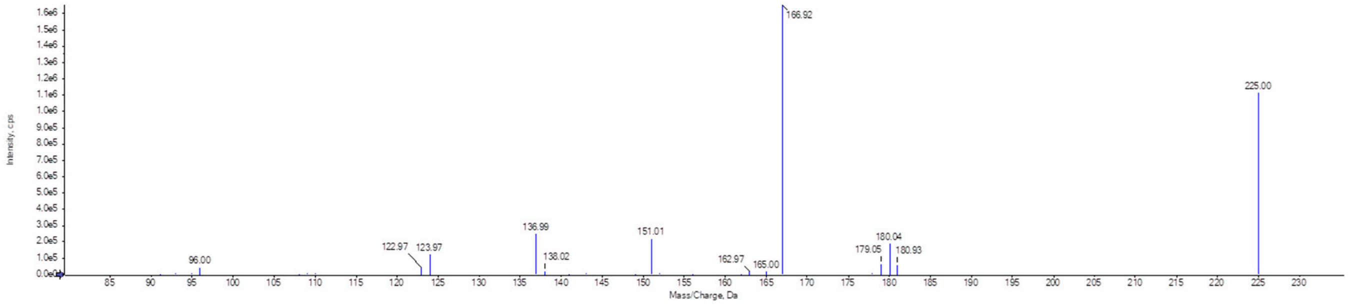

41. ATZ

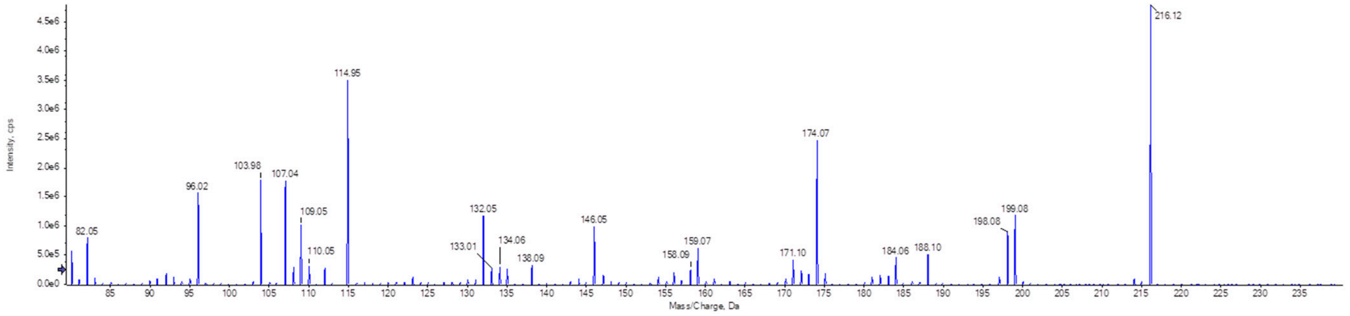

42. T245

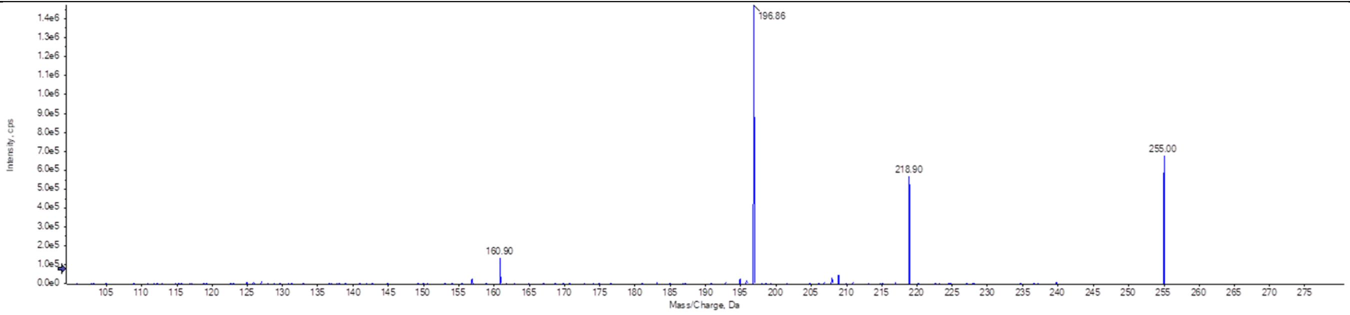

T245-$^{13}C_4$

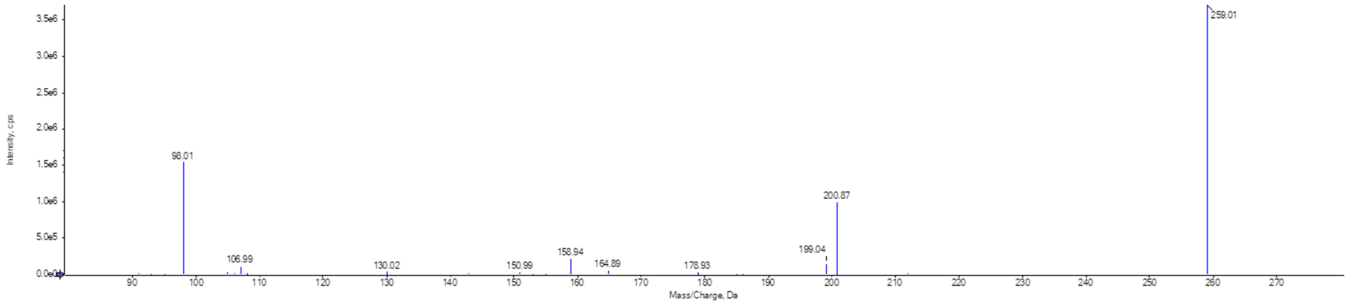

Insect repellents and metabolites

43. ECBA

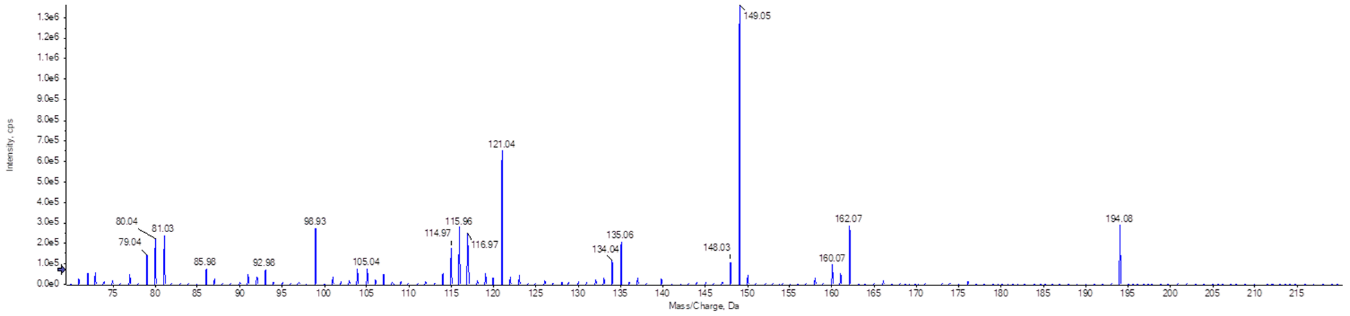

ECBA-d_5

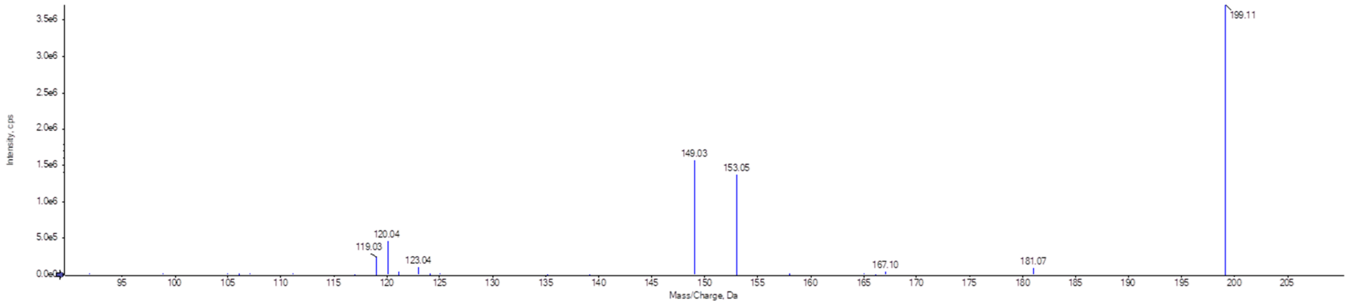

44. DHMB

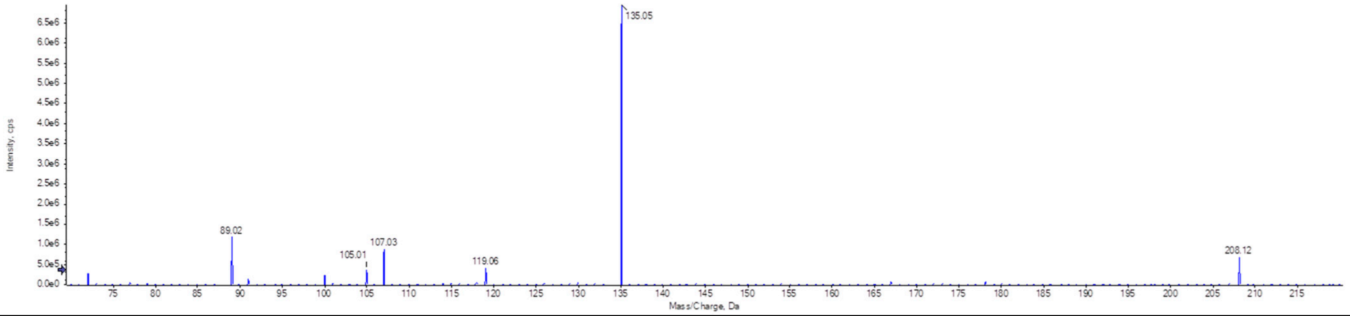

45. DCBA

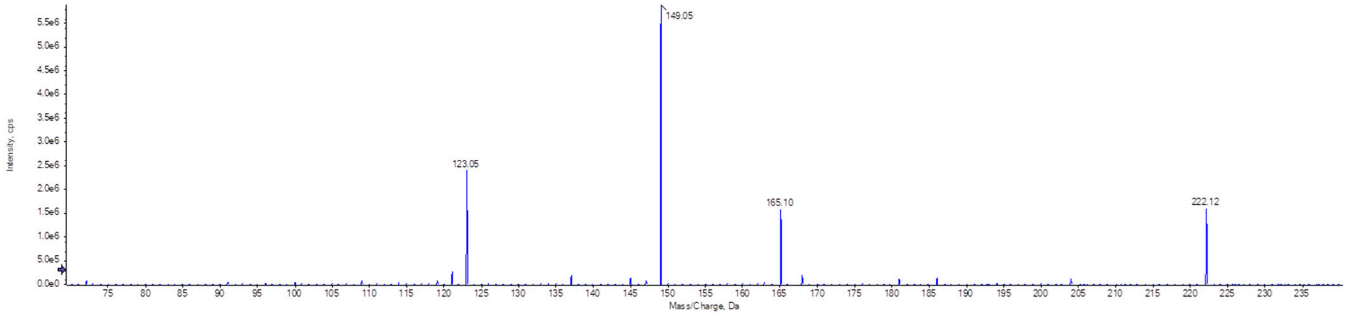

46. DEET

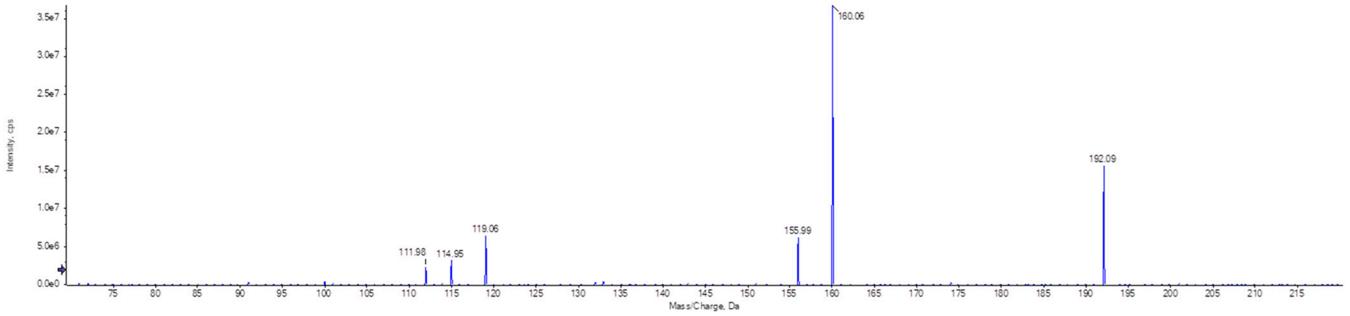

DEET-d_6

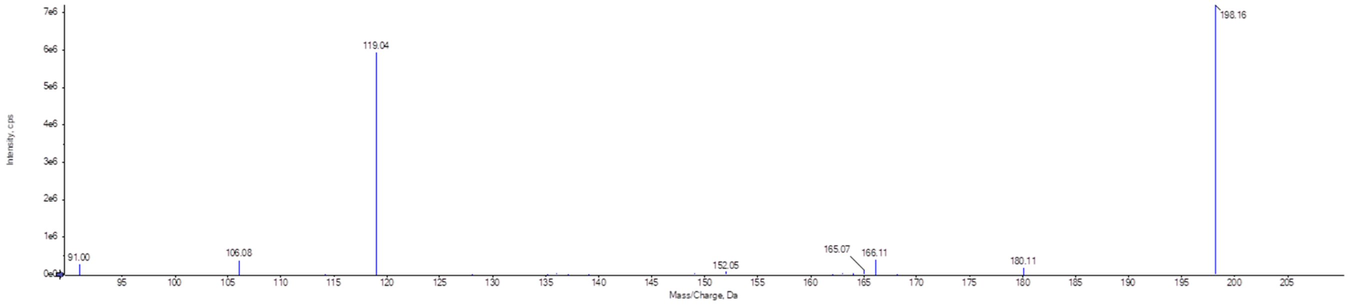

Organochlorine pesticide metabolites

47. TCP246

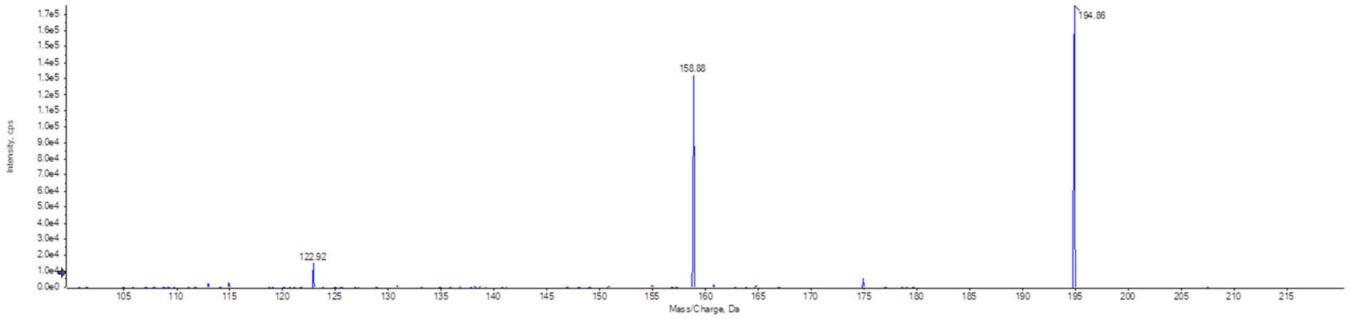

TCP246-$^{13}C_6$

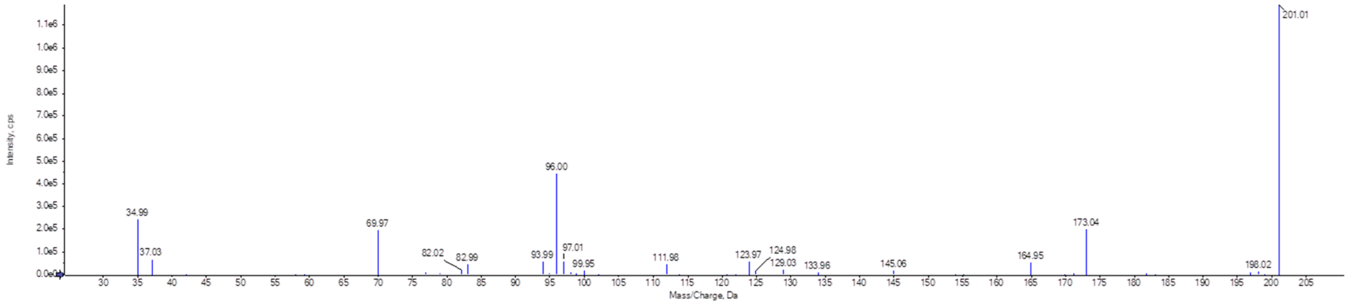

48. TECP2356

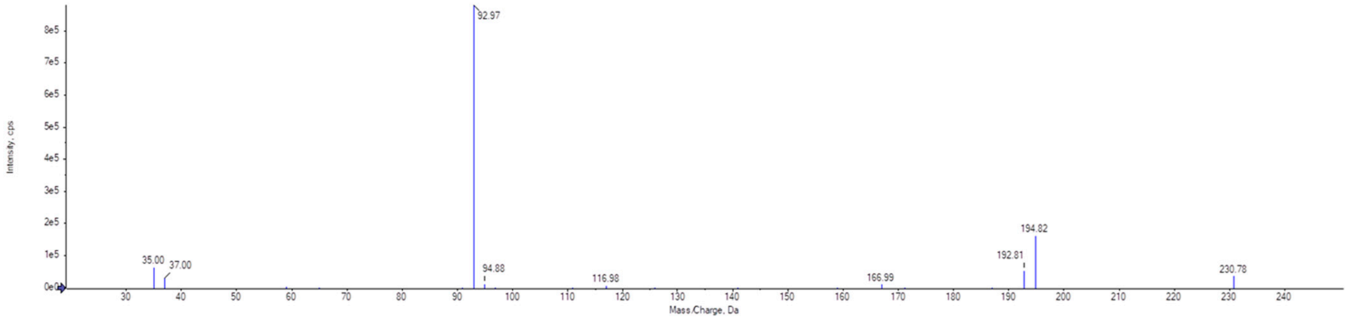

TECP2356-$^{13}C_6$

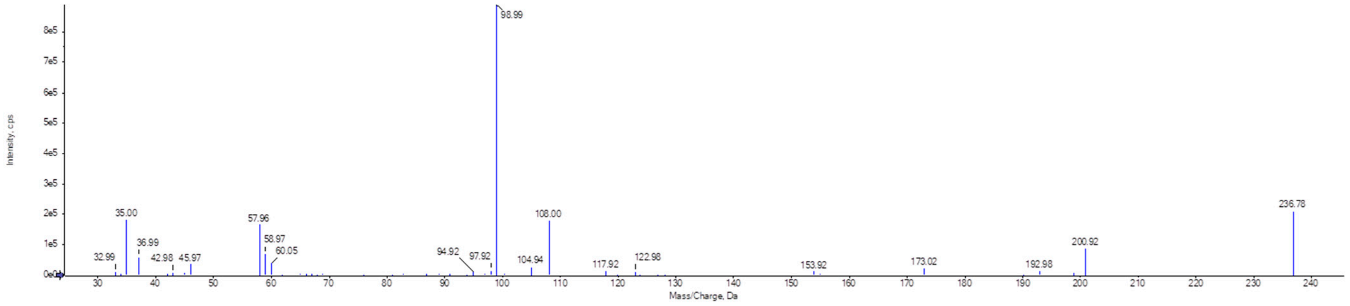

Plant growth regulators

49. CCC

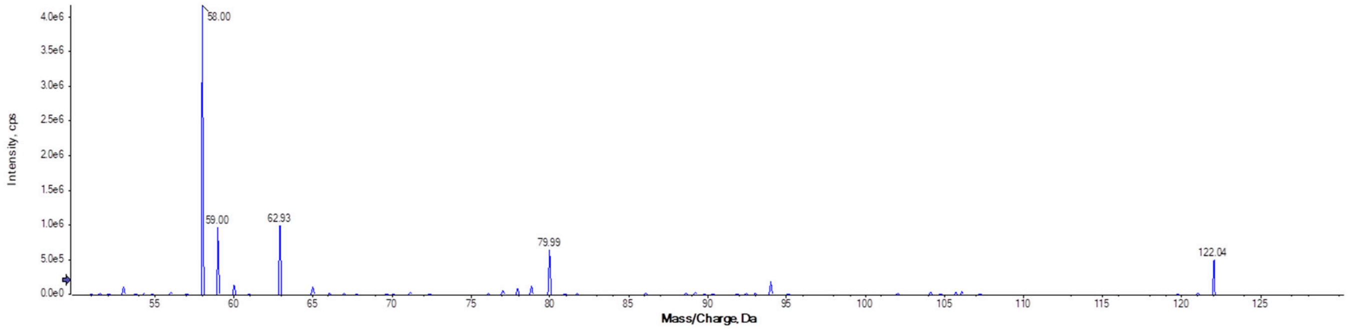

50. MQ

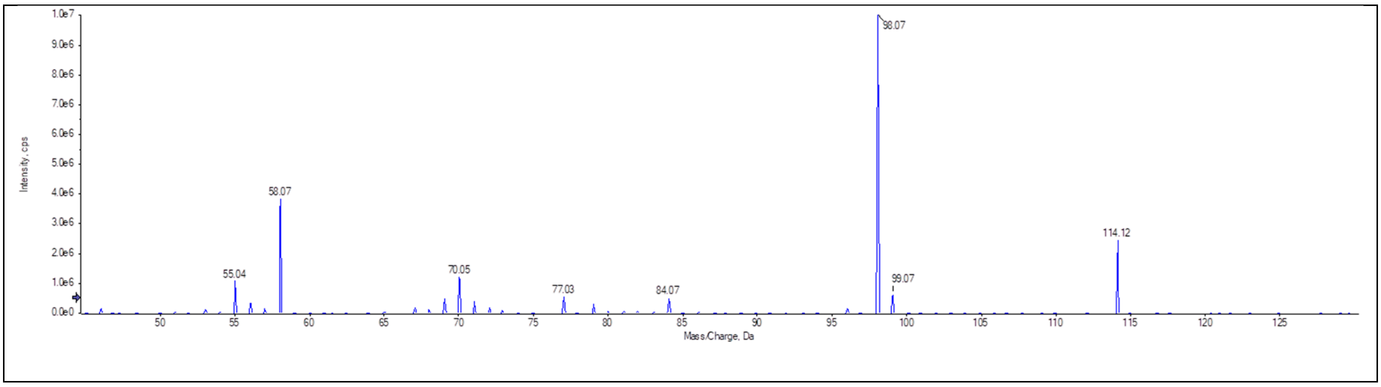

Intensity, cps
1.0e7
9.0e6
8.0e6
7.0e6
6.0e6
5.0e6
4.0e6
3.0e6
2.0e6
1.0e6
0.0e0
98.07
58.07
114.12
55.04
70.05
99.07
77.03
84.07
50
55
60
65
70
75
80
85
90
95
100
105
110
115
120
125
Mass/Charge, Da
